# Real‐world characteristics and outcomes of patients with multiple myeloma receiving second‐line treatment in England

**DOI:** 10.1002/jha2.1058

**Published:** 2024-12-05

**Authors:** Sally Moore, Laura Cornic, Christina‐Jane Crossman‐Barnes, Sophie Jose, Zeyad Khalaf, Kwee Yong, Megan Soutar, Philip Woods

**Affiliations:** ^1^ Department of Haematology University Hospitals Bristol and Weston NHS Foundation Trust Bristol UK; ^2^ GSK London UK; ^3^ Health Data Insight CIC Cambridge UK; ^4^ National Disease Registration Service NHS England London UK; ^5^ Department of Haematology University College Hospital London UK

**Keywords:** National Cancer Registration and Analysis Service, real‐world evidence, relapsed/refractory multiple myeloma

## Abstract

**Introduction:**

Despite recent advances in first‐line therapies for multiple myeloma (MM), most patients relapse or become refractory, underscoring the need for effective second‐line (2L) regimens for relapsed/refractory MM (RRMM).

**Methods:**

This study describes the real‐world baseline characteristics, treatment patterns and clinical outcomes of adult patients diagnosed with MM between 2013 and 2020 using data collated by the National Cancer Registration and Analysis Service (NCRAS) of the National Health Service in England. The study cohorts were broadly aligned to the eligibility criteria of the ongoing DREAMM‐7 (D7) and DREAMM‐8 (D8) clinical trials. We focus on lenalidomide‐exposed/refractory patients who received daratumumab–bortezomib–dexamethasone (DaraVd) at 2L in both cohorts.

**Results:**

In the D7‐like cohort, the lenalidomide‐exposed (*n *= 282) and lenalidomide‐refractory (*n *= 143) patients who received DaraVd at 2L had a median (95% confidence interval [CI]) time to next treatment or death (TTNTD) of 15.1 (12.6–22.4) and 10.3 (7.4–13.9) months, respectively. In the D8‐like cohort, the lenalidomide‐exposed (*n *= 269) and lenalidomide‐refractory (*n *= 148) patients who received DaraVd at 2L had a median (95% CI) TTNTD of 14.5 (11.7–19.7) and 10.0 (7.3–13.7) months, respectively.

**Conclusion:**

Patients with RRMM in England receiving DaraVd at 2L have poor clinical outcomes, highlighting the urgent need for new therapies, particularly for lenalidomide‐refractory patients.

## 1 INTRODUCTION

Multiple myeloma (MM) is a malignant plasma cell disorder accounting for up to 1.8% of all neoplasms, with an estimated incidence of 4.5–6 cases per 100,000 people per year in Europe [1]. In the United Kingdom, MM accounts for approximately 6000 new diagnoses and 3000 deaths annually, and the 10‐year survival rate is 29% [2]. Over the past decade, novel therapies have led to improvements in clinical outcomes of patients with MM; however, most patients relapse or become refractory to treatment [3]. In a study across seven European countries, 61% of patients with MM received second‐line (2L) treatment after relapse, 38% received third‐line (3L), 15% received fourth‐line (4L) and 1% received fifth‐line (5L) treatments [4].

First‐line (1L) treatment for patients with MM includes high‐dose chemotherapy with autologous stem cell transplantation (ASCT), where eligible [1, 5]. In the United Kingdom, the National Institute for Health and Care Excellence (NICE) guidelines recommend treatment with bortezomib–dexamethasone (Vd), with or without thalidomide, for induction therapy prior to ASCT, or daratumumab–bortezomib–thalidomide–dexamethasone as both induction and consolidation, and lenalidomide as maintenance therapy after ASCT [6, 7, 8, 9]. For patients ineligible for ASCT, treatment at 1L is thalidomide‐, bortezomib‐ or lenalidomide‐based [5, 10]. Lenalidomide–dexamethasone (Rd) is recommended for patients for whom thalidomide is either contraindicated or not tolerated [10]. On 25 October 2023, the daratumumab–Rd (DaraRd) triplet regimen gained NICE approval for 1L treatment in ASCT‐ineligible patients [11].

In the 2L setting, treatment for relapsed/refractory MM (RRMM) depends on prior 1L therapy. In the United Kingdom, 2L treatment options include bortezomib monotherapy, carfilzomib–dexamethasone (Kd), and in patients previously treated with bortezomib, carfilzomib–Rd (KRd) or Rd [12, 13, 14, 15]. The only NICE‐approved triplet regimens for lenalidomide‐refractory patients at 2L are daratumumab–Vd (DaraVd; CASTOR trial) and selinexor–Vd (SelVd; BOSTON trial), the latter of which is specifically for patients who are refractory to both lenalidomide and daratumumab [16, 17, 18].

There is a substantial unmet need for novel and more effective 2L triplet regimens, especially for lenalidomide‐refractory patients. With the recent integration of DaraRd as 1L standard of care for ASCT‐ineligible patients (representing approximately two‐thirds of all newly diagnosed patients), the number of lenalidomide‐refractory patients at 2L is expected to rise. Consequently, understanding the real‐world 2L outcomes of patients who were previously exposed to, or became refractory to, lenalidomide is crucial.

The ongoing DREAMM‐7 (NCT04246047) and DREAMM‐8 (NCT04484623) trials are investigating the efficacy and safety of belantamab mafodotin, a humanised immunoglobulin G1 antibody–drug conjugate that binds specifically to B‐cell maturation antigen (BCMA) [19, 20]. In DREAMM‐7, patients with RRMM receive belantamab mafodotin in combination with Vd versus DaraVd [19]. In DREAMM‐8, patients with RRMM receive belantamab mafodotin in combination with pomalidomide–dexamethasone versus pomalidomide–Vd [20]. To contextualise the outcomes from the DREAMM‐7 and DREAMM‐8 trials, we conducted a real‐world study using data collated by the National Cancer Registration and Analysis Service (NCRAS) within the National Health Service (NHS) in England. The aim of this study was to describe the baseline characteristics, treatment patterns and clinical outcomes of patients diagnosed with RRMM and treated across multiple lines of therapy between 2013 and 2020 in England. To ensure relevance to the DREAMM‐7 and DREAMM‐8 trials, the study cohorts, namely, DREAMM‐7‐like (D7‐like) and DREAMM‐8‐like (D8‐like), were broadly aligned to the eligibility criteria outlined in these trials. In this manuscript, we specifically examine baseline characteristics and outcomes for lenalidomide‐exposed and lenalidomide‐refractory patients treated with DaraVd in the D7‐like and D8‐like cohorts. As DaraVd was the only NICE‐approved triplet regimen for lenalidomide‐refractory patients at 2L, we focus on outcomes for patients treated with DaraVd at 2L.

## 2 METHODS

### 2.1 Study design

This was a descriptive, retrospective, non‐interventional study that used data collated and maintained by the NCRAS (study ID: GSK2857916). This study identified adult patients diagnosed with MM from 1 January 2013 until 31 December 2020. Study follow‐up was from the commencement of 2L or 3L for patients that met all inclusion criteria (index line) until 31 October 2022. Patient data were processed in compliance with the legal instruction under which the NCRAS collate records for patients with cancer. Further details on the study design and data collection methods can be found in the Supporting Information.

### 2.2 Study objectives

The study's primary objective was to describe the treatment patterns of patients treated for MM during the study period. Secondary objectives included characterisation of baseline characteristics and clinical outcomes, such as time to next treatment or death (TTNTD; an alternative to progression‐free survival [PFS], which was not recorded in the NCRAS database), time to treatment discontinuation or death (TTDD) and overall survival (OS). In this manuscript, we focus on baseline characteristics and treatment outcomes (TTNTD, TTDD and OS) for patients who received DaraVd at 2L.

### 2.3 Study assessments

The baseline characteristics described for each cohort were age, gender, ethnicity, stage, Eastern Cooperative Oncology Group performance status at index date, presence of ASCT, lytic bone lesions and extramedullary plasmacytomas between diagnosis and index date. Treatment patterns were described for the index line of treatment as well as for any prior or subsequent line of treatment. TTNTD, TTDD and OS were estimated using Kaplan–Meier methodology. TTNTD was defined as the time from the start of line of treatment until the start of a new line of treatment or death due to any cause; patients lost to follow‐up or still in the same line of treatment at the end of the study period were censored. TTDD was defined as the time from the start of index line of treatment until the earliest of: death during follow‐up, last administration plus one cycle length or start of a new line minus 1 day. OS was defined as the time from initiation of the index line of treatment until all‐cause death. Patients lost to follow‐up or still alive at the end of the study period were censored.

### 2.4 Patient eligibility criteria and cohorts

This study included adult patients living in England at the date of MM diagnosis, with ≥1 incident primary diagnosis of MM within the study timeframe. Within the National Cancer Registration Dataset, date of diagnosis is the incidence date of the cancer, derived from multiple sources, with date of histological confirmation taking priority. To be included in the study, patients were required to be ≥18 years of age at the date of diagnosis. Patients were excluded from the study if diagnosed via death certificate only or if they had no recorded date of diagnosis within the prespecified study period. Patients were excluded from the study if they had received treatments funded by the Cancer Drugs Fund (CDF), in line with an embargo on reporting outcomes for CDF indications prior to a final decision by NICE.

Individuals were eligible for the D7‐like cohort if they met the following criteria: received ≥1 prior line; previous line ended >14 days before the start of line; any monoclonal antibody‐containing prior line ended >30 days before the start of line; not refractory to anti‐CD38 therapies or bortezomib; and had no prior treatment with belantamab mafodotin or other anti‐BCMA therapy. Individuals were eligible for the D8‐like cohort if they met the following criteria: received ≥1 prior line that contained ≥2 cycles of lenalidomide, were not refractory to bortezomib, and had no prior treatment with pomalidomide or belantamab mafodotin or other anti‐BCMA therapy. Inclusion in the D7‐like cohort did not require patients to have been exposed to lenalidomide, whereas inclusion in the D8‐like cohort required patients to have received ≥2 cycles of lenalidomide. Cohorts were not mutually exclusive, and it was possible for a patient to contribute to more than one, provided the selection criteria were met.

The cohort definitions of double‐class refractory patients treated at 3L+ (DR3L+) and 4L+ (DR4L+), and triple‐class refractory patients treated at 5L+ (TR5L+) are described in the Supporting Information.

### 2.5 Statistical analysis

All outcomes were assessed descriptively, and the study did not include any hypothesis testing. Therefore, all reported differences in outcomes and between groups reflect numerical and not statistical differences. Further details on statistical analysis can be found in the Supporting Information.

## 3 RESULTS

**FIGURE 1:**
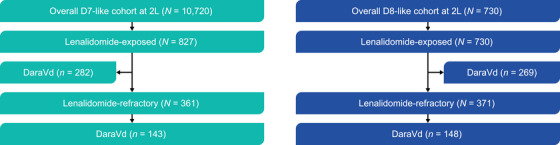
Patient flow diagram in the 2L setting. Cohorts are not mutually exclusive. Abbreviations: 2L, second‐line; D7‐like, DREAMM‐7‐like cohort; D8‐like, DREAMM‐8‐like cohort; DaraVd, daratumumab–bortezomib–dexamethasone.

The overall D7‐ and D8‐like cohorts included 10,720 and 730 patients, respectively. Among the overall cohorts, 827 (7.7%) patients in the D7‐like cohort and all patients in D8‐like cohort (*N* = 730) were lenalidomide‐exposed at 2L. The lenalidomide‐refractory population comprised 361 (3.4%) patients in the D7‐like cohort and 371 (50.8%) patients in the D8‐like cohort. Among the lenalidomide‐exposed population, 282 (34.1%) patients in the D7‐like cohort and 269 (36.8%) patients in the D8‐like cohort received DaraVd at 2L. Among the lenalidomide‐refractory patients, 143 (39.6%) patients in the D7‐like cohort and 148 (39.9%) patients in the D8‐like cohort received DaraVd at 2L (Figures 1 and S1).

Further data on baseline characteristics, treatment patterns and clinical outcomes of patients treated at 3L in the D8‐like cohort are reported in the Supporting Information.

### 3.1 Baseline characteristics

Baseline characteristics of patients with MM treated at 2L in the overall D7‐like cohort (*N *= 10,720) are reported in Table 1. Among all lenalidomide‐exposed and lenalidomide‐refractory patients in both the D7‐like and D8‐like cohorts treated at 2L, baseline characteristics for patients who received DaraVd are reported in Table 2 and baseline characteristics for patients who received other regimens are reported in Table S1. Baseline characteristics of DR3L+, DR4L+ and TR5L+ patients by regimen are reported in Table S2. Baseline characteristics of patients excluded from this study owing to the CDF embargo at the time of analysis are described in Table S3 for patients treated at 2L and Table S4 for DR3L+, DR4L+ and TR5L+ patients.

**TABLE 1:** Baseline characteristics of patients treated at 2L in the overall D7‐like cohort.

		Overall D7‐like cohort
Total patients; *N* (%)		10,720 (100.0)
Age at line start; mean (SD)	Years	71.2 (10.5)
Age at line start; *N* (%)	18–44	119 (1.1)
	45–64	2505 (23.4)
	65–74	3637 (33.9)
	75–84	3563 (33.2)
	85+	896 (8.4)
Gender; *N* (%)	Male	6225 (58.1)
	Female	4495 (41.9)
Ethnicity; *N* (%)	Asian	326 (3.0)
	Black	486 (4.5)
	White	9402 (87.7)
	Other	241 (2.2)
	Unknown	265 (2.5)
Stage^a^; *N* (%)	1	963 (9.0)
	2	1219 (11.4)
	3	1095 (10.2)
	Unstaged	7443 (69.4)
ECOG performance status; *N* (%)	0	2340 (21.8)
	1	3886 (36.2)
	2	1319 (12.3)
	3–4	250 (2.3)
	Unknown	2925 (27.3)
ASCT; *N* (%)	No	7729 (72.1)
	Yes	2991 (27.9)
Lytic bone lesion; *N* (%)	No	10,469 (97.7)
	Yes	251 (2.3)
Extramedullary plasmacytoma; *N* (%)	No	10,637 (99.2)
	Yes	83 (0.8)
Prior PI exposure; *N* (%)	No	3316 (30.9)
	Yes	7404 (69.1)
Prior IMID exposure; *N* (%)	No	5051 (47.1)
	Yes	5669 (52.9)
Prior lenalidomide exposure; *N* (%)	No	9893 (92.3)
	Yes	827 (7.7)
Lenalidomide‐refractory; *N* (%)	No	10,359 (96.6)
	Yes	361 (3.4)
Prior anti‐CD38 mAb exposure; *N* (%)	No	10,673 (99.6)
	Yes	47 (0.4)
Available follow‐up from MM diagnosis; median (IQR)	Months	47.7 (31.9–69.1)
Available follow‐up from start of line; median (IQR)	Months	18.4 (8.8–32.3)

Presence of ASCT (OPCS‐4 code: X334) sourced from National Cancer Registration Dataset, HES APC and OP datasets between date of diagnosis with MM and start of 2L. Presence of lytic bone lesions (ICD‐10 codes: M895, M899) sourced from HES APC and OP datasets between date of diagnosis with MM and start of 2L. Extramedullary plasmacytoma (ICD‐10 code: C902) sourced from National Cancer Registration Dataset, HES APC and OP datasets between date of diagnosis with MM and start of 2L.

Abbreviations: 2L, second‐line; ASCT, autologous stem cell transplant; D7‐like, DREAMM‐7‐like; ECOG, Eastern Cooperative Oncology Group; HES APC, hospital episode statistics admitted patient care; ICD‐10, International Classification of Diseases, Tenth Revision; IMID, immunomodulatory drugs; IQR, interquartile range; mAb, monoclonal antibody; MM, multiple myeloma; OP, outpatient; OPCS‐4, Office of Population Censuses and Surveys Classification of Surgical Operations and Procedures, Fourth Revision; PI, proteasome inhibitor; SD, standard deviation.

^a^ Stage at start of 2L is recorded based on the International Staging System.

**TABLE 2:** Baseline characteristics of patients treated with DaraVd at 2L in the D7‐like and D8‐like cohorts.

		D7‐like cohort		D8‐like cohort	
		Len‐exposed	Len‐refractory	Len‐exposed	Len‐refractory
Total patients; *N* (%)		282 (34.1)	143 (39.6)	269 (36.8)	148 (39.9)
Age at line start; mean (SD)	Years	75.2 (8.7)	75.6 (7.9)	75.3 (8.7)	75.7 (7.8)
Age at line start; *N* (%)	18–44	1 (0.4)	1 (0.7)	1 (0.4)	1 (0.7)
	45–64	29 (10.3)	11 (7.7)	26 (9.7)	10 (6.8)
	65–74	84 (29.8)	46 (32.2)	81 (30.1)	50 (33.8)
	75–84	138 (48.9)	72 (50.3)	132 (49.1)	72 (48.6)
	85+	30 (10.6)	13 (9.1)	29 (10.8)	15 (10.1)
Gender; *N* (%)	Male	153 (54.3)	78 (54.5)	149 (55.4)	81 (54.7)
	Female	129 (45.7)	65 (45.5)	120 (44.6)	67 (45.3)
Ethnicity; *N* (%)	Asian	8 (2.8)	4 (2.8)	8 (3.0)	4 (2.7)
	Black	15 (5.3)	9 (6.3)	15 (5.6)	9 (6.1)
	White	247 (87.6)	121 (84.6)	235 (87.4)	126 (85.1)
	Other	2 (0.7)	1 (0.7)	2 (0.7)	1 (0.7)
	Unknown	10 (3.5)	8 (5.6)	9 (3.3)	8 (5.4)
Stage^a^; *N* (%)	1	27 (9.6)	14 (9.8)	27 (10.0)	15 (10.1)
	2	26 (9.2)	15 (10.5)	26 (9.7)	15 (10.1)
	3	28 (9.9)	12 (8.4)	27 (10.0)	12 (8.1)
	Unstaged	201 (71.3)	102 (71.3)	189 (70.3)	106 (71.6)
ECOG performance status; *N* (%)	0	26 (9.2)	13 (9.1)	24 (8.9)	13 (8.8)
	1	106 (37.6)	58 (40.6)	98 (36.4)	59 (39.9)
	2	46 (16.3)	30 (21.0)	46 (17.1)	31 (20.9)
	3–4	4 (1.4)	4 (2.8)	4 (1.5)	4 (2.7)
	Unknown	100 (35.5)	38 (26.6)	97 (36.1)	41 (27.7)
ASCT; *N* (%)	No	264 (93.6)	139 (97.2)	253 (94.1)	143 (96.6)
	Yes	18 (6.4)	4 (2.8)	16 (5.9)	5 (3.4)
Lytic bone lesion; *N* (%)	No	277 (98.2)	141 (98.6)	264 (98.1)	146 (98.6)
	Yes	5 (1.8)	2 (1.4)	5 (1.9)	2 (1.4)
Extramedullary plasmacytoma; *N* (%)	No	280 (99.3)	142 (99.3)	267 (99.3)	147 (99.3)
	Yes	2 (0.7)	1 (0.7)	2 (0.7)	1 (0.7)
Prior PI exposure; *N* (%)	No	274 (97.2)	142 (99.3)	266 (98.9)	147 (99.3)
	Yes	8 (2.8)	1 (0.7)	3 (1.1)	1 (0.7)
Prior IMID exposure; *N* (%)	No	–	–	–	–
	Yes	282 (100.0)	143 (100.0)	269 (100.0)	148 (100.0)
Prior lenalidomide exposure; *N* (%)	No	–	–	–	–
	Yes	282 (100.0)	143 (100.0)	269 (100.0)	148 (100.0)
Lenalidomide‐refractory; *N* (%)	No	139 (49.3)	–	121 (45.0)	–
	Yes	143 (50.7)	143 (100.0)	148 (55.0)	148 (100.0)
Prior anti‐CD38 mAb exposure; *N* (%)	No	282 (100.0)	143 (100.0)	269 (100.0)	148 (100.0)
	Yes	–	–	–	–
Available follow‐up from MM diagnosis; median (IQR)	Months	30.6 (24.1–41.0)	28.9 (22.6–37.0)	30.5 (24.0–39.6)	28.8 (22.6–36.9)
Available follow‐up from start of line; median (IQR)	Months	12.0 (4.5–19.2)	10.8 (3.8–17.6)	11.7 (4.5–18.9)	10.5 (3.8–17.4)

Presence of ASCT (OPCS‐4 code: X334) sourced from National Cancer Registration Dataset, HES APC and OP datasets between date of diagnosis with MM and start of 2L. Presence of lytic bone lesions (ICD‐10 codes: M895, M899) sourced from HES APC and OP datasets between date of diagnosis with MM and start of 2L. Extramedullary plasmacytoma (ICD‐10 code: C902) sourced from National Cancer Registration Dataset, HES APC and OP datasets between date of diagnosis with MM and start of 2L.

Abbreviations: 2L, second‐line; ASCT, autologous stem cell transplant; D7‐like, DREAMM‐7‐like; D8‐like, DREAMM‐8‐like; DaraVd, daratumumab–bortezomib–dexamethasone; ECOG, Eastern Cooperative Oncology Group; HES APC, hospital episode statistics admitted patient care; ICD‐10, International Classification of Diseases, Tenth Revision; IMID, immunomodulatory drugs; IQR, interquartile range; Len, lenalidomide; mAb, monoclonal antibody; MM, multiple myeloma; OP, outpatient; OPCS‐4, Office of Population Censuses and Surveys Classification of Surgical Operations and Procedures, Fourth Revision; PI, proteasome inhibitor; SD, standard deviation.

^a^ Stage at start of 2L is recorded based on the International Staging System.

### 3.2 Treatment patterns

In the overall D7‐like cohort, patients received Rd (25.4%), DaraVd (19.9%), Vd (8.5%), KRd (1.6%) and Kd (1.6%) at 2L. In the D7‐like cohort, the proportion who received DaraVd at 2L was higher amongst lenalidomide‐exposed (34.1%) and lenalidomide‐refractory patients (39.6%). Overall, the modal interval between bortezomib administrations was 7 days. For lenalidomide‐exposed/refractory patients in both the D7‐like and D8‐like cohorts, Table S5 presents an overview of 1L treatments and drug classes by regimens received at 2L. For lenalidomide‐exposed/refractory patients treated at 2L in the D7‐like and D8‐like cohorts, Tables S6 and S7 report subsequent regimens.

### 3.3 TTNTD

**FIGURE 2:**
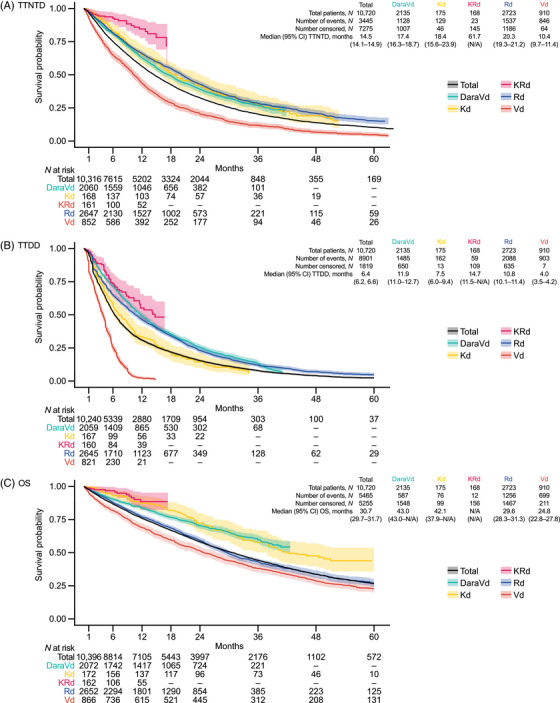
Clinical outcomes of patients treated at 2L in the overall D7‐like cohort in total and for patients treated with NICE‐approved regimens. TTNTD, TTDD and OS outcomes of patients treated with non‐NICE‐approved regimens at 2L in the overall D7‐like cohort can be found in Table S8. Abbreviations: 2L, second‐line; CI, confidence interval; D7‐like, DREAMM‐7‐like; DaraVd, daratumumab–bortezomib–dexamethasone; Kd, carfilzomib–dexamethasone; KRd, carfilzomib–lenalidomide–dexamethasone; MM, multiple myeloma; N/A, not available; NICE, National Institute for Health and Care Excellence; OS, overall survival; Rd, lenalidomide–dexamethasone; TTDD, time to treatment discontinuation or death; TTNTD, time to next treatment or death; Vd, bortezomib–dexamethasone.

In the overall D7‐like cohort, the median (95% confidence interval [CI]) TTNTD for patients treated at 2L was 14.5 (14.1–14.9) months (Figure 2). In the D7‐like cohort, the median (95% CI) TTNTD for lenalidomide‐exposed and lenalidomide‐refractory patients treated at 2L was 11.7 (10.3–13.2) and 8.5 (6.9–10.3) months, respectively (Figure S2). In the D8‐like cohort, the median (95% CI) TTNTD for lenalidomide‐exposed and lenalidomide‐refractory patients treated at 2L was 11.2 (10.1–12.9) months and 8.1 (6.7–10.1) months, respectively (Figure S2).

**FIGURE 3:**
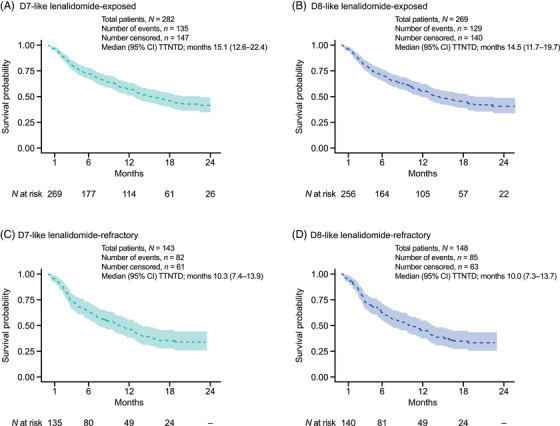
TTNTD from initiation of DaraVd at 2L. TTNTD is measured in months from start of line of therapy. TTNTD event is the earliest of a new line start or death during follow‐up. Patients remaining on the line of therapy or yet to start a new line and alive at follow‐up end were censored. Kaplan–Meier analysis is truncated at the point when <10 patients remain in the risk‐set. Abbreviations: 2L, second‐line; CI, confidence interval; D7‐like, DREAMM‐7‐like; D8‐like, DREAMM‐8‐like; DaraVd, daratumumab–bortezomib–dexamethasone; TTNTD, time to next treatment or death.

In the overall D7‐like cohort, the median (95% CI) TTNTD for patients treated with DaraVd at 2L (*N *= 2135) was 17.4 (16.3–18.7) months. In the D7‐like cohort, the median (95% CI) TTNTD for lenalidomide‐exposed and lenalidomide‐refractory patients who received DaraVd at 2L was 15.1 (12.6–22.4) and 10.3 (7.4–13.9) months, respectively (Figure 3). In the D8‐like cohort, the median (95% CI) TTNTD for lenalidomide‐exposed and lenalidomide‐refractory patients who received DaraVd at 2L was 14.5 (11.7–19.7) and 10.0 (7.3–13.7) months, respectively (Figure 3). A breakdown of TTNTD outcomes at 2L by different regimens in both cohorts is reported in Figure S3.

### 3.4 TTDD

In the overall D7‐like cohort, the median (95% CI) TTDD for patients treated at 2L was 6.4 (6.2–6.6) months (Figure 2). In the D7‐like cohort, the median (95% CI) TTDD for lenalidomide‐exposed and lenalidomide‐refractory patients treated at 2L was 5.5 (5.0–6.4) and 5.0 (4.3–5.6) months, respectively (Figure S4). In the D8‐like cohort, the median (95% CI) TTDD for lenalidomide‐exposed and lenalidomide‐refractory patients treated at 2L was 5.6 (5.0–6.6) and 5.0 (4.3–5.6) months, respectively (Figure S4).

**FIGURE 4:**
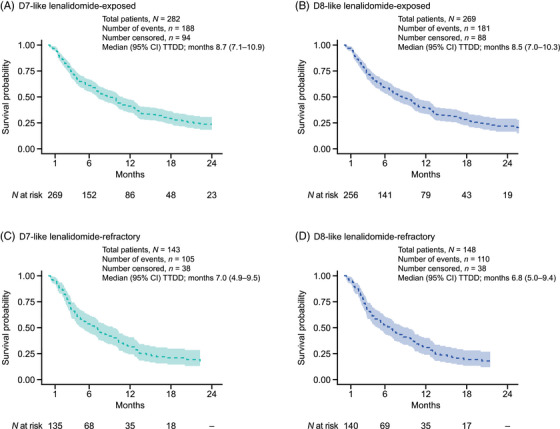
TTDD from initiation of DaraVd at 2L. TTDD is measured in months from start of line of therapy. TTDD event is the earliest of last administration plus one cycle length; start of a new line minus 1 day; death during follow‐up. Patients alive at follow‐up end with no subsequent line and last administration date of the line within a cycle length of administrative follow‐up end were censored. Kaplan–Meier analysis is truncated at the point when <10 patients remain in the risk‐set. Abbreviations: 2L, second‐line; CI, confidence interval; D7‐like, DREAMM‐7‐like; D8‐like, DREAMM‐8‐like; DaraVd, daratumumab–bortezomib–dexamethasone; TTDD, time to treatment discontinuation or death.

In the overall D7‐like cohort, the median (95% CI) TTDD for patients treated with DaraVd at 2L was 11.9 (11.0–12.7) months. In the D7‐like cohort, the median (95% CI) TTDD for lenalidomide‐exposed and lenalidomide‐refractory patients who received DaraVd at 2L was 8.7 (7.1–10.9) and 7.0 (4.9–9.5) months, respectively (Figure 4). In the D8‐like cohort, the median (95% CI) TTDD for lenalidomide‐exposed and lenalidomide‐refractory patients who received DaraVd at 2L was 8.5 (7.0–10.3) and 6.8 (5.0–9.4) months, respectively (Figure 4). A breakdown of TTDD outcomes at 2L by different regimens in both cohorts is reported in Figure S5.

### 3.5 OS

In the overall D7‐like cohort, the median (95% CI) OS for patients treated at 2L was 30.7 (29.7–31.7) months (Figure 2). In the D7‐like cohort, the median (95% CI) OS for lenalidomide‐exposed and lenalidomide‐refractory patients treated at 2L was 23.0 (20.4–26.0) and 18.5 (15.2–22.1) months, respectively (Figure S6). In the D8‐like cohort, the median (95% CI) OS for lenalidomide‐exposed and lenalidomide‐refractory patients treated at 2L was 23.0 (20.0–26.6) and 18.0 (15.1–21.6) months, respectively (Figure S6).

**FIGURE 5:**
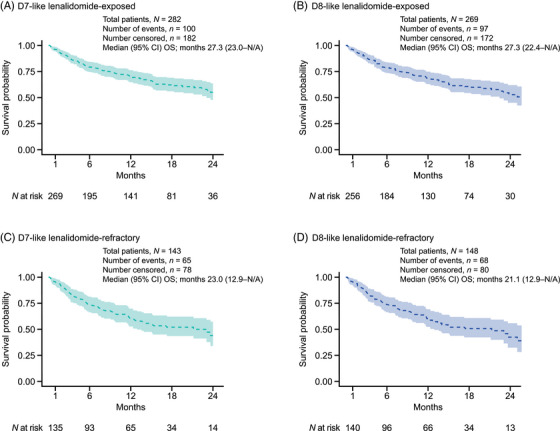
OS from initiation of DaraVd at 2L. OS is measured in months from start of line of therapy. OS event is death during follow‐up. Patients alive at follow‐up were censored. Kaplan–Meier analysis is truncated at the point when <10 patients remain in the risk‐set. Abbreviations: 2L, second‐line; CI, confidence interval; D7‐like, DREAMM‐7‐like; D8‐like, DREAMM‐8‐like; DaraVd, daratumumab–bortezomib–dexamethasone; N/A, not available; OS, overall survival.

In the D7‐like cohort, the median (95% CI) OS for patients treated with DaraVd at 2L was 43 (43.0–not available [N/A]) months. In the D7‐like cohort, the median (95% CI) OS for lenalidomide‐exposed and lenalidomide‐refractory patients who received DaraVd at 2L was 27.3 (23.0–N/A) and 23.0 (12.9–N/A) months, respectively (Figure 5). In the D8‐like cohort, the median (95% CI) OS for lenalidomide‐exposed and lenalidomide‐refractory patients who received DaraVd at 2L was 27.3 (22.4–N/A) and 21.1 (12.9–N/A) months, respectively (Figure 5). A breakdown of OS outcomes at 2L by different regimens in both cohorts is reported in Figure S7.

For the overall D7‐like cohort, TTNTD, TTDD and OS results for patients treated with NICE‐ and non‐NICE‐approved regimens at 2L are presented in Figure 2 and Table S8, respectively. TTNTD, TTDD and OS results by different regimens for DR3L+, DR4L+ and TR5L+ patients are reported in Figures S8–S10.

## 4 DISCUSSION

To our knowledge, this is the largest real‐world study of baseline characteristics, treatment patterns and clinical outcomes of patients with MM in England; we present descriptive results (without statistical testing) based on data collated by NCRAS. In our study, Rd was the most prevalent choice of treatment at 2L in the overall D7‐like cohort. However, in current clinical practice and owing to recent NICE approvals, lenalidomide is now commonly used to treat patients with MM at 1L [6, 11]. In the overall D7‐like cohort of the present study, Rd‐based regimens at 2L had the longest median TTNTD and TTDD. Across the lenalidomide‐exposed and lenalidomide‐refractory D7‐like and D8‐like cohorts, the most prevalent therapeutic regimen at 2L was DaraVd. In both D7‐like and D8‐like cohorts, treatment with DaraVd at 2L showed numerically longer TTNTD, TTDD and OS in lenalidomide‐exposed patients compared with lenalidomide‐refractory patients; however, CIs overlapped.

The phase 3 CASTOR trial (*N *= 498) demonstrated significant clinical benefits in patients with RRMM treated with DaraVd versus those treated with Vd [21]. In the CASTOR trial, patients treated with DaraVd who received one prior line of therapy had a median PFS of 27.0 months and those who received one prior line of therapy with lenalidomide had a median PFS of 21.2 months [21]. Compared with the CASTOR trial, lenalidomide‐exposed patients in the D7‐like and D8‐like cohorts who were treated with DaraVd at 2L had a numerically shorter median TTNTD of 15.1 and 14.5 months, respectively. However, this may be attributed to the small number of lenalidomide‐exposed patients in the CASTOR study (*n *= 15). Despite the small number of lenalidomide‐refractory patients at 2L in the CASTOR trial (6/122 patients at 2L; 5%), our 2L data showed a numerically similar median TTNTD (D7‐like, 10.3 months; D8‐like, 10.0 months) to the median PFS of the lenalidomide‐refractory multiline population of CASTOR (7.8 months) [21]. In the phase 3 DREAMM‐7 trial (*N* = 494), patients with RRMM and ≥1 prior line of therapy demonstrated an improved median PFS when treated with belantamab mafodotin–Vd compared with DaraVd (36.6 vs. 13.4 months; *p* < 0.00001) [22]. Median TTNTD for lenalidomide‐refractory patients in the D7‐like and D8‐like cohorts treated with DaraVd at 2L in the present study (10.3 and 10.0 months, respectively) was numerically similar to the median PFS of the lenalidomide‐refractory subgroup treated with DaraVd in the DREAMM‐7 study (8.6 months; 95% CI, 6.4–13.5) [22]. Following the reimbursement of DaraVd resulting from the CASTOR trial, a real‐world, multicentre, retrospective UK study aimed to assess the clinical outcomes of 296 patients with MM treated with DaraVd at first relapse [23]. The real‐world study showed a median PFS of 23 and 18 months for lenalidomide‐exposed patients (*n* = 44) and lenalidomide‐refractory patients (*n* = 24), respectively [23]. In comparison, our results showed numerically shorter median TTNTD for lenalidomide‐exposed (D7‐like, 15.1 months; D8‐like, 14.5 months) and lenalidomide‐refractory (D7‐like, 10.3 months; D8‐like, 10.0 months) patients treated with DaraVd at 2L [23]. However, the real‐world study was limited by small numbers of patients [23]. Overall, our data corroborate the poor clinical outcomes of lenalidomide‐refractory patients treated with DaraVd for RRMM observed in the CASTOR and DREAMM‐7 trials. With the recent integration of DaraRd at 1L, patients with MM are now more frequently exposed to and more likely to develop resistance to anti‐CD38 monoclonal antibodies during the initial stages of treatment [11]. This emphasises the need to provide patients with alternative 2L options to avoid retreatment with anti‐CD38 monoclonal antibodies at relapse.

Limitations of this study, consistent with retrospective studies utilising administrative health data, include heterogeneity of practices among treatment centres and lack of key data items. Notably, our dataset lacked information on patient progression/refractory status and treatment duration. Consequently, our study relied on algorithms that may not accurately capture these parameters. Additionally, our dataset lacked information on patient cytogenetic and performance status, and disease stage was not available for most patients. Furthermore, the number of patients with lytic bone lesions may be underreported, owing to a reliance on administrative healthcare data from the inpatient care setting, likely representing patients with more severe illness requiring hospital admission. In addition, our dataset lacked M‐protein level measurements, causing our definition of refractoriness to deviate from IMWG criteria. Thus, some patients identified as lenalidomide‐refractory may not truly be refractory, and vice versa. Another limitation pertains to the patient eligibility criteria; as the D7‐like and D8‐like cohort inclusion criteria were not identical to the inclusion criteria of DREAMM‐7 and DREAMM‐8, the observed outcomes of our study may not be directly comparable to the findings of DREAMM‐7 and DREAMM‐8. When interpreting these data, it is important to be cautious about drawing indirect comparisons to results from intention‐to‐treat populations of clinical trials. Furthermore, TTNTD may not be directly compared with PFS. Although TTNTD is widely used as an alternative to PFS in studies on MM [24, 25, 26], it may overestimate the true PFS, as events such as toxicity resolution and the washout periods can delay the next treatment. However, one strength of this study is reporting TTNTD and TTDD together, as this can provide insights regarding the impact of the time off treatment between successive lines of therapy. Furthermore, the relatively short follow‐up period of our study limits interpretation of its findings, particularly for OS outcomes. Other important limitations relate to the differences between historic and current treatment approaches due to the study timeframe. In the United Kingdom, the main 2L treatments for lenalidomide‐exposed/refractory patients include DaraVd, Kd and SelVd. The present study did not include many patients treated with Kd (*n* = 175 [1.6%] in overall D7‐like cohort) at 2L, owing to receiving NICE approval in 2020 (ENDEAVOR; NCT01568866). Similarly, our study did not include any patients treated with SelVd (BOSTON; NCT03110562), owing to its recent NICE approval on 15 May 2024. Therefore, the study dataset cannot be compared with clinical outcomes from ENDEAVOR or BOSTON. In addition, owing to the study timeframe, the D7‐like cohort comprised fewer lenalidomide‐exposed (7.7%) and lenalidomide‐refractory patients (3.4%) than would be expected in current clinical practice, based on increasing 1L lenalidomide use due to recent NICE approvals [6, 11]. Finally, the NCRAS dataset lacks information on CD38 sensitivity; yet, given the recent incorporation of daratumumab at 1L, prior CD38 monoclonal antibody exposure and refractoriness are expected to be frequently observed at 2L.

## 5 CONCLUSION

Following the approval of new effective treatments for RRMM, patient outcomes continue to improve [3, 27]. However, patients still relapse, and the disease remains incurable. In the United Kingdom, 1L treatments now involve four drug combinations or DaraRd, depending on fitness [8, 11]. The choice of 2L treatment depends on frontline regimen, tolerability and refractoriness to prior therapies. With the incorporation of lenalidomide as continuous or maintenance therapy at 1L, and the increasing use of DaraRd at 1L, the number of patients who will become lenalidomide‐ and daratumumab‐refractory at first relapse is expected to increase. Our results for DaraVd suggest that outcomes may be poor for lenalidomide‐refractory patients at 2L in England. These findings, along with the poor clinical outcomes for lenalidomide‐refractory patients observed in the CASTOR, DREAMM‐7, ENDEAVOR and BOSTON studies, highlight the need for new effective combinations with manageable safety profiles in this setting. BCMA‐directed treatments have demonstrated efficacy in heavily pretreated patients and may present a suitable therapeutic option at earlier lines of treatment [28]. Our study serves as an important historical reference for patients treated at 2L in England and provides valuable context for the results of key clinical trials such as CASTOR, DREAMM‐7 and DREAMM‐8.

## AUTHOR CONTRIBUTIONS

Christina‐Jane Crossman‐Barnes, Laura Cornic, Philip Woods and Zeyad Khalaf contributed to the study conceptualisation. Christina‐Jane Crossman‐Barnes, Laura Cornic, Philip Woods, Sophie Jose and Zeyad Khalaf developed the study methodology. Sophie Jose was responsible for software development, formal analysis, investigation and resource provision. All the authors were involved in writing, reviewing and editing the manuscript. Data visualisation was handled by Christina‐Jane Crossman‐Barnes, Laura Cornic, Megan Soutar and Philip Woods. Supervision was provided by Christina‐Jane Crossman‐Barnes and Philip Woods. Project administration was provided by Christina‐Jane Crossman‐Barnes and Megan Soutar. Laura Cornic acquired the project funding.

## CONFLICT OF INTEREST STATEMENT

Laura Cornic, Christina‐Jane Crossman‐Barnes, Zeyad Khalaf, Megan Soutar and Philip Woods are employed by GSK and hold financial equities in GSK. Sally Moore, Kwee Yong and Sophie Jose have no conflicts of interest to disclose.

## ETHICS STATEMENT

The authors have confirmed ethical approval statement is not needed for this submission.

## PATIENT CONSENT STATEMENT

The authors have confirmed patient consent statement is not needed for this submission.

## CLINICAL TRIAL REGISTRATION

The authors have confirmed clinical trial registration is not needed for this submission.

## Supporting information



Supporting Information

## Data Availability

Data used for this publication was collated by the National Cancer Registration and Analysis Service (NCRAS) of the National Health Service in England. For access to anonymised subject level data, please contact NCRAS.

## References

[1] Dimopoulos MA , Moreau P , Terpos E , Mateos MV , Zweegman S , Cook G , et al. Multiple myeloma: EHA‐ESMO Clinical Practice Guidelines for diagnosis, treatment and follow‐up(dagger). Ann Oncol. 2021;32(3):309–322. 33549387 10.1016/j.annonc.2020.11.014

[2] Cancer Research UK . Myeloma statistics. Available from: https://www.cancerresearchuk.org/health‐professional/cancer‐statistics/statistics‐by‐cancer‐type/myeloma/survival2018#:~:text=There%20are%20around%206%2C000%20new,year%20(2016%2D2018). Accessed 19 Feb 2024.

[3] Su CT , Ye JC . Emerging therapies for relapsed/refractory multiple myeloma: CAR‐T and beyond. J Hematol Oncol. 2021;14(1):115. 34301270 10.1186/s13045-021-01109-y PMC8299593

[4] Yong K , Delforge M , Driessen C , Fink L , Flinois A , Gonzalez‐McQuire S , et al. Multiple myeloma: patient outcomes in real‐world practice. Br J Haematol. 2016;175(2):252–264. 27411022 10.1111/bjh.14213 PMC5096152

[5] National Institute of Health and Care Excellence . Myeloma: diagnosis and management. 2018. Available from: www.nice.org.uk/guidance/ng35. Accessed 7 Mar 2024.

[6] National Institute of Health and Care Excellence . Lenalidomide maintenance treatment after an autologous stem cell transplant for newly diagnosed multiple myeloma [TA680]. 2021. Available from: www.nice.org.uk/guidance/ta680. Accessed 7 Mar 2024. 40737469

[7] National Institute for Health and Care Excellence . Bortezomib for induction therapy in multiple myeloma before high‐dose chemotherapy and autologous stem cell transplantation [TA311]. 2014. Available from: www.nice.org.uk/guidance/ta311. Accessed 8 Apr 2024.

[8] National Institute for Health and Care Excellence. Daratumumab in combination for untreated multiple myeloma when a stem cell transplant is suitable [TA763]. 2022. Available from: www.nice.org.uk/guidance/ta763. Accessed 8 Apr 2024. 40455893

[9] National Institute for Health and Care Excellence. Bortezomib and thalidomide for the first‐line treatment of multiple myeloma [TA228]. 2011. Available from: https://www.nice.org.uk/guidance/ta228. Accessed 8 Apr 2024.

[10] National Institute for Health and Care Excellence . Lenalidomide plus dexamethasone for previously untreated multiple myeloma [TA587]. 2019. Available from: www.nice.org.uk/guidance/ta587. Accessed 7 Mar 2024. 40906874

[11] National Institute of Health and Care Excellence . Daratumumab with lenalidomide and dexamethasone for untreated multiple myeloma when a stem cell transplant is unsuitable [TA917]. 2023. Available from: www.nice.org.uk/guidance/ta917. Accessed 7 Mar 2024. 40198020

[12] National Institute for Health and Care Excellence . Bortezomib monotherapy for relapsed multiple myeloma [TA129]. 2007. Available from: www.nice.org.uk/guidance/ta129. Accessed 7 Mar 2024.

[13] National Institute for Health and Care Excellence . Carfilzomib with dexamethasone and lenalidomide for previously treated multiple myeloma [TA695]. 2021. Available from: www.nice.org.uk/guidance/ta695. Accessed 7 Mar 2024. 40743379

[14] National Institute for Health and Care Excellence . Carfilzomib for previously treated multiple myeloma [TA657]. 2020. Available from: www.nice.org.uk/guidance/ta657. Accessed 7 Mar 2024. 40875872

[15] National Institute of Health and Care Excellence . Lenalidomide plus dexamethasone for multiple myeloma after 1 treatment with bortezomib [TA586]. 2019. Available from: www.nice.org.uk/guidance/ta586. Accessed 7 Mar 2024. 40906873

[16] Grosicki S , Simonova M , Spicka I , Pour L , Kriachok I , Gavriatopoulou M , et al. Once‐per‐week selinexor, bortezomib, and dexamethasone versus twice‐per‐week bortezomib and dexamethasone in patients with multiple myeloma (BOSTON): a randomised, open‐label, phase 3 trial. Lancet. 2020;396(10262):1563–1573. 33189178 10.1016/S0140-6736(20)32292-3

[17] National Institute for Health and Care Excellence . Selinexor with bortezomib and dexamethasone for previously treated multiple myeloma [TA974]. 2024. Available from: www.nice.org.uk/guidance/ta974. Accessed 16 May 2024. 40073189

[18] National Institute of Health and Care Excellence . Daratumumab with bortezomib and dexamethasone for previously treated multiple myeloma [TA897]. 2019. Available from: www.nice.org.uk/guidance/ta897. Accessed 7 Mar 2024. 40146862

[19] Hungria V , Robak P , Hus M , Zherebtsova V , Ward C , Ho PJ , et al. Belantamab mafodotin, bortezomib, and dexamethasone for multiple myeloma. N Engl J Med. 2024;391(5):393‐407. 38828933 10.1056/NEJMoa2405090

[20] Dimopoulos MA , Beksac M , Pour L , Delimpasi S , Vorobyev V , Quach H , et al. Belantamab mafodotin, pomalidomide, and dexamethasone in multiple myeloma. N Engl J Med. 2024;391(5):408‐421. 38828951 10.1056/NEJMoa2403407

[21] Mateos MV , Sonneveld P , Hungria V , Nooka AK , Estell JA , Barreto W , et al. Daratumumab, bortezomib, and dexamethasone versus bortezomib and dexamethasone in patients with previously treated multiple myeloma: three‐year follow‐up of CASTOR. Clin Lymphoma Myeloma Leuk. 2020;20(8):509–518. 32482541 10.1016/j.clml.2019.09.623

[22] Maria‐Victoria Mateos PR , Hus M , Fu C , Zherebtsova V , Ward C , Joy Ho P , et al. DREAMM‐7 update: subgroup analyses from a phase 3 trial of belantamab mafodotin (belamaf) + bortezomib and dexamethasone (BVd) vs daratumumab, bortezomib, and dexamethasone (DVd) in relapsed/refractory multiple myeloma (RRMM). 2024. Available from: https://ascopubs.org/doi/10.1200/JCO.2024.42.16_suppl.7503. Accessed 7 Mar 2024.

[23] McMillan A , Basu S , Karunanithi K , Parkins E , Lau EYM , Cook G , et al. Daratumumab, bortezomib and dexamethasone at first relapse for patients with multiple myeloma: a real‐world multicentre UK retrospective analysis. Br J Haematol. 2023;201(4):682–689. 36822820 10.1111/bjh.18703

[24] Hari P , Ung B , Abouzaid S , Agarwal A , Parikh K . Lenalidomide maintenance post‐transplantation in newly diagnosed multiple myeloma: real‐world outcomes and costs. Future Oncol. 2019;15(35):4045–4056. 31625415 10.2217/fon-2019-0422

[25] Abe Y , Ikeda S , Kitadate A , Narita K , Kobayashi H , Miura D , et al. Low hexokinase‐2 expression‐associated false‐negative (18)F‐FDG PET/CT as a potential prognostic predictor in patients with multiple myeloma. Eur J Nucl Med Mol Imaging. 2019;46(6):1345–1350. 30903198 10.1007/s00259-019-04312-9

[26] Chari A , Parikh K , Ni Q , Abouzaid S . Treatment patterns and clinical and economic outcomes in patients with newly diagnosed multiple myeloma treated with lenalidomide‐ and/or bortezomib‐containing regimens without stem cell transplant in a real‐world setting. Clin Lymphoma Myeloma Leuk. 2019;19(10):645–655. 31377207 10.1016/j.clml.2019.06.007

[27] Puertas B , Gonzalez‐Calle V , Sobejano‐Fuertes E , Escalante F , Queizan JA , Barez A , et al. Novel agents as main drivers for continued improvement in survival in multiple myeloma. Cancers (Basel). 2023;15(5):1558. 36900349 10.3390/cancers15051558 PMC10000382

[28] Lonial S , Lee HC , Badros A , Trudel S , Nooka AK , Chari A , et al. Belantamab mafodotin for relapsed or refractory multiple myeloma (DREAMM‐2): a two‐arm, randomised, open‐label, phase 2 study. Lancet Oncol. 2020;21(2):207–221. 31859245 10.1016/S1470-2045(19)30788-0

