# Supplementary material for: Real‐world characteristics and outcomes of patients with multiple myeloma receiving second‐line treatment in England
Source: EJHaem. 2024 Dec 5;6(1):e1058. doi: 10.1002/jha2.1058 (PMC11756965; doi:10.1002/jha2.1058)
Supplement: Supplementary file 1 — Supporting Information [file JHA2-6-e1058-s001.docx]

# DATA SUPPLEMENT

## SUPPLEMENTARY METHODS

### Study design

Lines of therapy were determined by treatment changes between consecutive systemic therapy regimens. Lines of therapy changed when all drugs were changed, when there was any alteration in the composition of drug class(es), when there was any change in the composition of drugs delivered in which the drug class(es) was unchanged, and when there was any alteration in composition of drugs delivered in which the treatment-free interval between the two regimens totalled >60 days for autologous stem cell transplantation (ASCT)-negative and >100 days for ASCT-positive patients. The ‘treatment-free interval’ is defined as the period in days between the end of a regimen and the start of a subsequent regimen, with a regimen spanning the interval between the earliest known cycle start date and the last known administration date for the regimen. When a regimen contains only a single cycle and/or administration start date, such that the regimen duration is equal to 0 days, the duration is imputed to one that is equal to the ‘usual’ cycle duration for the regimen in question. In agreement with International Myeloma Working Group (IMWG) criteria, patients were classed as refractory to lenalidomide when a new line was started within 60 days of completion of the last line of therapy containing lenalidomide.^29^

### Data collection methods

The National Cancer Registration and Analysis Service (NCRAS) patient data were collated from a range of sources, including the National Cancer Registration Dataset, the Hospital Episode Statistics datasets and the Systemic Anti-Cancer Therapy dataset.^30-32^ Patients were censored in the event of death, relocation outside of England or at the end of the study period.

### Patient eligibility criteria and cohorts

As well as the DREAMM-7-like (D7-like) and DREAMM-8-like (D8-like) cohorts, this study additionally included cohorts of patients diagnosed with MM from 1 January 2013 until 31 December 2019, and who were double-class refractory and treated at 3L or higher (DR3L+), 4L or higher (DR4L+), and triple-class refractory treated at 5L or higher (TR5L+). These cohorts of patients were not stratified based on D7-like and D8-like eligibility criteria. Individuals were eligible for the DR3L+ and DR4L+ cohorts if they had prior exposure to a proteasome inhibitor (PI) and an immunomodulatory drug (IMID) with a change of line within 60 days of its completion, at the start of 3L or higher, and 4L or higher, respectively. Individuals were eligible for the TR5L+ cohort if they had prior exposure to a PI, an IMID and an anti-CD38 monoclonal antibody with a change of line within 60 days of its completion, at the start of 5L or higher.

### Statistical analysis

Continuous variables were summarised by means (standard deviation [SD]), medians, quartiles and ranges. Frequencies and percentages were presented for categorical variables. Patients were described in terms of their baseline demographic and clinical characteristics; treatment pathways were characterised from date of diagnosis of multiple myeloma (MM) to end of follow-up.

For survival analyses, Kaplan–Meier estimates (and their corresponding two-sided 95% confidence intervals [CIs]) were reported for patients at 1, 6, 12, 18, 24 and 36 months following the index date, subject to a minimum of 10 at-risk participants at each interval. Estimates were presented alongside associated summary statistics, including median survival time.

## SUPPLEMENTARY RESULTS

This analysis includes D8-like cohort results at third-line (3L) within the data supplement. However, not all corresponding figures and tables were generated for the D7-like cohort.

### Baseline characteristics and treatments at 3L

Baseline characteristics and treatments of lenalidomide-exposed and lenalidomide-refractory patients treated at 3L in the D8-like cohort are reported in **Table S9**. For lenalidomide-exposed and lenalidomide-refractory patients treated at 3L in the D8-like cohort, a summary of prior treatments by regimen and drug classes can be found in **Table S10**, and summary of subsequent lines of therapy received are reported in **Table S7**. Subsequent regimens and drug classes for patients in the D8-like cohort meeting eligibility criteria at second-line (2L) or 3L are reported in **Table S11**.

### TTNTD, TTDD and OS at 3L

For the D8-like cohort, the overall time to next treatment or death (TTNTD) and TTNTD by regimen at 3L are reported in **Figures S11–S12**. For the D8-like cohort, the overall time to treatment discontinuation or death (TTDD) and TTDD by regimen at 3L are reported in **Figures S13–S14**. For the D8-like cohort, overall survival (OS) and OS by regimen at 3L are reported in **Figures S15–S16**.

FIGURE S1. Study attrition diagram


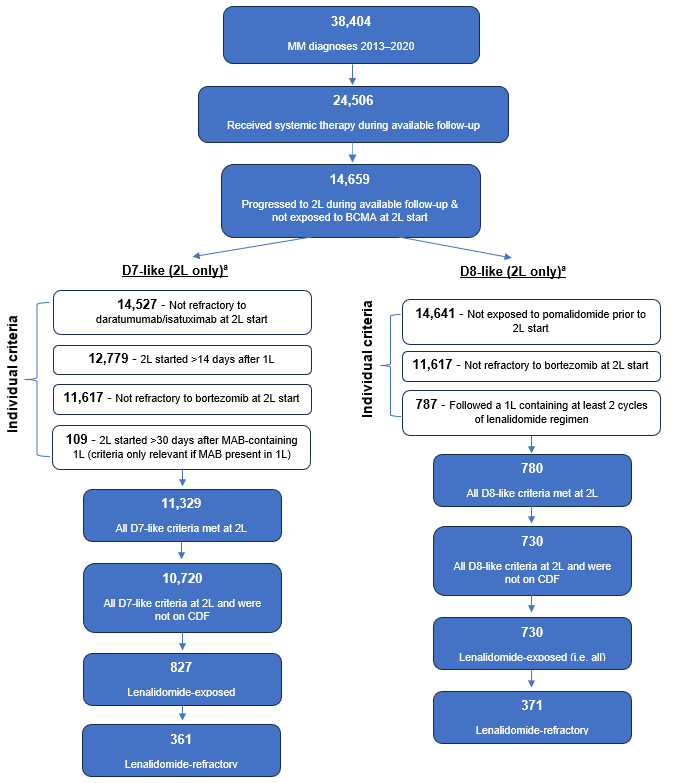


Abbreviations: 1L, first-line; 2L, second-line; BCMA, B-cell maturation antigen; CDF, Cancer Drugs Fund; D7, DREAMM-7; D8, DREAMM-8; MAB, monoclonal antibody; MM, multiple myeloma.

^a^Final cohorts are not mutually exclusive.

FIGURE S2. TTNTD from initiation of 2L

1. D7-like lenalidomide-exposed


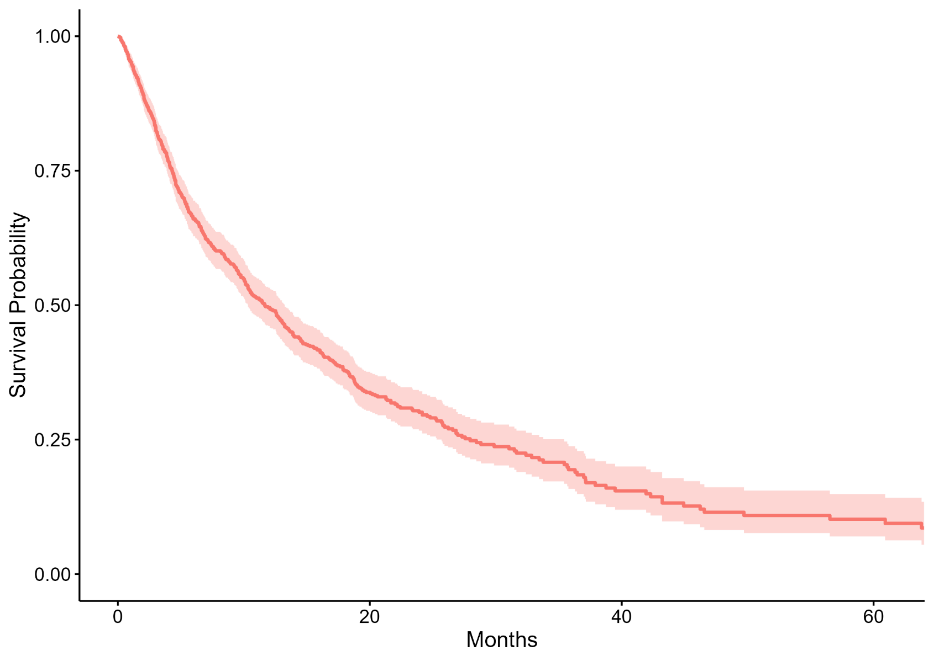


*N* = 827

Number of events: 554

Numbers censored: 273

Median (95% CI) TTNTD; months: 11.7 (10.3, 13.2)

| Months | 1 | 6 | 12 | 18 | 24 | 36 | 48 | 60 |
| --- | --- | --- | --- | --- | --- | --- | --- | --- |
| *N* at risk | 782 | 506 | 338 | 204 | 115 | 41 | 20 | 13 |

1. D7-like lenalidomide-refractory


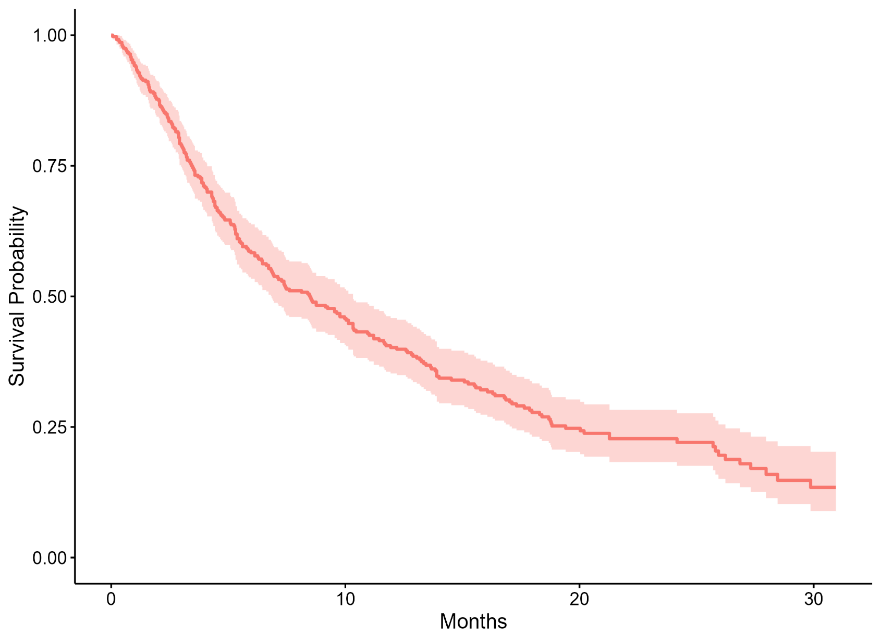


*N* = 361

Number of events: 264

Numbers censored: 97

Median (95% CI) TTNTD; months: 8.5 (6.9, 10.3)

| Months | 1 | 6 | 12 | 18 | 24 |
| --- | --- | --- | --- | --- | --- |
| *N* at risk | 339 | 195 | 121 | 67 | 32 |

1. D8-like lenalidomide-exposed


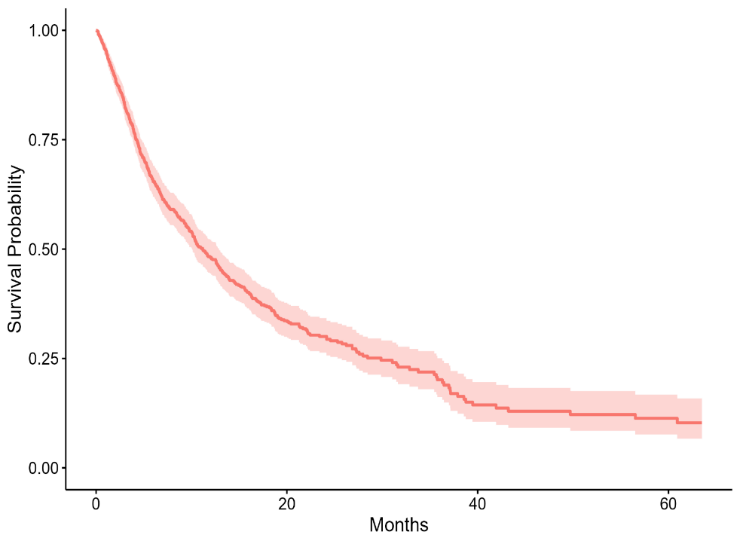


*N* = 730

Number of events: 477

Numbers censored: 253

Median (95% CI) TTNTD; months: 11.2 (10.1, 12.9)

| Months | 1 | 6 | 12 | 18 | 24 | 36 | 48 | 60 |
| --- | --- | --- | --- | --- | --- | --- | --- | --- |
| *N* at risk | 691 | 438 | 282 | 167 | 92 | 33 | 18 | 11 |

1. D8-like lenalidomide-refractory


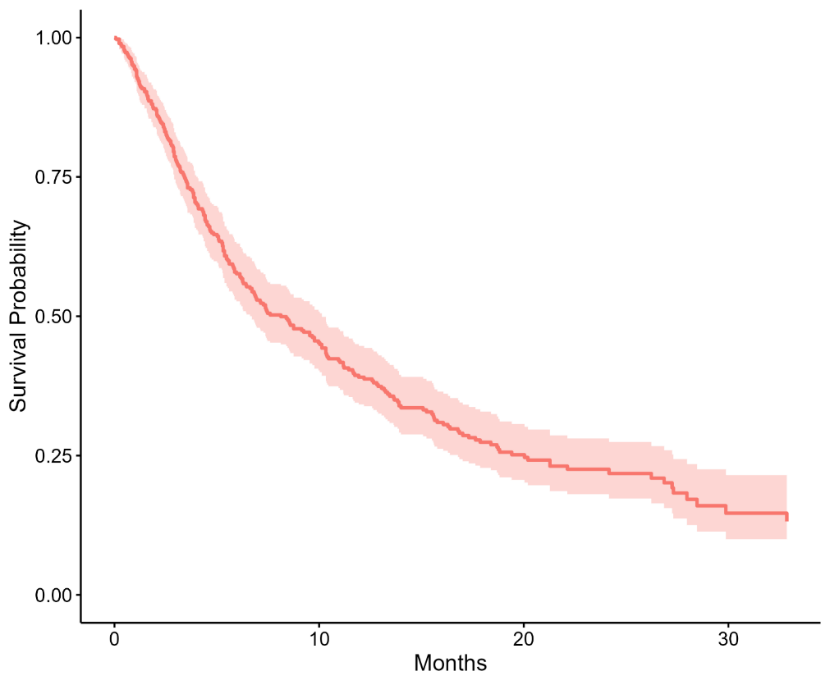


*N* = 371

Number of events: 267

Numbers censored: 104

Median (95% CI) TTNTD; months: 8.1 (6.7, 10.1)

| Months | 1 | 6 | 12 | 18 | 24 |
| --- | --- | --- | --- | --- | --- |
| *N* at risk | 349 | 197 | 118 | 65 | 30 |

TTNTD event is the earliest of a new line start or death during follow-up. Patients remaining on the line of therapy or yet to start a new line and alive at follow-up end were censored. Kaplan–Meier analysis is truncated at the point when <10 patients remain in the risk-set.

Abbreviations: 2L, second-line; CI, confidence interval; D7, DREAMM-7; D8, DREAMM-8; TTNTD, time to next treatment or death.

FIGURE S3. TTNTD from initiation of 2L by regimen

1. D7-like lenalidomide-exposed


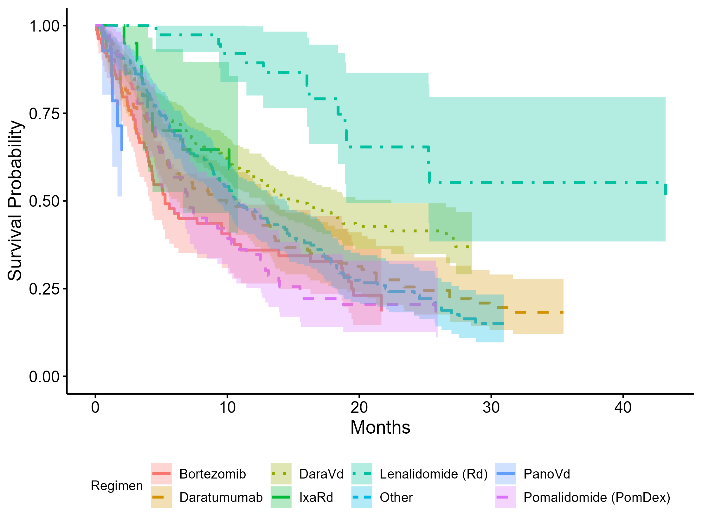


|  | **Bortezomib** | **Daratumumab** | **DaraVd** | **IxaRd** | **Rd** | **PanoVd** | **PomDex** | **Other** |
| --- | --- | --- | --- | --- | --- | --- | --- | --- |
| Total patients*, N* | 79 | 126 | 282 | 20 | 38 | 15 | 72 | 183 |
| Number of events*, n* | 63 | 99 | 135 | 11 | 17 | 14 | 61 | 143 |
| Number censored*, n* | 16 | 27 | 147 | 9 | 21 | 1 | 11 | 40 |
| Median (95% CI) TTNTD; months | 5.3 (4.1, 11.0) | 9.9 (6.2,13.6) | 15.1 (12.6, 22.4) | 28.0 (6.6, N/A) | 43.2 (19.0, N/A) | 4.0 (2.0, 11.3) | 7.1 (5.6, 10.7) | 10.6 (9.2, 14.5) |
| Months from line start | *N* at risk | | | | | | | |
| 1 | 72 | 115 | 269 | 20 | 38 | 13 | 70 | 173 |
| 6 | 34 | 71 | 177 | 13 | 37 | – | 40 | 120 |
| 12 | 23 | 51 | 114 | – | 33 | – | 23 | 79 |
| 18 | 17 | 36 | 61 | – | 18 | – | 13 | 47 |
| 24 | – | 25 | 26 | – | 13 | – | 11 | 24 |
| 36 | – | – | – | – | 10 | – | – | – |

1. D7-like lenalidomide-refractory


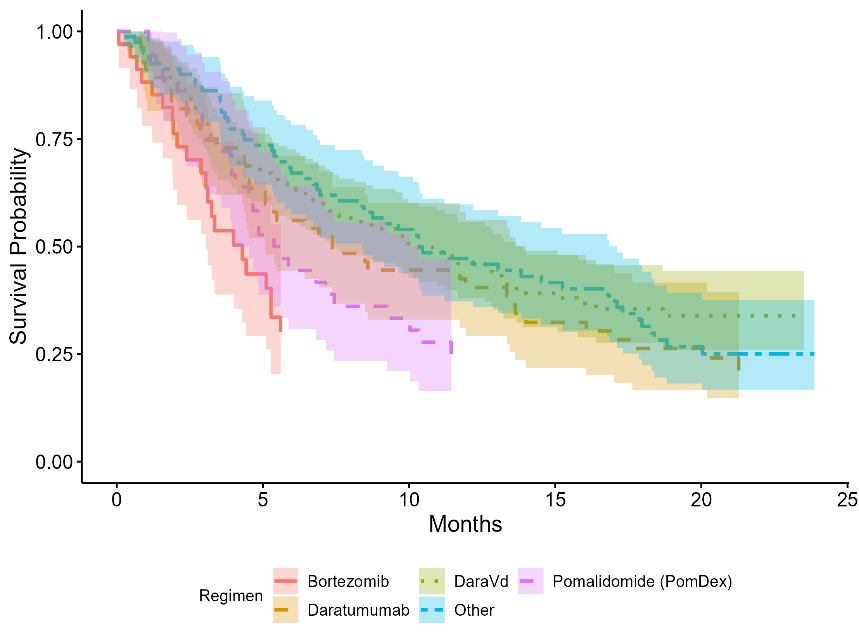


|  | **Bortezomib** | **Daratumumab** | **DaraVd** | **PomDex** | **Other** |
| --- | --- | --- | --- | --- | --- |
| Total patients*, N* | 34 | 56 | 143 | 36 | 80 |
| Number of events, *n* | 29 | 47 | 82 | 34 | 61 |
| Number censored, *n* | 5 | 9 | 61 | 2 | 19 |
| Median (95% CI) TTNTD; months | 4.3 (3.0,6.3) | 7.4 (5.1,14.0) | 10.3 (7.4, 13.9) | 5.5 (4.1,10.0) | 10.4 (8.4, 17.1) |
| Months from line start | *N* at risk | | | | |
| 1 | 30 | 51 | 135 | 36 | 76 |
| 6 | – | 30 | 80 | 16 | 52 |
| 12 | – | 20 | 49 | – | 35 |
| 18 | – | 12 | 24 | – | 21 |

1. D8-like lenalidomide-exposed


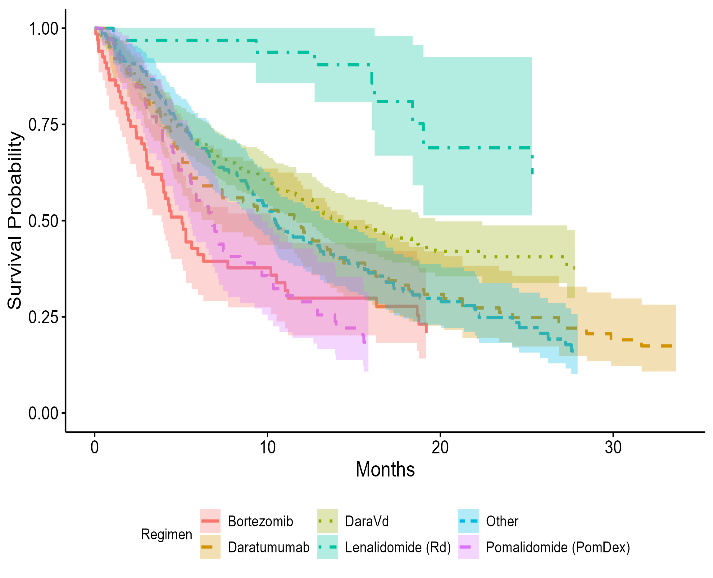


|  | **Bortezomib** | **Daratumumab** | **DaraVd** | **Rd** | **PomDex** | **Other** |
| --- | --- | --- | --- | --- | --- | --- |
| Total patients*, N* | 67 | 103 | 269 | 32 | 66 | 152 |
| Number of events, *n* | 54 | 81 | 129 | 12 | 54 | 115 |
| Number censored, *n* | 13 | 22 | 140 | 20 | 12 | 37 |
| Median (95% CI) TTNTD; months | 5 (3.9, 10.5) | 11.7 (6.9, 15.6) | 14.5 (11.7, 19.7) | 49.7 (25.3, 0) | 6.8 (5.5, 9.7) | 10.5 (9.0, 13.8) |
| Months from line start | *N* at risk | | | | | |
| 1 | 58 | 95 | 256 | 32 | 64 | 147 |
| 6 | 25 | 60 | 164 | 31 | 35 | 99 |
| 12 | 15 | 42 | 105 | 29 | 17 | 62 |
| 18 | 12 | 28 | 57 | 14 | – | 38 |
| 24 | – | 21 | 22 | 10 | – | 19 |

1. D8-like lenalidomide-refractory


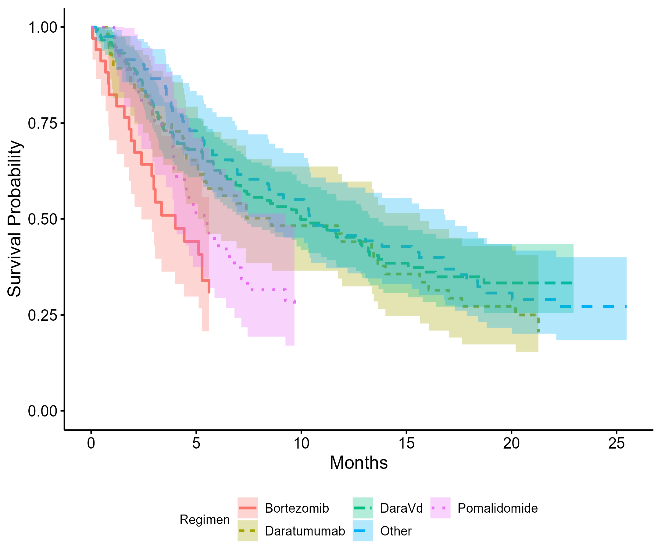


|  | **Bortezomib** | **Daratumumab** | **DaraVd** | **PomDex** | **Other** |
| --- | --- | --- | --- | --- | --- |
| Total patients*, N* | 34 | 56 | 148 | 36 | 82 |
| Number of events, *n* | 29 | 46 | 85 | 33 | 60 |
| Number censored, *n* | 5 | 10 | 63 | 3 | 22 |
| Median (95% CI) TTNTD; months | 4 (2.4, 6.3) | 8.6 (5.1, 15.6) | 10 (7.3, 13.7) | 5.4 (3.9, 9.2) | 10.4 (8.4, 16.9) |
| Months from line start | *N* at risk | | | | |
| 1 | 28 | 51 | 140 | 36 | 80 |
| 6 | – | 31 | 81 | 15 | 52 |
| 12 | – | 21 | 49 | – | 35 |
| 18 | – | 12 | 24 | – | 22 |
| 24 | – | – | – | – | 10 |

TTNTD is measured in months from start of line of therapy. TTNTD event is the earliest of a new line start or death during follow-up. Patients remaining on the line of therapy or yet to start a new line and alive at follow-up end were censored. Kaplan–Meier analysis is truncated at the point when <10 patients remain in the risk-set.

Abbreviations: 2L, second-line; CI, confidence interval; D7, DREAMM-7; D8, DREAMM-8; DaraVd, daratumumab–bortezomib–dexamethasone; IxaRd, ixazomib–lenalidomide–dexamethasone; N/A, not available; PanoVd, panobinostat–bortezomib–dexamethasone; PomDex, pomalidomide–dexamethasone; Rd, lenalidomide–dexamethasone; TTNTD, time to next treatment or death.

FIGURE S4. TTDD from initiation of 2L

1. D7-like lenalidomide-exposed


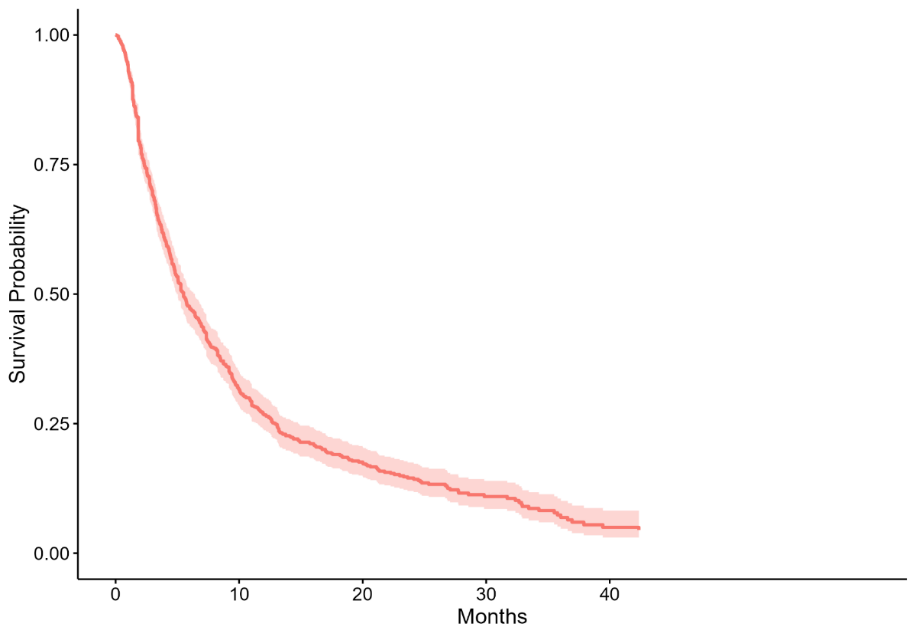


*N* = 827

Number of events: 685

Numbers censored: 142

Median (95% CI) TTDD; months: 5.5 (5.0, 6.4)

| Months | 1 | 6 | 12 | 18 | 24 | 36 |
| --- | --- | --- | --- | --- | --- | --- |
| *N* at risk | 774 | 369 | 187 | 110 | 64 | 16 |

1. D7-like lenalidomide-refractory


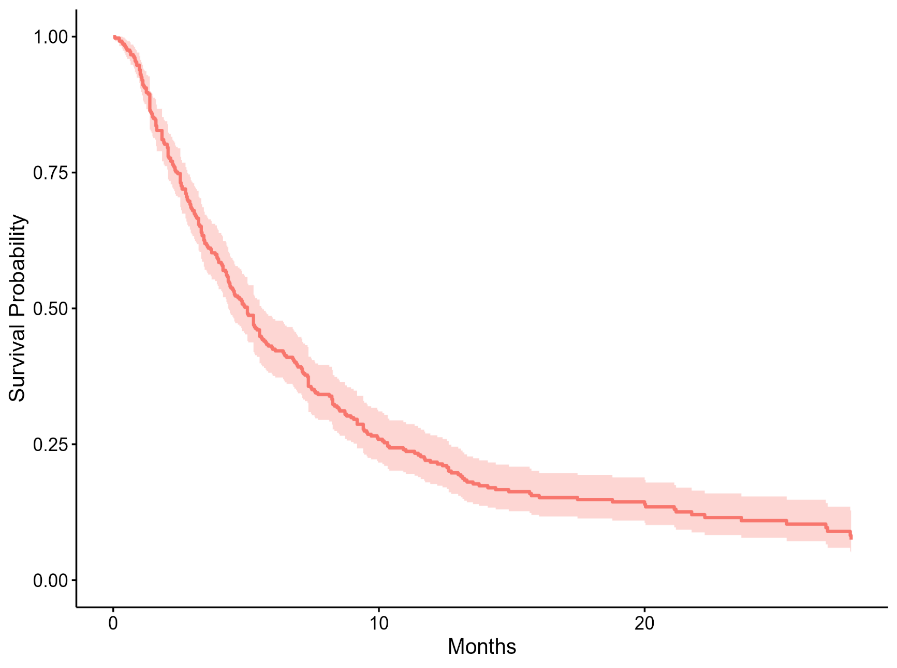


*N* = 361

Number of events: 307

Numbers censored: 54

Median (95% CI) TTDD; months: 5.0 (4.3, 5.6)

| Months | 1 | 6 | 12 | 18 | 24 |
| --- | --- | --- | --- | --- | --- |
| *N* at risk | 338 | 144 | 66 | 37 | 19 |

1. D8-like lenalidomide-exposed


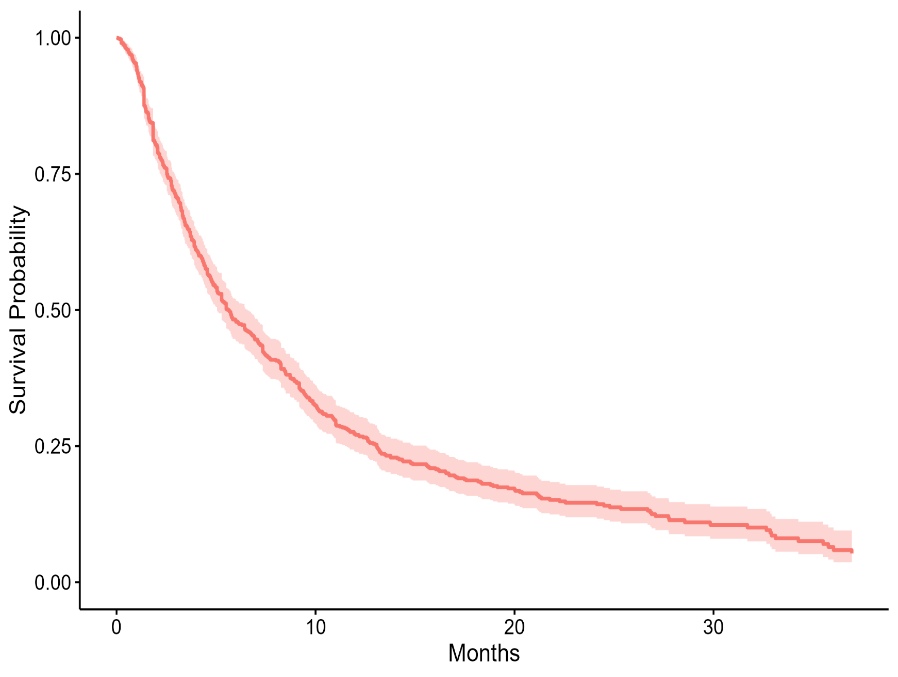


*N* = 730

Number of events: 597

Numbers censored: 133

Median (95% CI) TTDD; months: 5.6 (5.0, 6.6)

| Months | 1 | 6 | 12 | 18 | 24 | 36 |
| --- | --- | --- | --- | --- | --- | --- |
| *N* at risk | 686 | 327 | 164 | 92 | 54 | 11 |

1. D8-like lenalidomide-refractory


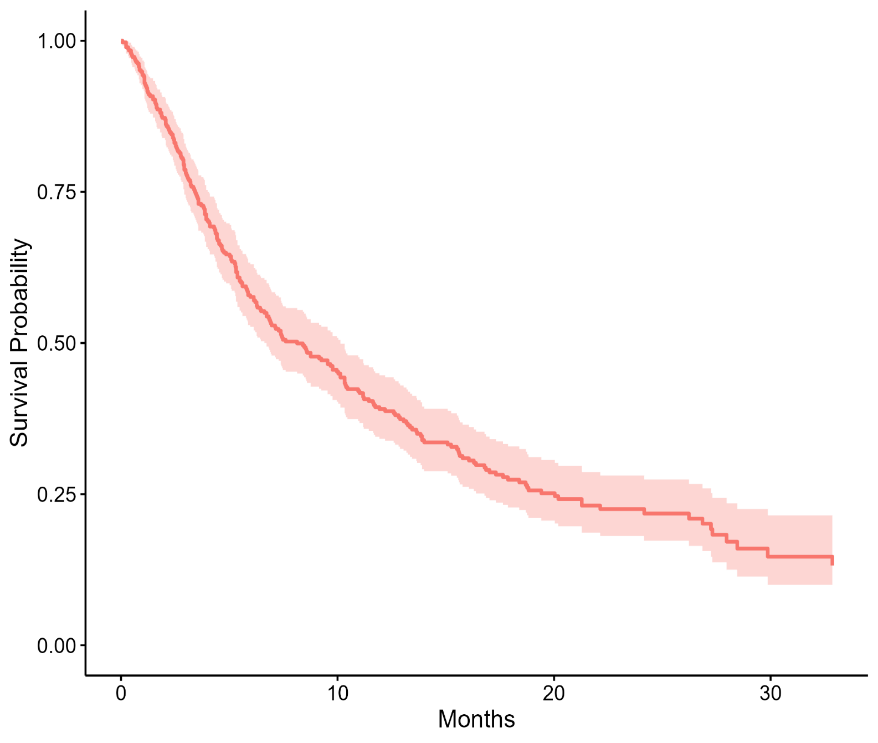


*N* = 371

Number of events: 315

Numbers censored: 56

Median (95% CI) TTDD; months: 5.0 (4.3, 5.6)

| Months | 1 | 6 | 12 | 18 | 24 |
| --- | --- | --- | --- | --- | --- |
| *N* at risk | 347 | 145 | 66 | 35 | 18 |

TTDD is measured in months from start of line of therapy. TTDD event is the earliest of: last administration plus one cycle length; start of a new line minus 1 day; death during follow-up. Patients alive at follow-up end with no subsequent line and last administration date of the line within a cycle length of administrative follow-up end were censored. Kaplan–Meier analysis is truncated at the point when <10 patients remain in the risk-set.

Abbreviations: 2L, second-line; CI, confidence interval; D7, DREAMM-7; D8, DREAMM-8; TTDD, time to treatment discontinuation or death.

FIGURE S5. TTDD from initiation of 2L by regimen

1. D7-like lenalidomide-exposed


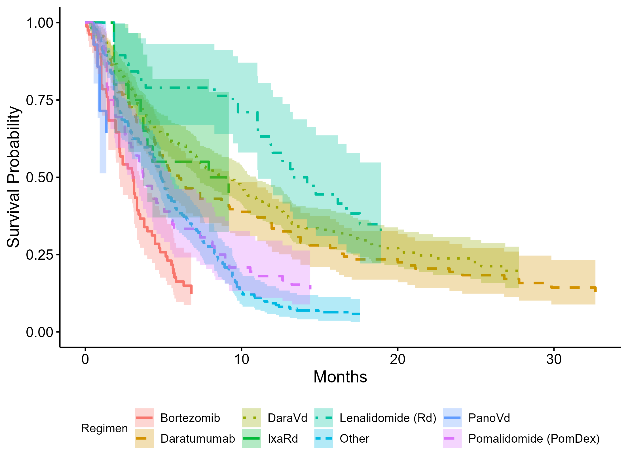


|  | **Bortezomib** | **Daratumumab** | **DaraVd** | **IxaRd** | **Rd** | **PanoVd** | **PomDex** | **Other** |
| --- | --- | --- | --- | --- | --- | --- | --- | --- |
| Total patients*, N* | 79 | 126 | 282 | 20 | 38 | 15 | 72 | 183 |
| Number of events, *n* | 75 | 106 | 188 | 16 | 33 | 14 | 69 | 173 |
| Number censored, *n* | 4 | 20 | 94 | 4 | 5 | 1 | 3 | 10 |
| Median (95% CI) TTDD; months | 3.1 (2.2, 3.5) | 6.0 (4.8, 9.2) | 8.7 (7.1, 10.9) | 8.6 (3.7, N/A) | 13.7 (11.0, 21.2) | 2.3 (1.3, 9.4) | 3.8 (2.9, 5.4) | 4.9 (4.3, 5.7) |
| Months from line start | *N* at risk | | | | | | | |
| 1 | 71 | 115 | 269 | 20 | 38 | 10 | 68 | 171 |
| 6 | 12 | 61 | 152 | 11 | 30 | – | 24 | 70 |
| 12 | – | 37 | 86 | – | 23 | – | 13 | 16 |
| 18 | – | 25 | 48 | – | 10 | – | – | – |
| 24 | – | 18 | 23 | – | – | – | – | – |

1. D7-like lenalidomide-refractory


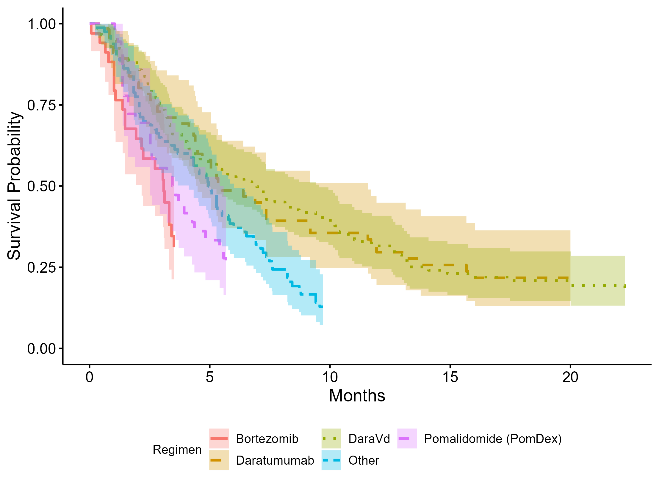


|  | **Bortezomib** | **Daratumumab** | **DaraVd** | **PomDex** | **Other** |
| --- | --- | --- | --- | --- | --- |
| Total patients*, N* | 34 | 56 | 143 | 36 | 80 |
| Number of events, *n* | 30 | 49 | 105 | 34 | 78 |
| Number censored, *n* | 4 | 7 | 38 | 2 | 2 |
| Median (95% CI) TTDD; months | 3.1 (2.2, 4.0) | 5.5 (4.5, 11.6) | 7.0 (4.9, 9.5) | 3.5 (2.5, 5.4) | 5.0 (4.1, 6.0) |
| Months from line start | *N* at risk | | | | |
| 1 | 30 | 51 | 135 | 36 | 75 |
| 6 | – | 26 | 68 | – | 30 |
| 12 | – | 15 | 35 | – | – |
| 18 | – | 10 | 18 | – | – |

1. D8-like lenalidomide-exposed


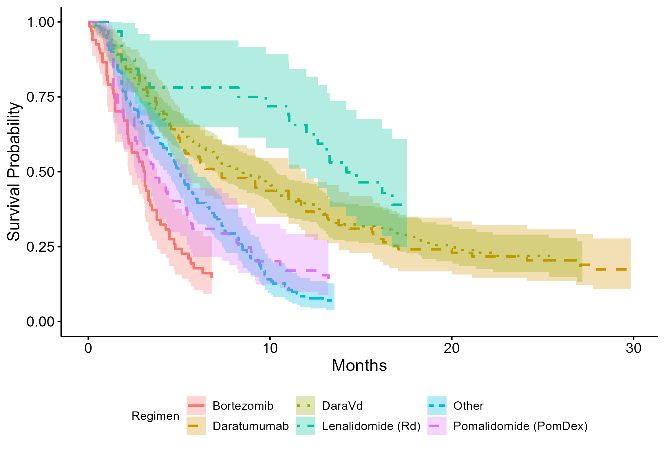


|  | **Bortezomib** | **Daratumumab** | **DaraVd** | **Rd** | **PomDex** | **Other** |
| --- | --- | --- | --- | --- | --- | --- |
| Total patients*, N* | 67 | 103 | 269 | 32 | 66 | 152 |
| Number of events, *n* | 63 | 85 | 181 | 27 | 62 | 144 |
| Number censored, *n* | 4 | 18 | 88 | 5 | 4 | 8 |
| Median (95% CI) TTDD; months | 3.1 (2.2, 3.8) | 6.9 (5.1, 11.9) | 8.5 (7, 10.3) | 14.2 (12, 21.2) | 3.7 (2.6, 5.4) | 5 (4.3, 5.8) |
| Months from line start | *N* at risk | | | | | |
| 1 | 58 | 95 | 256 | 32 | 63 | 145 |
| 6 | 11 | 54 | 141 | 25 | 20 | 60 |
| 12 | – | 32 | 79 | 21 | 11 | 12 |
| 18 | – | 20 | 43 | – | – | – |
| 24 | – | 17 | 19 | – | – | – |

1. D8-like lenalidomide-refractory


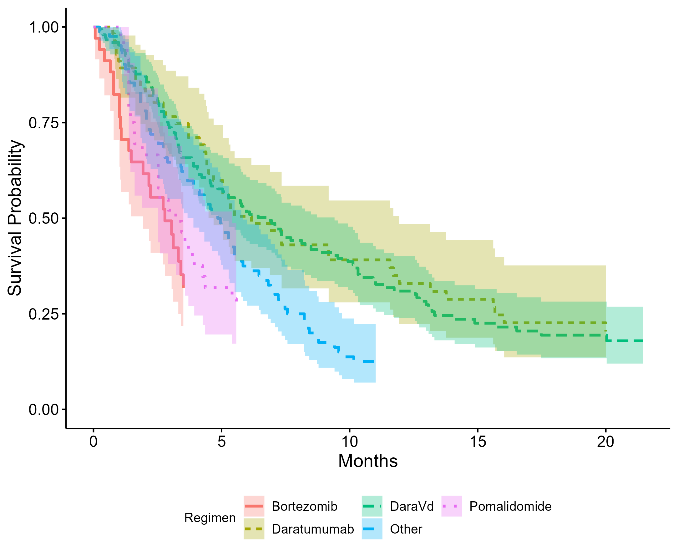


|  | **Bortezomib** | **Daratumumab** | **DaraVd** | **PomDex** | **Other** |
| --- | --- | --- | --- | --- | --- |
| Total patients*, N* | 34 | 56 | 148 | 36 | 82 |
| Number of events, *n* | 30 | 48 | 110 | 33 | 80 |
| Number censored, *n* | 4 | 8 | 38 | 3 | 2 |
| Median (95% CI) TTDD; months | 2.8 (1.9, 4) | 6.2 (4.7, 11.9) | 6.8 (5, 9.4) | 3.2 (2.5, 5.4) | 4.9 (3.9, 5.8) |
| Months from line start | *N* at risk | | | | |
| 1 | 28 | 51 | 140 | 36 | 78 |
| 6 | – | 27 | 69 | – | 30 |
| 12 | – | 16 | 35 | – | – |
| 18 | – | 10 | 17 | – | – |

TTDD is measured in months from start of line of therapy. TTDD event is the earliest of: last administration plus one cycle length; start of a new line minus 1 day; death during follow-up. Patients alive at follow-up end with no subsequent line and last administration date of the line within a cycle length of administrative follow-up end were censored. Kaplan–Meier analysis is truncated at the point when <10 patients remain in the risk-set.

Abbreviations: 2L, second-line; CI, confidence interval; D7, DREAMM-7; D8, DREAMM-8; DaraVd, daratumumab–bortezomib–dexamethasone; IxaRd, ixazomib–lenalidomide–dexamethasone; N/A, not available; PanoVd, panobinostat–bortezomib–dexamethasone; PomDex, pomalidomide–dexamethasone; Rd, lenalidomide–dexamethasone; TTDD, time to treatment discontinuation or death.

FIGURE S6. OS from initiation of 2L

1. D7-like lenalidomide-exposed


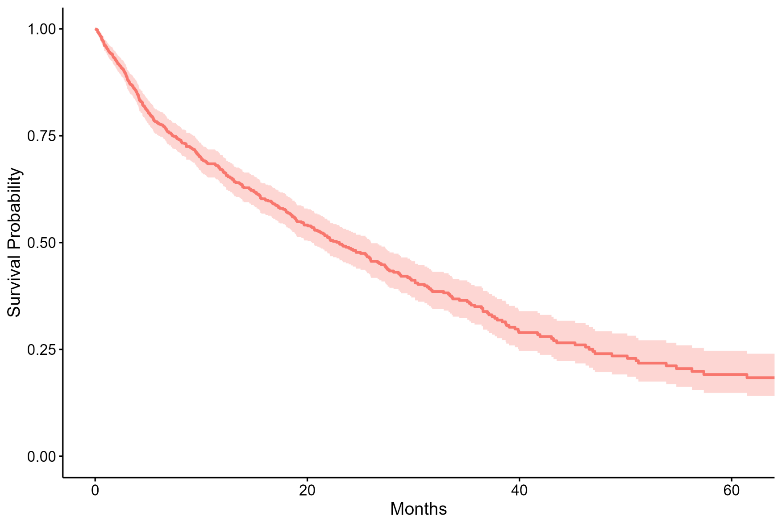


*N* = 827

Number of events: 446

Numbers censored: 381

Median (95% CI) OS; months: 23.0 (20.4, 26.0)

| Months | 1 | 6 | 12 | 18 | 24 | 36 | 48 | 60 |
| --- | --- | --- | --- | --- | --- | --- | --- | --- |
| *N* at risk | 787 | 599 | 460 | 317 | 204 | 93 | 45 | 26 |

1. D7-like lenalidomide-refractory


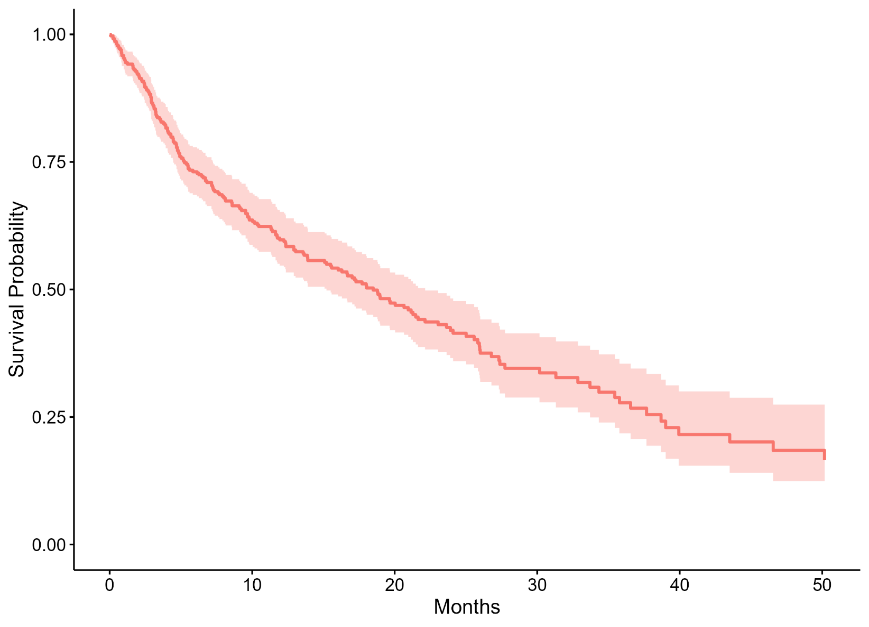


*N* = 361

Number of events: 211

Numbers censored: 150

Median (95% CI) OS; months: 18.5 (15.2, 22.1)

| Months | 1 | 6 | 12 | 18 | 24 | 36 | 48 |
| --- | --- | --- | --- | --- | --- | --- | --- |
| *N* at risk | 343 | 246 | 181 | 126 | 73 | 26 | 10 |

1. D8-like lenalidomide-exposed


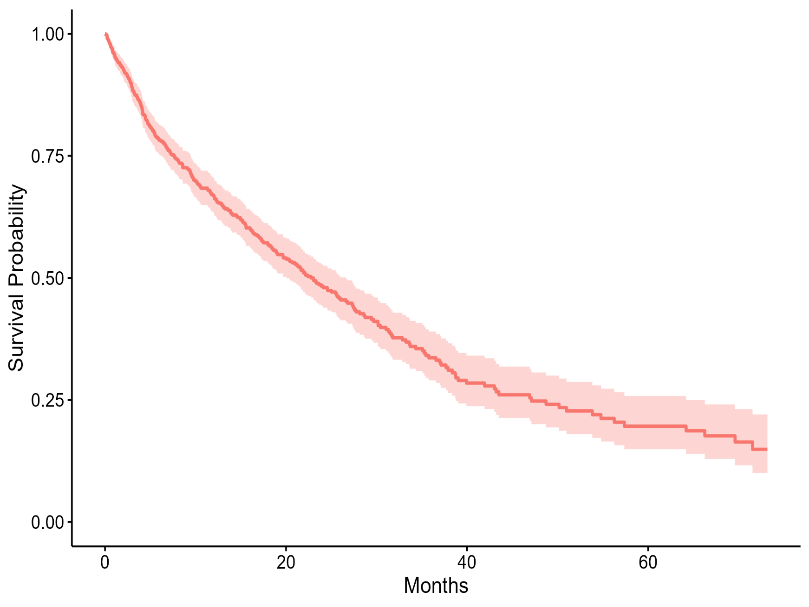


*N* = 730

Number of events: 380

Numbers censored: 350

Median (95% CI) OS; months: 23.0 (20.0, 26.6)

| Months | 1 | 6 | 12 | 18 | 24 | 36 | 48 | 60 |
| --- | --- | --- | --- | --- | --- | --- | --- | --- |
| *N* at risk | 694 | 526 | 395 | 265 | 166 | 70 | 38 | 23 |

1. D8-like lenalidomide-refractory


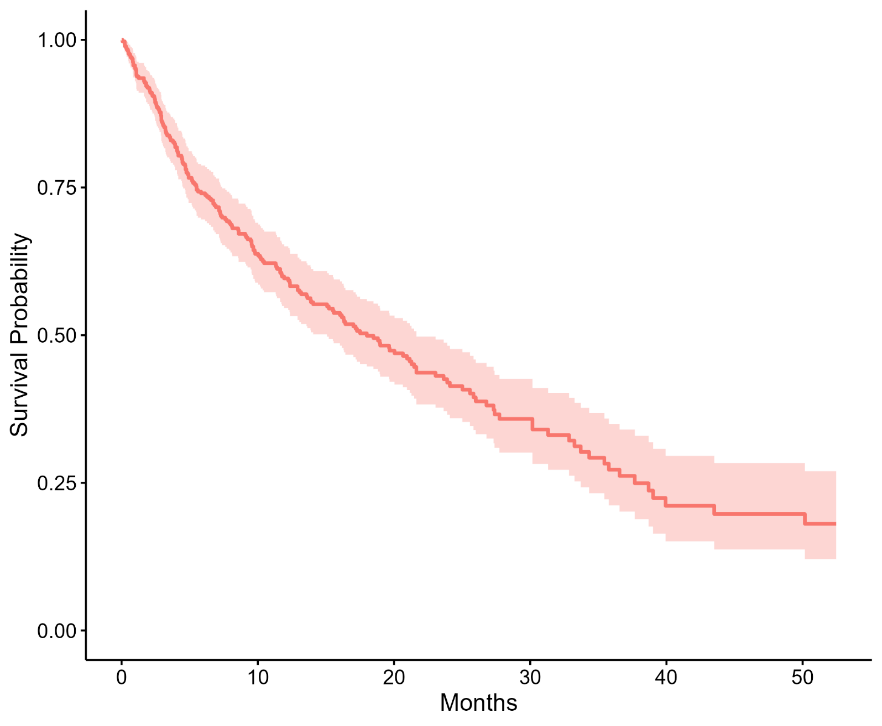


*N* = 371

Number of events: 214

Numbers censored: 157

Median (95% CI) OS; months: 18.0 (15.1, 21.6)

| Months | 1 | 6 | 12 | 18 | 24 | 36 | 48 |
| --- | --- | --- | --- | --- | --- | --- | --- |
| *N* at risk | 352 | 254 | 182 | 124 | 72 | 26 | 12 |

OS is measured in months from start of line of therapy. OS event is death during follow-up. Patients alive at follow-up were censored. Kaplan–Meier analysis is truncated at the point when <10 patients remain in the risk-set.

Abbreviations: 2L, second-line; CI, confidence interval; D7, DREAMM-7; D8, DREAMM-8; OS, overall survival.

FIGURE S7. OS from initiation of 2L by regimen

1. D7-like lenalidomide-exposed


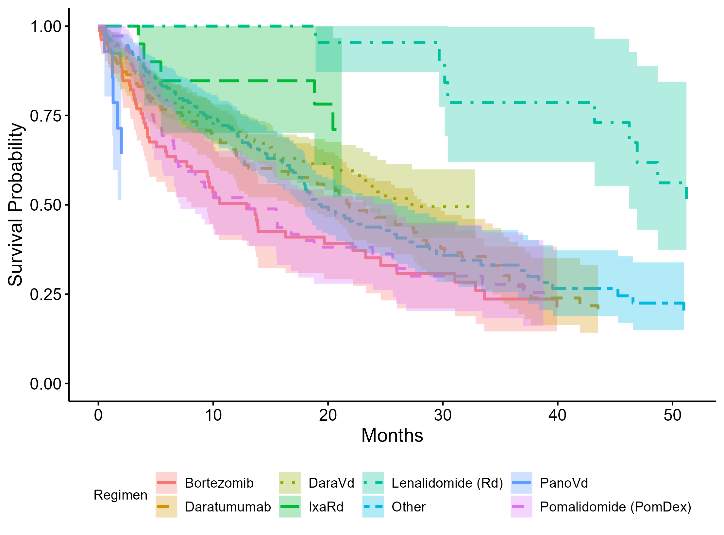


|  | **Bortezomib** | **Daratumumab** | **DaraVd** | **IxaRd** | **Rd** | **PanoVd** | **PomDex** | **Other** |
| --- | --- | --- | --- | --- | --- | --- | --- | --- |
| Total patients*, N* | 79 | 126 | 282 | 20 | 38 | 15 | 72 | 183 |
| Number of events, *n* | 57 | 84 | 100 | 7 | 11 | 14 | 53 | 111 |
| Number censored, *n* | 22 | 42 | 182 | 13 | 27 | 1 | 19 | 72 |
| Median (95% CI) OS; months | 12.9 (8.1, 23.3) | 21.9 (17.0, 28.6) | 27.3 (23.0, N/A) | 53.8 (35.4, N/A) | 79.8 (46.2, N/A) | 4.1 (2.0, 15.0) | 13.1 (7.1, 25.6) | 19.4 (17.7, 26.0) |
| Months from line start | *N* at risk | | | | | | | |
| 1 | 74 | 116 | 269 | 20 | 38 | 13 | 70 | 175 |
| 6 | 47 | 93 | 195 | 16 | 38 | – | 48 | 145 |
| 12 | 33 | 73 | 141 | 13 | 37 | – | 33 | 117 |
| 18 | 23 | 64 | 81 | 13 | 24 | – | 22 | 81 |
| 24 | 17 | 50 | 36 | – | 19 | – | 19 | 47 |
| 36 | 10 | 19 | – | – | 14 | – | 12 | 21 |
| 48 | – | – | – | – | 11 | – | – | 11 |

1. D7-like lenalidomide-refractory


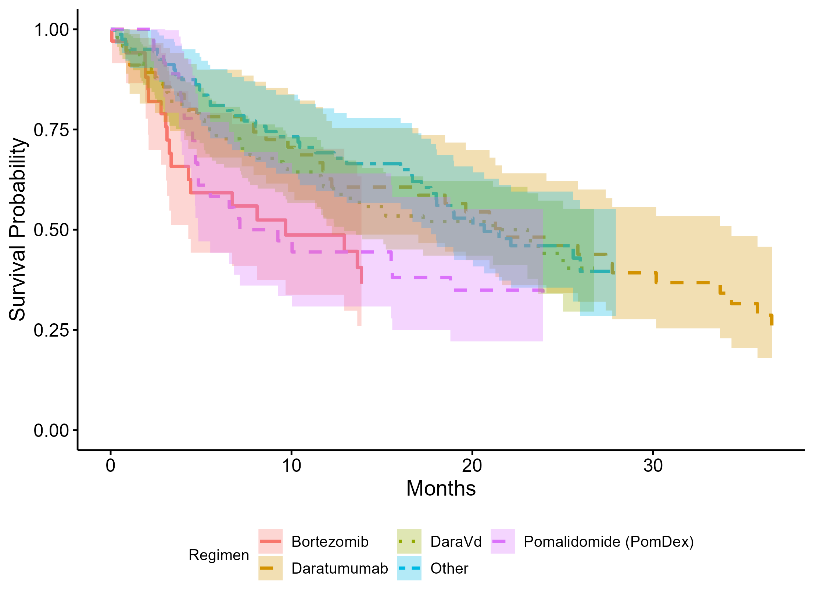


|  | **Bortezomib** | **Daratumumab** | **DaraVd** | **PomDex** | **Other** |
| --- | --- | --- | --- | --- | --- |
| Total patients*, N* | 34 | 56 | 143 | 36 | 80 |
| Number of events, *n* | 26 | 38 | 65 | 29 | 44 |
| Number censored, *n* | 8 | 18 | 78 | 7 | 36 |
| Median (95% CI) OS; months | 9.7 (4.3, 19.7) | 21.6 (12.2, 34.3) | 23.0 (12.9, N/A) | 8.2 (4.8, 25.9) | 20.7 (17.7, N/A) |
| Months from line start | *N* at risk | | | | |
| 1 | 32 | 52 | 135 | 36 | 77 |
| 6 | 18 | 42 | 93 | 21 | 63 |
| 12 | 12 | 31 | 65 | 16 | 51 |
| 18 | – | 28 | 34 | 12 | 39 |
| 24 | – | 23 | 14 | – | 19 |
| 36 | – | 10 | – | – | – |

1. D8-like lenalidomide-exposed


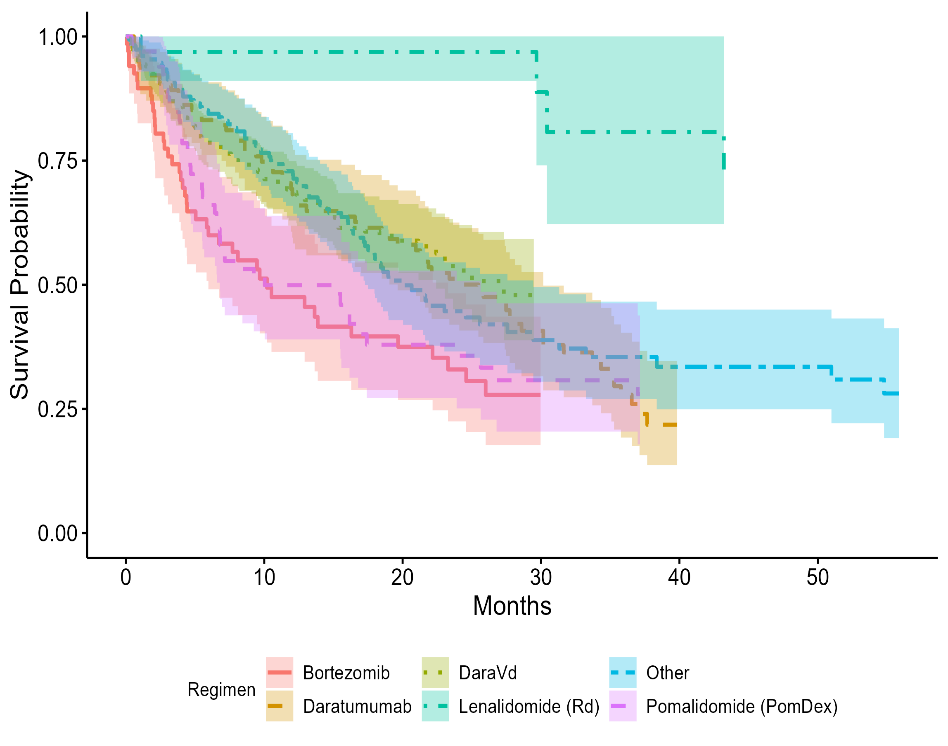


|  | **Bortezomib** | **Daratumumab** | **DaraVd** | **Rd** | **PomDex** | **Other** |
| --- | --- | --- | --- | --- | --- | --- |
| Total patients*, N* | 67 | 103 | 269 | 32 | 66 | 152 |
| Number of events, *n* | 48 | 68 | 97 | 8 | 47 | 84 |
| Number censored, *n* | 19 | 35 | 172 | 24 | 19 | 68 |
| Median (95% CI) OS; months | 10.2 (6.0, 23.3) | 25.5 (19.6, 30.2) | 27.3 (22.4, 0) | 79.8 (46.9, 0) | 10 (6.8, 25.6) | 19 (17.3, 29.4) |
| Months from line start | *N* at risk | | | | | |
| 1 | 60 | 96 | 256 | 32 | 64 | 147 |
| 6 | 37 | 82 | 184 | 31 | 42 | 121 |
| 12 | 25 | 62 | 130 | 30 | 28 | 98 |
| 18 | 19 | 54 | 74 | 17 | 18 | 65 |
| 24 | 14 | 44 | 30 | 14 | 16 | 36 |
| 36 | – | 15 | – | 10 | – | 19 |
| 48 | – | – | – | – | – | 14 |

1. D8-like lenalidomide-refractory


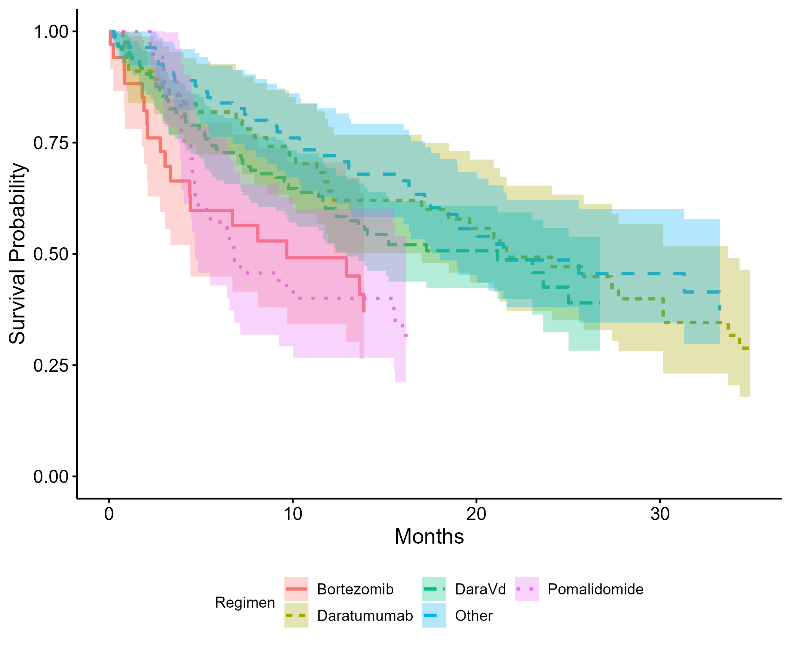


|  | **Bortezomib** | **Daratumumab** | **DaraVd** | **PomDex** | **Other** |
| --- | --- | --- | --- | --- | --- |
| Total patients*, N* | 34 | 56 | 148 | 36 | 82 |
| Number of events, *n* | 25 | 38 | 68 | 29 | 42 |
| Number censored, *n* | 9 | 18 | 80 | 7 | 40 |
| Median (95% CI) OS; months | 9.7 (4.4, 26.0) | 21.6 (17.0, 33.7) | 21.1 (12.9, N/A) | 6.8 (4.7, 23.9) | 21.6 (18.0, N/A) |
| Months from line start | *N* at risk | | | | |
| 1 | 30 | 52 | 140 | 36 | 80 |
| 6 | 18 | 44 | 96 | 20 | 66 |
| 12 | 12 | 31 | 66 | 14 | 53 |
| 18 | – | 28 | 34 | – | 39 |
| 24 | – | 23 | 13 | – | 20 |

OS is measured in months from start of line of therapy. OS event is death during follow-up. Patients alive at follow-up were censored. Kaplan–Meier analysis is truncated at the point when <10 patients remain in the risk-set.

Abbreviations: 2L, second-line; CI, confidence interval; D7, DREAMM-7; D8, DREAMM-8; DaraVd, daratumumab–bortezomib–dexamethasone; IxaRd, ixazomib–lenalidomide–dexamethasone; N/A, not available; OS, overall survival; PanoVd, panobinostat–bortezomib–dexamethasone; PomDex, pomalidomide–dexamethasone; Rd, lenalidomide–dexamethasone.

FIGURE S8. TTNTD from initiation of 3L+, 4L+ and 5L+ by regimen

1. DR3L+


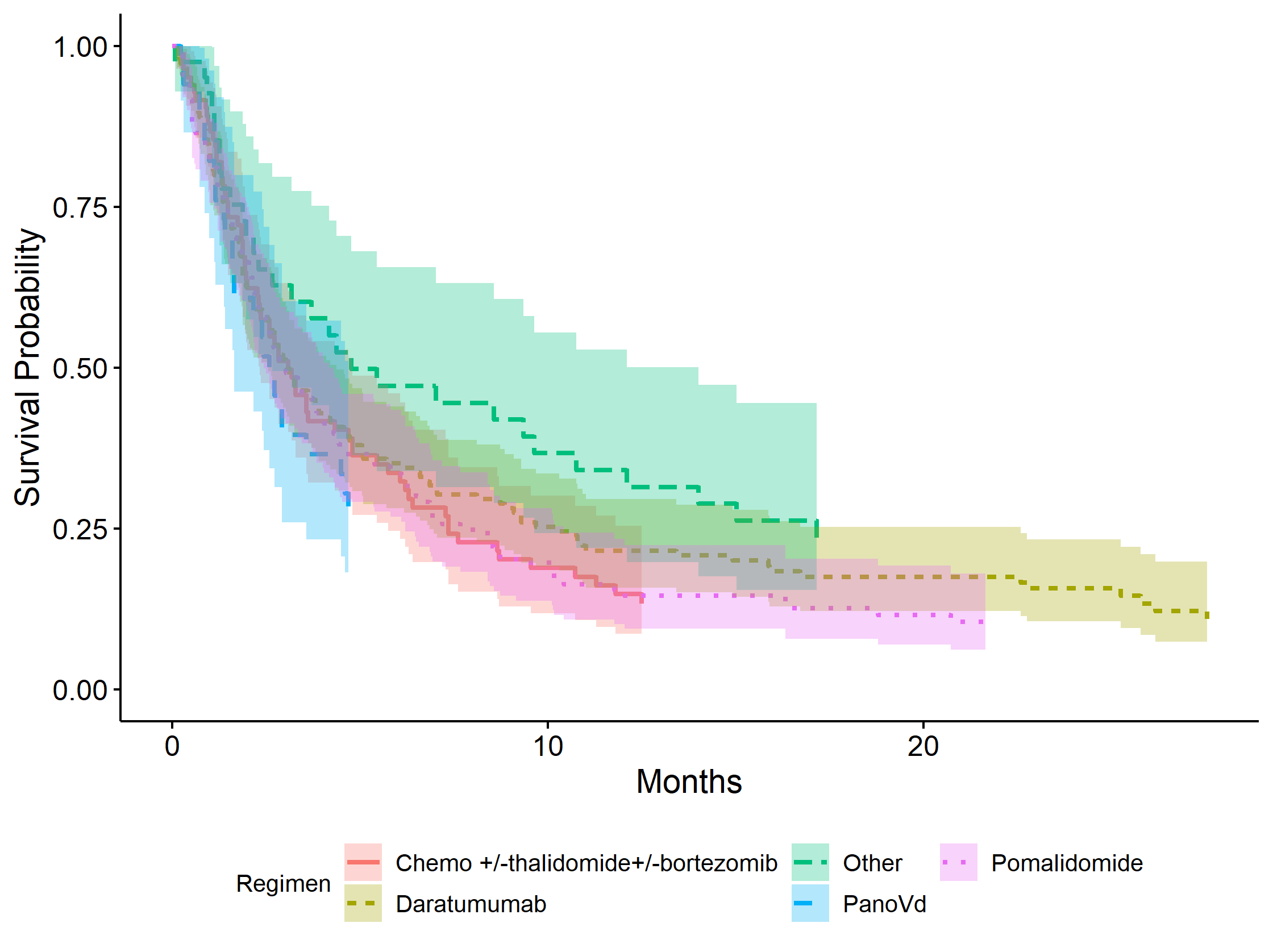


|  | **PomDex** | **PanoVd** | **Daratumumab** | **Chemotherapy ± thalidomide ± bortezomib** | **Other** |
| --- | --- | --- | --- | --- | --- |
| Total patients*, N* | 133 | 34 | 145 | 83 | 41 |
| Number of events, *n* | 121 | 32 | 125 | 73 | 36 |
| Number censored, *n* | 12 | 2 | 20 | 10 | 5 |
| Median (95% CI) TTNTD; months | 2.9 (2.4, 4.2) | 2.6 (1.6, 4.7) | 3.0 (2.6, 4.4) | 3.1 (2.3, 4.8) | 4.8 (2.7, 14.0) |
| Months from line start | *N* at risk | | | | |
| 1 | 107 | 27 | 120 | 73 | 38 |
| 6 | 42 | – | 50 | 25 | 18 |
| 12 | 18 | – | 29 | 11 | 13 |
| 18 | 12 | – | 21 | – | – |
| 24 | – | – | 16 | – | – |

1. DR4L+


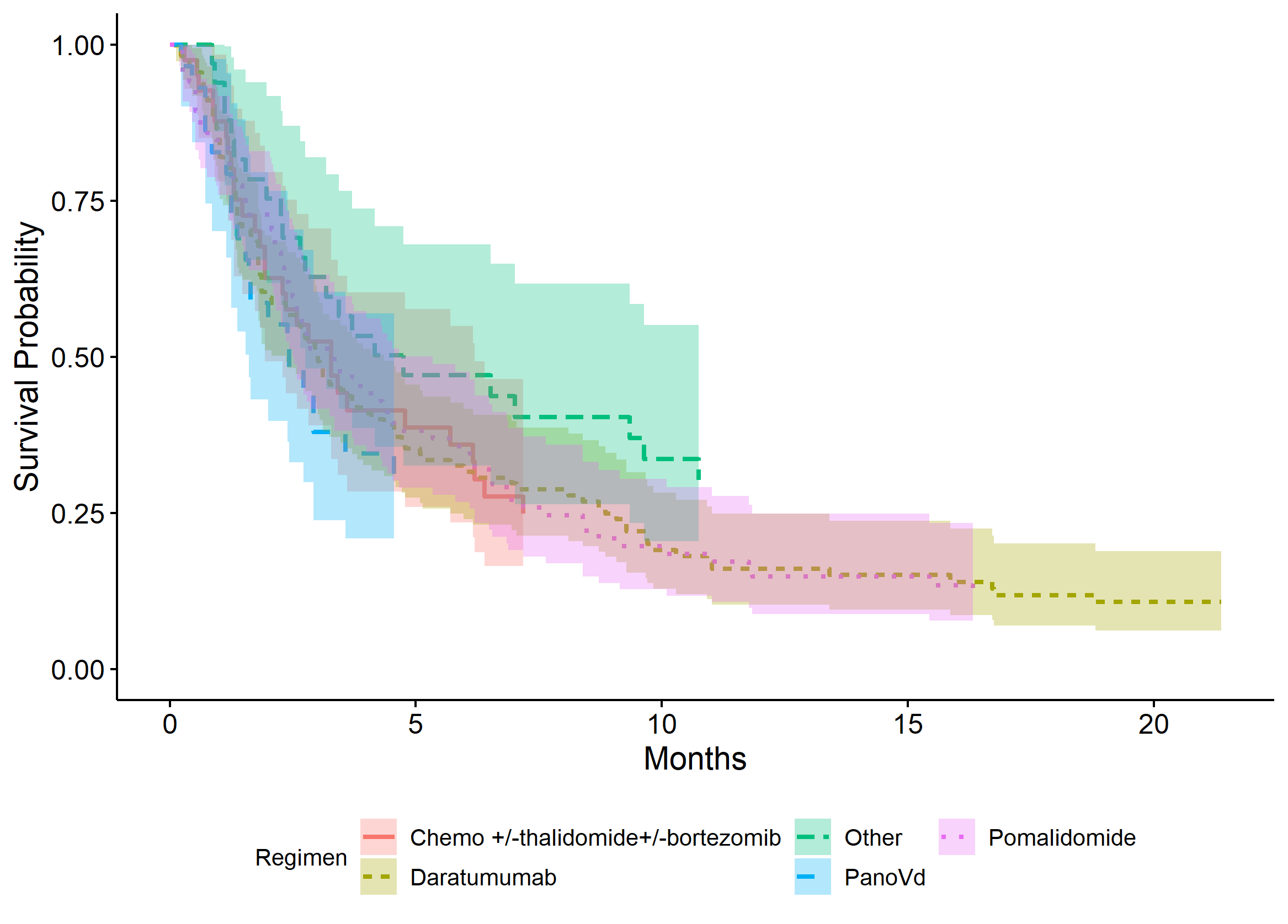


|  | **PomDex** | **PanoVd** | **Daratumumab** | **Chemotherapy ± thalidomide ± bortezomib** | **Other** |
| --- | --- | --- | --- | --- | --- |
| Total patients*, N* | 85 | 29 | 112 | 41 | 33 |
| Number of events, *n* | 81 | 29 | 99 | 36 | 29 |
| Number censored, *n* | 4 | 0 | 13 | 5 | 4 |
| Median (95% CI) TTNTD; months | 3.3 (2.5, 5.3) | 2.4 (1.6, 5.4) | 3.0 (2.4, 4.4) | 3.3 (1.9, 6.2) | 4.8 (2.8, 15.0) |
| Months from line start | *N* at risk | | | | |
| 1 | 70 | 24 | 94 | 35 | 31 |
| 6 | 29 | – | 34 | 13 | 14 |
| 12 | 12 | – | 16 | – | – |
| 18 | – | – | 11 | – | – |

1. TR5L+


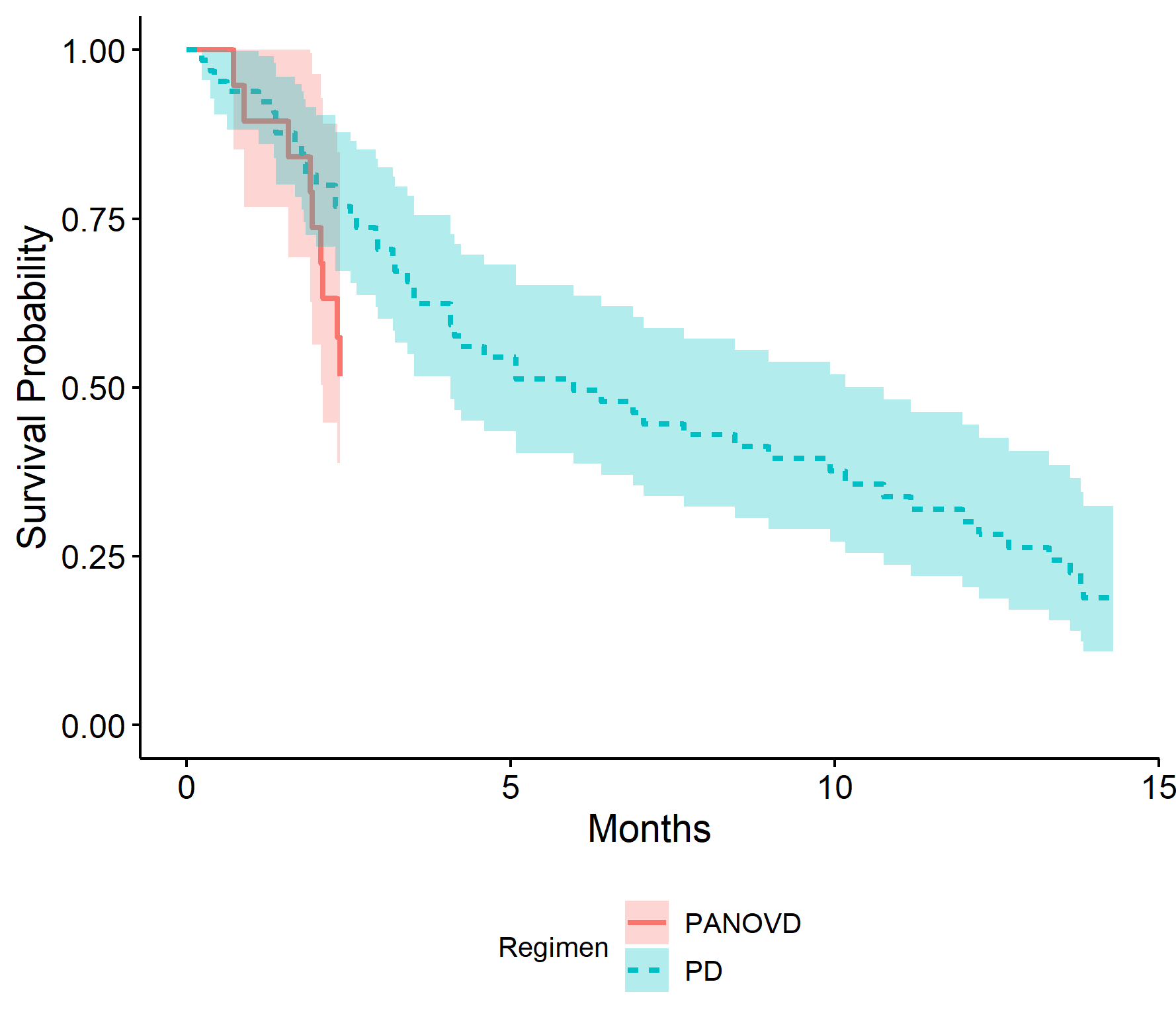


|  | **PomDex** | **PanoVd** |
| --- | --- | --- |
| Total patients*, N* | 65 | 19 |
| Number of events, *n* | 53 | 16 |
| Number censored, *n* | 12 | 3 |
| Median (95% CI) TTNTD; months | 6.0 (4.1, 10.8) | 3.0 (2.1, 6.6) |
| Months from line start | *N* at risk | |
| 1 | 61 | 17 |
| 3 | 44 | – |
| 6 | 30 | – |
| 9 | 22 | – |
| 12 | 16 | – |

DR3L+ = double class refractory MM commencing a third or higher line of therapy. DR4L+ = double class refractory MM commencing a fourth or higher line of therapy. TR5L+ = triple class refractory MM commencing a fifth or higher line of therapy. Patients at DR3L+, DR4L+ and TR5L+ do not necessarily meet the D7-like and D8-like eligibility criteria. TTNTD is measured in months from start of line of therapy. TTNTD event is the earliest of a new line start or death during follow-up. Patients remaining on the line of therapy or yet to start a new line and alive at follow-up end were censored. Kaplan–Meier analysis is truncated at the point when <10 patients remain in the risk-set.

Abbreviations: 3L, third-line; 4L, fourth-line; 5L, fifth-line; CI, confidence interval; chemo, chemotherapy; D7, DREAMM-7; D8, DREAMM-8; DR, double class refractory; MM, multiple myeloma; PanoVd, panobinostat–bortezomib–dexamethasone; PomDex, pomalidomide–dexamethasone; TR, triple class refractory; TTNTD, time to next treatment or death.

FIGURE S9. TTDD from initiation of 3L+ and 4L+ by regimen

1. DR3L+


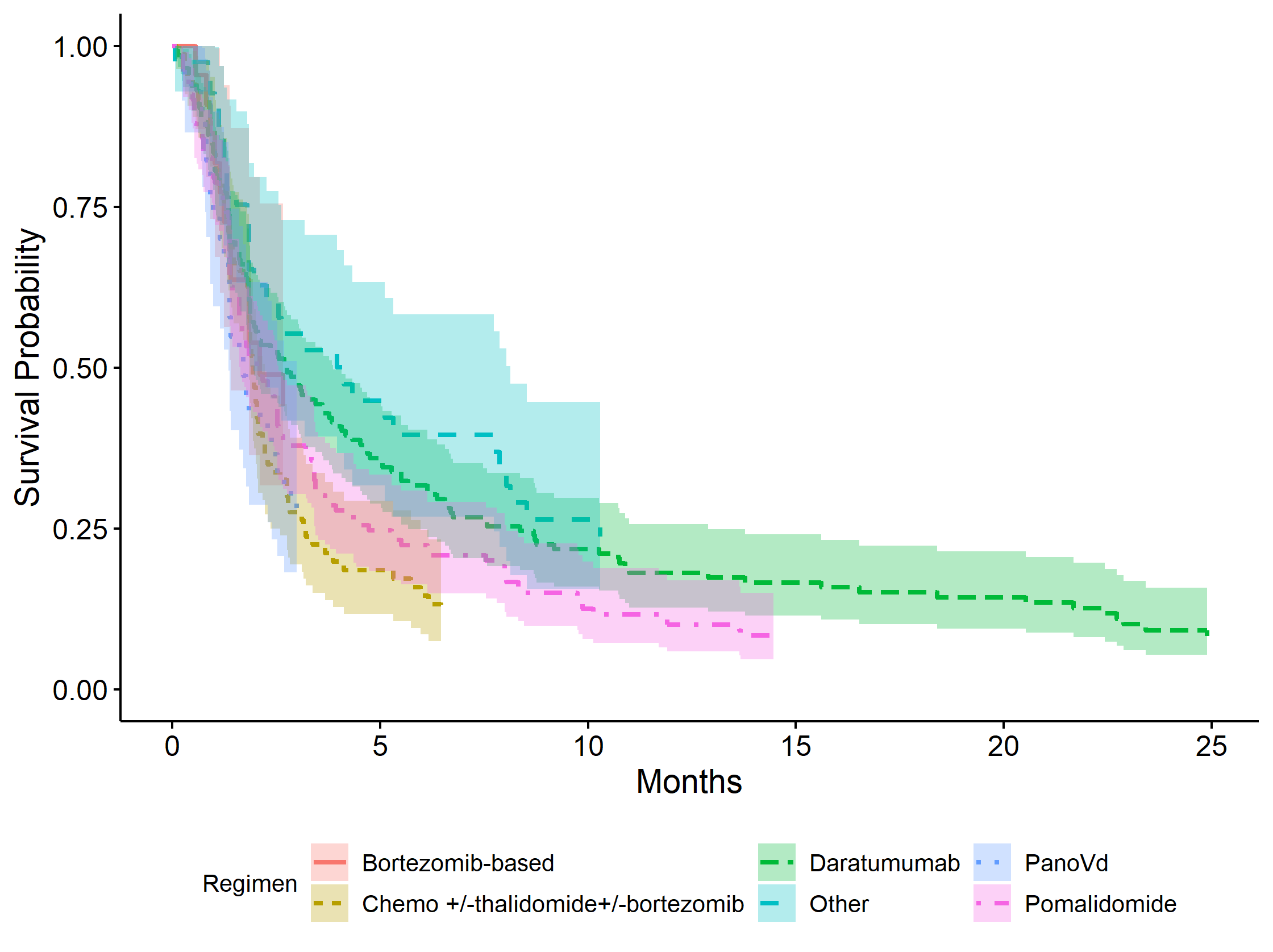


|  | **PomDex** | **PanoVd** | **Daratumumab** | **Chemotherapy ± thalidomide ± bortezomib** | **Bortezomib-based** | **Other** |
| --- | --- | --- | --- | --- | --- | --- |
| Total patients*, N* | 133 | 34 | 145 | 83 | 22 | 41 |
| Number of events, *n* | 127 | 33 | 134 | 80 | 21 | – |
| Number censored, *n* | 6 | 1 | 11 | 3 | 1 | – |
| Median (95% CI) TTDD; months | 2.1 (1.6, 2.6) | 1.7 (1.3, 3.0) | 2.7 (1.9, 3.9) | 1.9 (1.8, 2.2) | 2.1 (1.4, 4.5) | 4.1 (2.0, 8.1) |
| Months from line start | *N* at risk | | | | | |
| 1 | 104 | 24 | 120 | 73 | 19 | 38 |
| 6 | 29 | – | 45 | 11 | – | 15 |
| 12 | 12 | – | 24 | – | – | – |
| 18 | – | – | 19 | – | – | – |
| 24 | – | – | 10 | – | – | – |

1. DR4L+


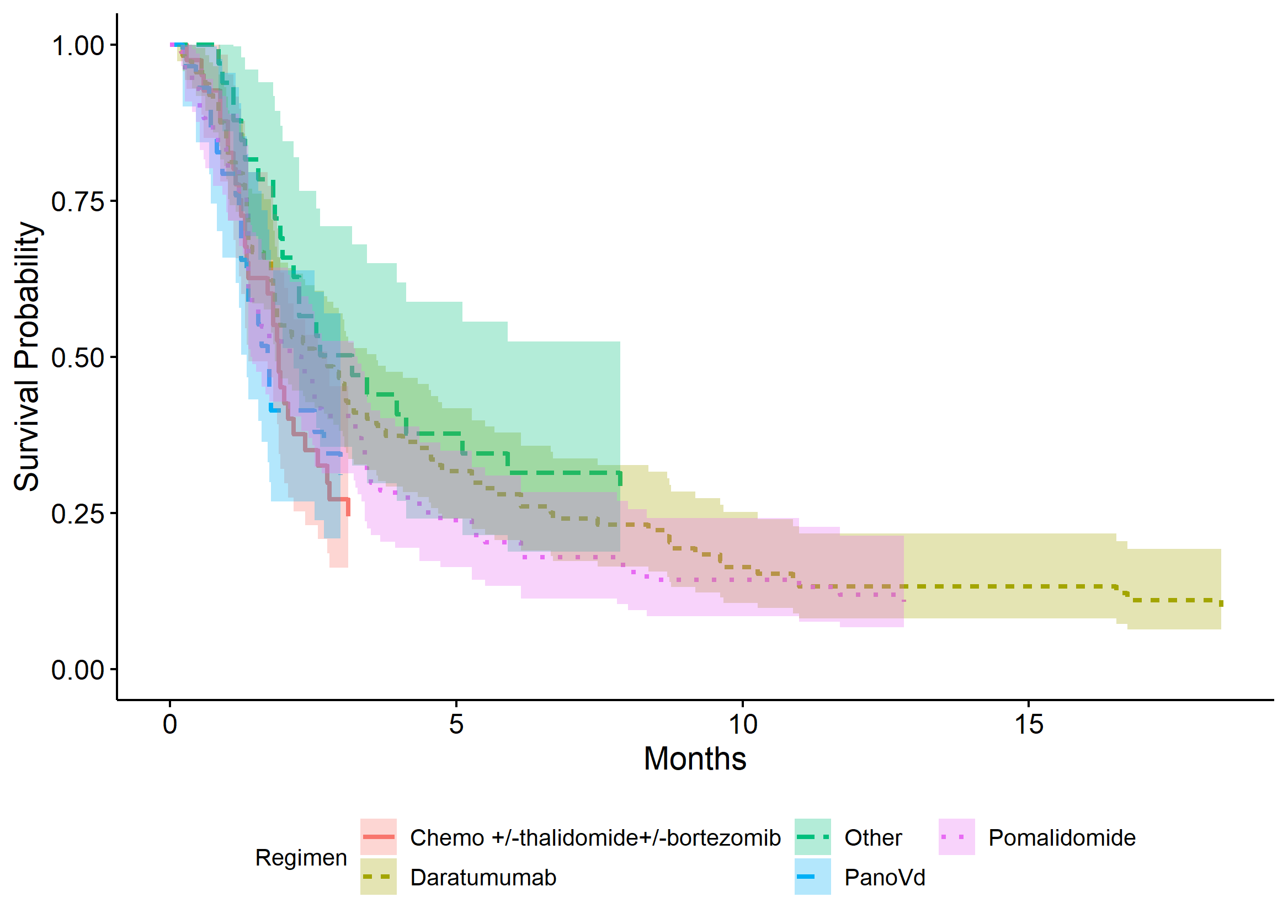


|  | **PomDex** | **PanoVd** | **Daratumumab** | **Chemotherapy ± thalidomide ± bortezomib** | **Other** |
| --- | --- | --- | --- | --- | --- |
| Total patients*, N* | 85 | 29 | 112 | 41 | 33 |
| Number of events, *n* | 83 | 29 | 102 | 38 | 31 |
| Number censored, *n* | 2 | 0 | 10 | 3 | 2 |
| Median (95% CI) TTDD; months | 2.3 (1.4, 3.3) | 1.7 (1.3, 3.2) | 2.7 (1.8, 3.6) | 1.9 (1.4, 2.8) | 3.2 (2.2, 7.9) |
| Months from line start | *N* at risk | | | | |
| 1 | 68 | 23 | 94 | 35 | 31 |
| 6 | 17 | – | 30 | – | 10 |
| 12 | 10 | – | 13 | – | – |
| 18 | – | – | 10 | – | – |

DR3L+ = double class refractory MM commencing a third or higher line of therapy. DR4L+ = double class refractory MM commencing a fourth or higher line of therapy. Patients at DR3L+ and DR4L+ do not necessarily meet the D7-like and D8-like eligibility criteria. TTDD is measured in months from start of line of therapy. TTDD event is the earliest of last administration plus one cycle length; start of a new line minus 1 day; death during follow-up. Those alive at follow-up end with no subsequent line and last administration date of the index line within a cycle length of administrative follow-up end were censored. Kaplan–Meier analysis is truncated at the point when <10 patients remain in the risk-set.

Abbreviations: 3L, third-line; 4L, fourth-line; CI, confidence interval; chemo, chemotherapy; D7, DREAMM-7; D8, DREAMM-8; DR, double class refractory; MM, multiple myeloma; PanoVd, panobinostat–bortezomib–dexamethasone; PomDex, pomalidomide–dexamethasone; TTDD, time to treatment discontinuation or death.

FIGURE S10. OS from initiation 3L+, 4L+ and 5L+ by regimen

1. DR3L+


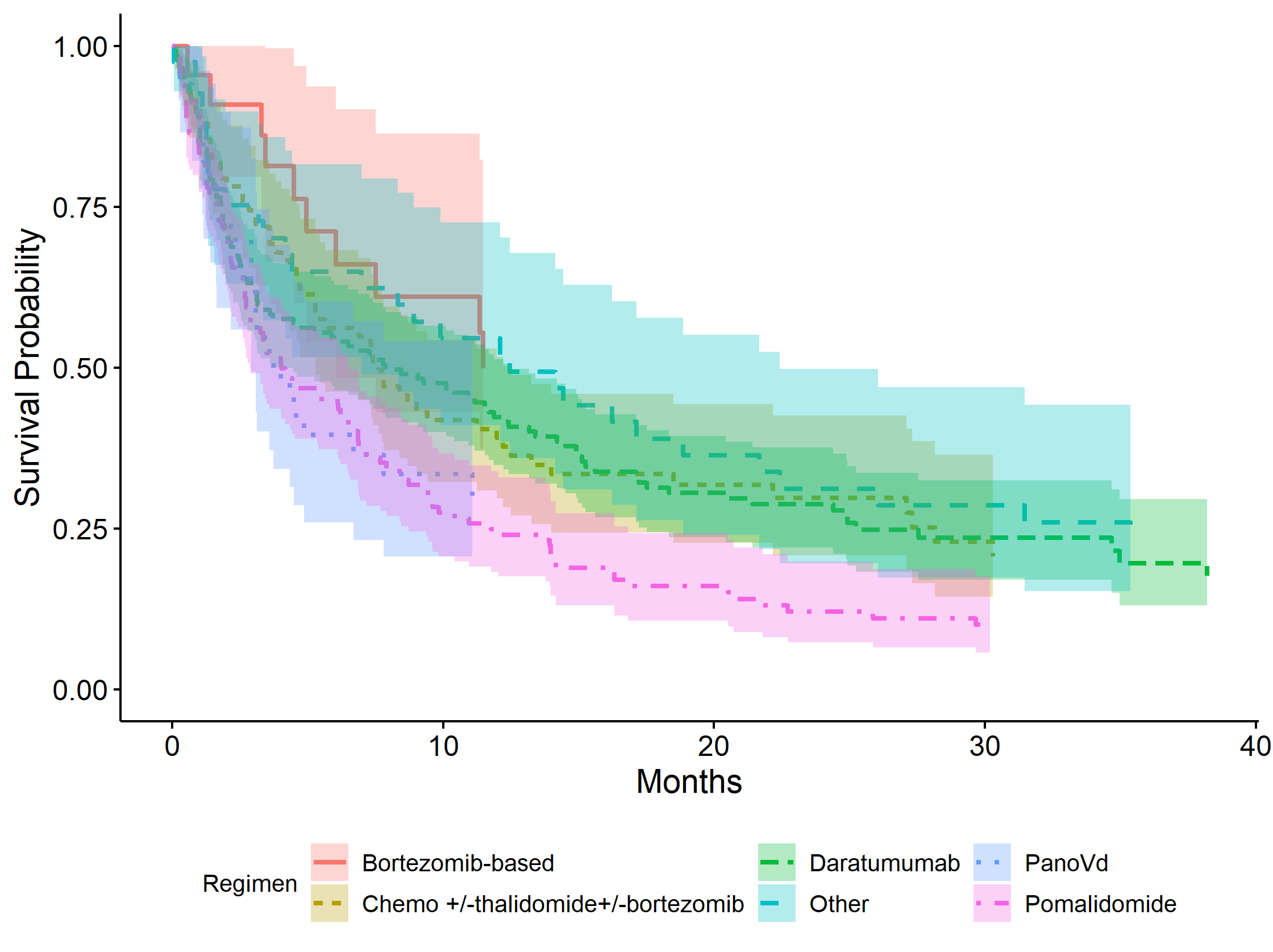


|  | **PomDex** | **PanoVd** | **Daratumumab** | **Chemotherapy ± thalidomide ± bortezomib** | **Bortezomib-based** | **Other** |
| --- | --- | --- | --- | --- | --- | --- |
| Total patients*, N* | 133 | 34 | 145 | 83 | 22 | 41 |
| Number of events, *n* | 119 | 31 | 109 | 65 | 15 | 32 |
| Number censored, *n* | 14 | 3 | 36 | 18 | 7 | 9 |
| Median (95% CI) OS; months | 4.0 (2.9, 6.6) | 3.7 (2.9, 13.3) | 7.9 (4.5, 12.4) | 7.6 (5.3, 12.5) | 11.5 (6.0, N/A) | 12.5 (7.0, 22.4) |
| Months from line start | *N* at risk | | | | | |
| 1 | 109 | 29 | 126 | 75 | 21 | 38 |
| 6 | 56 | 13 | 76 | 43 | 14 | 25 |
| 12 | 29 | – | 56 | 29 | – | 21 |
| 18 | 16 | – | 38 | 20 | – | 15 |
| 24 | 12 | – | 30 | 15 | – | 12 |
| 36 | – | – | 10 | – | – | – |

1. DR4L+


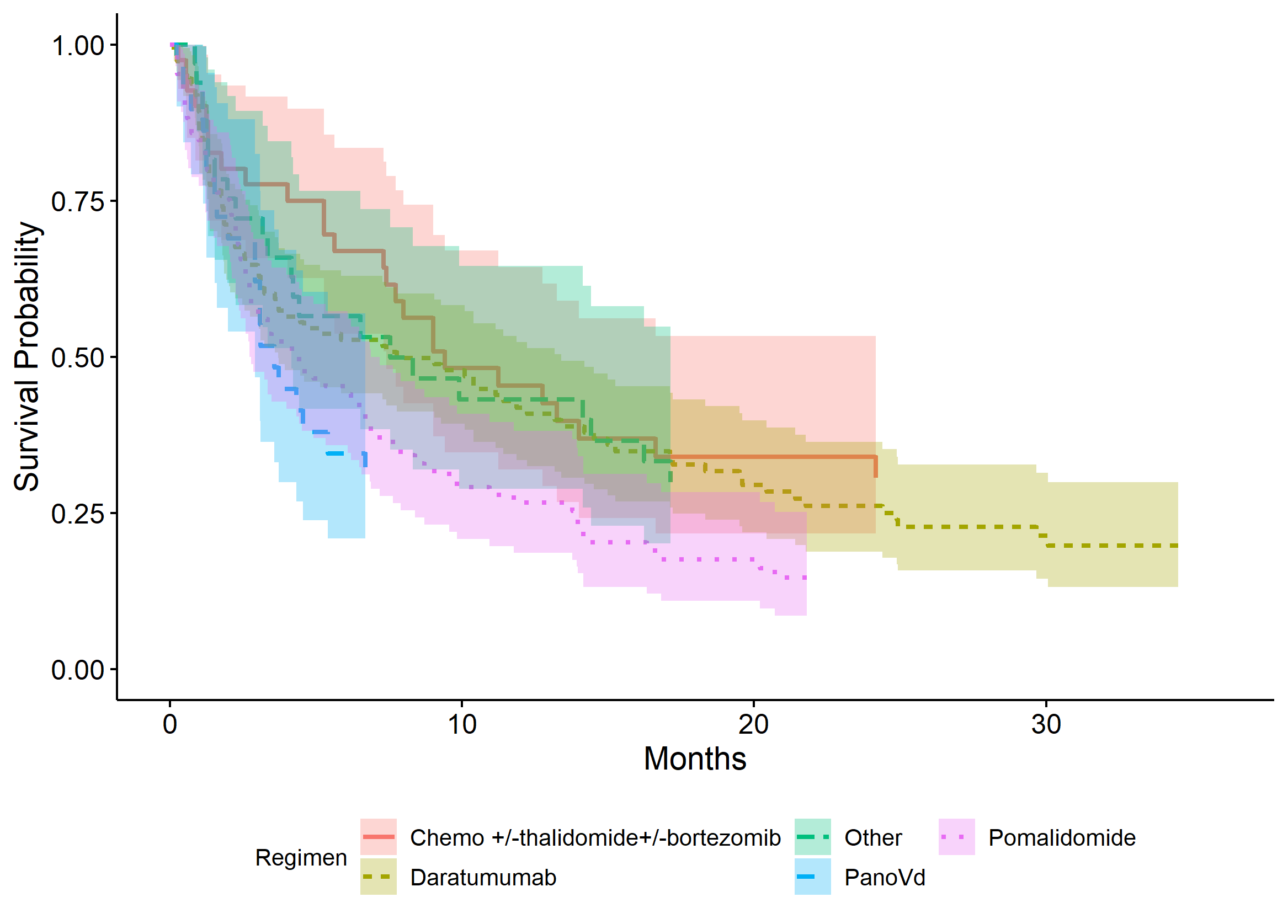


|  | **PomDex** | **PanoVd** | **Daratumumab** | **Chemotherapy ± thalidomide ± bortezomib** | **Other** |
| --- | --- | --- | --- | --- | --- |
| Total patients*, N* | 85 | 29 | 112 | 41 | 33 |
| Number of events, *n* | 79 | 29 | 88 | 32 | 27 |
| Number censored, *n* | 6 | 0 | 24 | 9 | 6 |
| Median (95% CI) OS; months | 4.4 (2.8, 6.9) | 3.6 (2.9, 7.8) | 7.8 (3.7, 13.4) | 9.4 (7.4, 27.1) | 7.5 (4.2, 18.9) |
| Months from line start | *N* at risk | | | | |
| 1 | 71 | 26 | 99 | 36 | 31 |
| 6 | 37 | 10 | 56 | 25 | 17 |
| 12 | 21 | – | 42 | 16 | 13 |
| 18 | 12 | – | 30 | 11 | – |
| 24 | – | – | 23 | 10 | – |

1. TR5L+


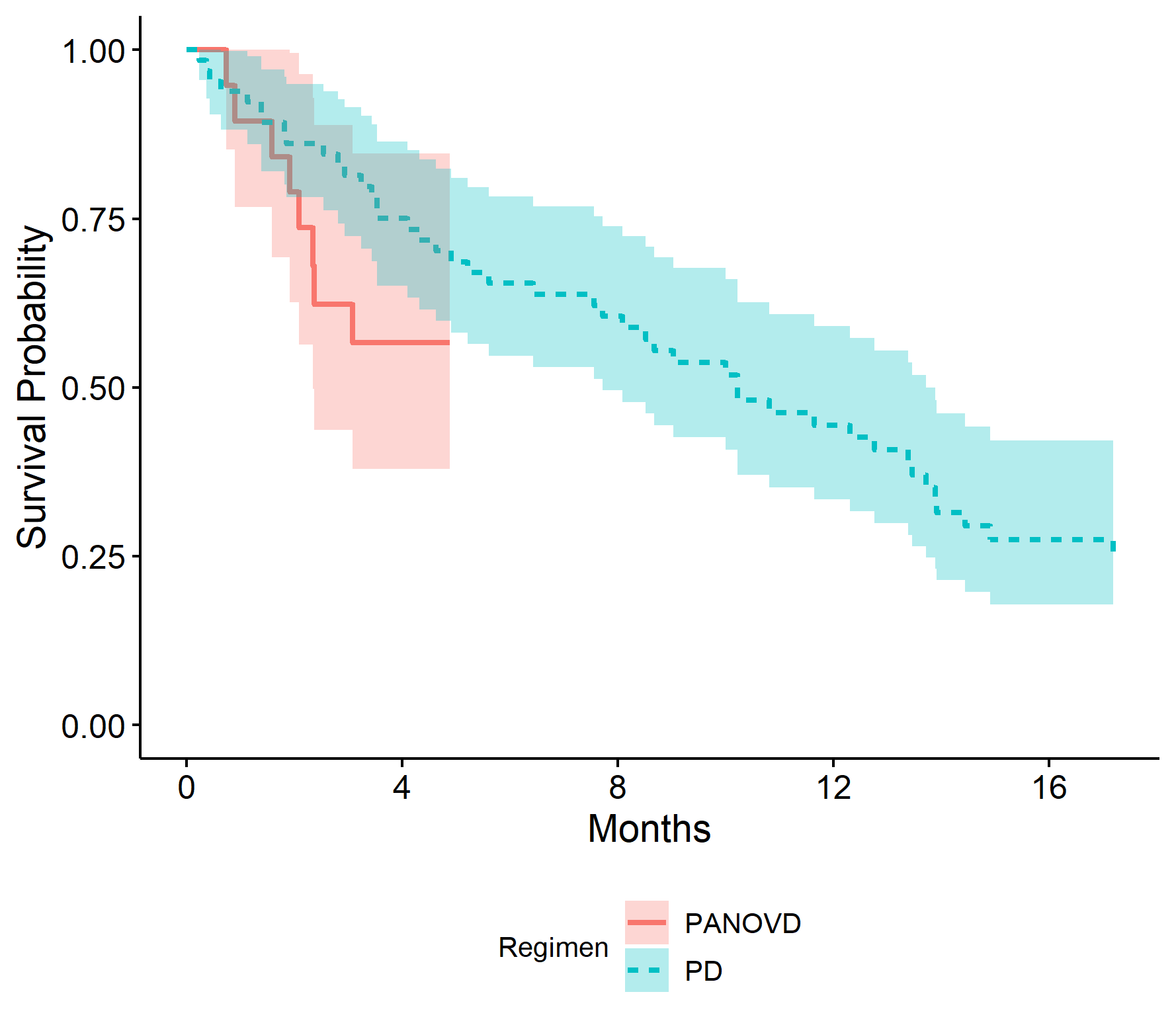


|  | **PomDex** | **PanoVd** |
| --- | --- | --- |
| Total patients*, N* | 65 | 19 |
| Number of events, *n* | 49 | 15 |
| Number censored, *n* | 16 | 4 |
| Median (95% CI) OS; months | 10.2 (7.7, 13.7) | 6.4 (2.3, N/A) |
| Months from line start | *N* at risk | |
| 1 | 61 | 17 |
| 3 | 51 | 11 |
| 6 | 40 | – |
| 9 | 31 | – |
| 12 | 24 | – |
| 15 | 13 | – |

DR3L+ = double class refractory MM commencing a third or higher line of therapy. DR4L+ = double class refractory MM commencing a fourth or higher line of therapy. TR5L+ = triple class refractory MM commencing a fifth or higher line of therapy. Patients at DR3L+, DR4L+ and TR5L+ do not necessarily meet the D7-like and D8-like eligibility criteria. OS is measured in months from start of line of therapy. OS event is death during follow-up. Those alive at follow-up end were censored. Kaplan–Meier analysis is truncated at the point when <10 patients remain in the risk-set.

Abbreviations: 3L, third-line; 4L, fourth-line; 5L, fifth-line; CI, confidence interval; chemo, chemotherapy; D7, DREAMM-7; D8, DREAMM-8; DR, double class refractory; MM, multiple myeloma; N/A, not available; OS, overall survival; PanoVd, panobinostat–bortezomib–dexamethasone; PomDex, pomalidomide–dexamethasone; TR, triple class refractory.

FIGURE S11. TTNTD from initiation of 3L

1. D8-like lenalidomide-exposed


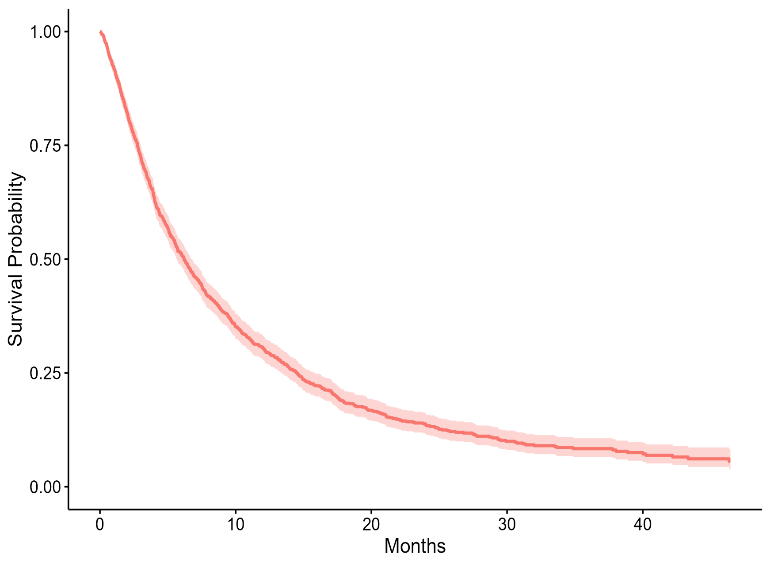


*N* = 1344

Number of events: 1043

Numbers censored: 301

Median (95% CI) TTNTD; months: 6.2 (5.7, 6.8)

| Months | 1 | 6 | 12 | 18 | 24 | 36 |
| --- | --- | --- | --- | --- | --- | --- |
| *N* at risk | 1223 | 616 | 307 | 152 | 92 | 36 |

1. D8-like lenalidomide-refractory


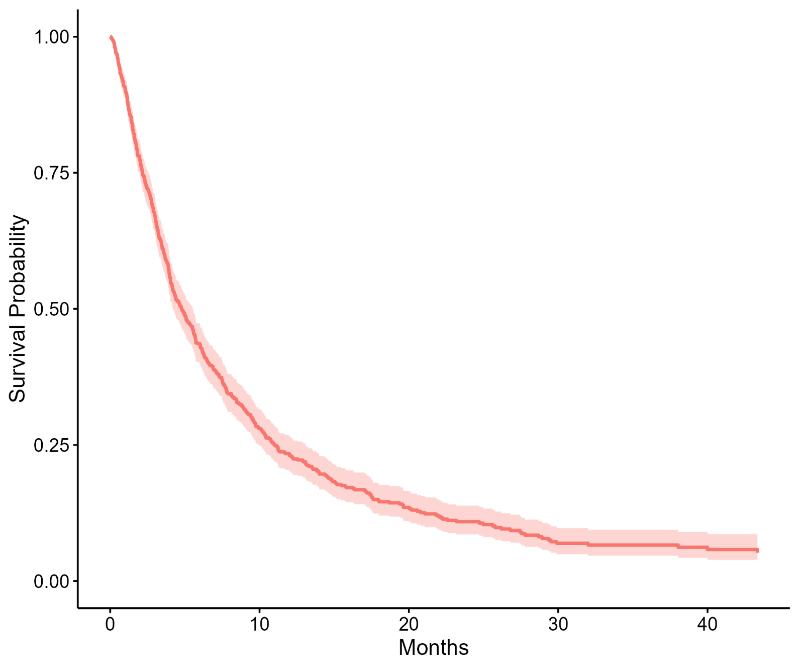


*N* = 776

Number of events: 641

Numbers censored: 135

Median (95% CI) TTNTD; months: 4.8 (4.2, 5.5)

| Months | 1 | 6 | 12 | 18 | 24 | 36 |
| --- | --- | --- | --- | --- | --- | --- |
| *N* at risk | 696 | 306 | 142 | 72 | 44 | 18 |

TTNTD is measured in months from start of line of therapy. TTNTD event is the earliest of a new line start or death during follow-up. Patients remaining on the line of therapy or yet to start a new line and alive at follow-up end were censored. Kaplan–Meier analysis is truncated at the point when <10 patients remain in the risk-set.

Abbreviations: 3L, third-line; CI, confidence interval; D8, DREAMM-8; TTNTD, time to next treatment or death.

FIGURE S12. TTNTD from initiation of 3L by regimen

1. D8-like lenalidomide-exposed


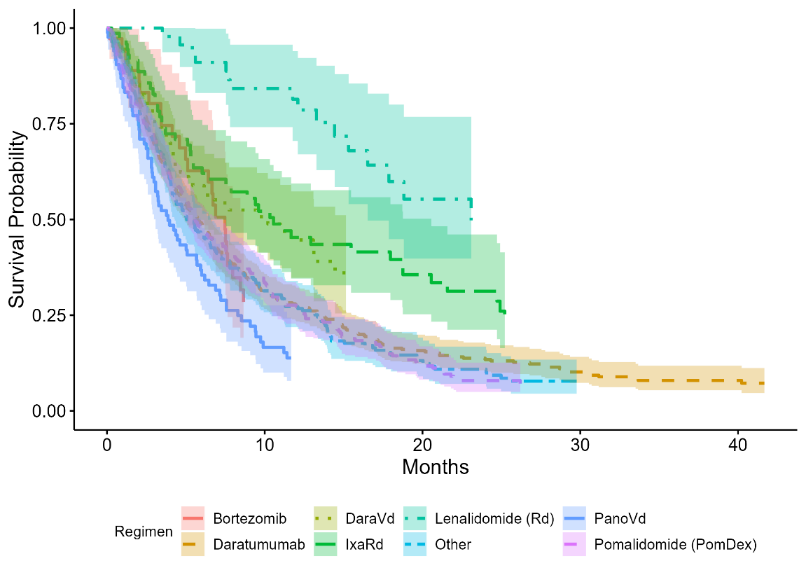


|  | **Bortezomib** | **Daratumumab** | **DaraVd** | **IxaRd** | **Rd** | **PanoVd** | **PomDex** | **Other** |
| --- | --- | --- | --- | --- | --- | --- | --- | --- |
| Total patients*, N* | 36 | 423 | 124 | 74 | 48 | 84 | 304 | 241 |
| Number of events, *n* | 30 | 358 | 56 | 49 | 20 | 76 | 252 | 199 |
| Number censored, *n* | 6 | 65 | 68 | 25 | 28 | 8 | 52 | 42 |
| Median (95% CI) TTNTD; months | 7.5 (5.1, 8.7) | 5.7 (4.9, 6.6) | 10.2 (5.4, 17.5) | 10.6 (6.4, 20.6) | 23.1 (16.5, 0) | 3.9 (3.1, 5.9) | 5.6 (5.1, 7.3) | 5 (4.3, 6.6) |
| Months from line start | *N* at risk | | | | | | | |
| 1 | 34 | 391 | 111 | 69 | 48 | 72 | 270 | 219 |
| 6 | 21 | 196 | 48 | 43 | 40 | 28 | 133 | 101 |
| 12 | – | 104 | 19 | 25 | 29 | – | 62 | 52 |
| 18 | – | 54 | – | 20 | 14 | – | 30 | 24 |
| 24 | – | 38 | – | 13 | – | – | 14 | 14 |
| 36 | – | 15 | – | – | – | – | – | – |

1. D8-like lenalidomide-refractory


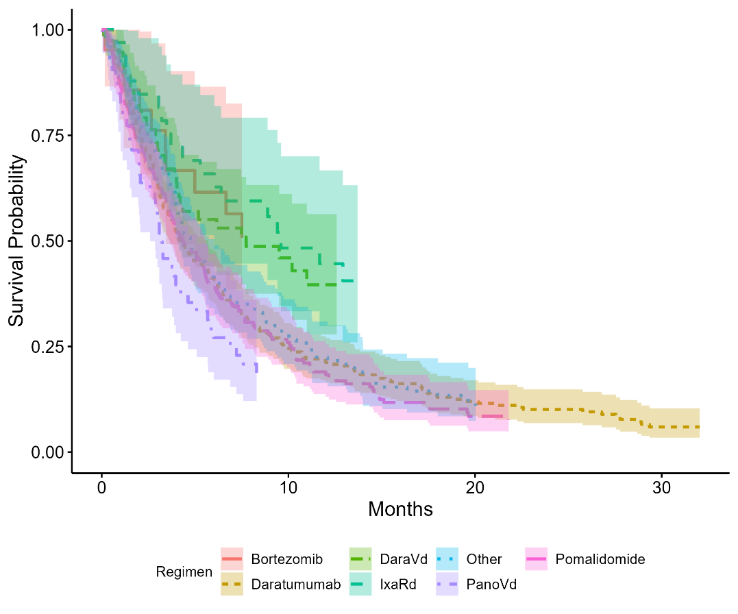


|  | **Bortezomib** | **Daratumumab** | **DaraVd** | **IxaRd** | **PanoVd** | **PomDex** | **Other** |
| --- | --- | --- | --- | --- | --- | --- | --- |
| Total patients*, N* | 21 | 253 | 77 | 34 | 53 | 176 | 149 |
| Number of events, *n* | 16 | 228 | 39 | 22 | 49 | 157 | 126 |
| Number censored, *n* | 5 | 25 | 38 | 12 | 4 | 19 | 23 |
| Median (95% CI) TTNTD; months | 7.6 (3.4, 12.3) | 4.2 (3.6, 5.7) | 7.7 (4.1, N/A) | 9.6 (6.0, N/A) | 3.2 (2.6, 5.1) | 4.4 (3.6, 5.5) | 4.8 (4.0, 6.0) |
| Months from line start | *N* at risk | | | | | | |
| 1 | 19 | 227 | 70 | 32 | 45 | 153 | 137 |
| 6 | 12 | 102 | 27 | 21 | 14 | 64 | 56 |
| 12 | – | 52 | 10 | 11 | – | 27 | 27 |
| 18 | – | 28 | – | – | – | 13 | 14 |
| 24 | – | 20 | – | – | – | – | – |

TTNTD is measured in months from start of line of therapy. TTNTD event is the earliest of a new line start or death during follow-up. Patients remaining on the line of therapy or yet to start a new line and alive at follow-up end were censored. Kaplan–Meier analysis is truncated at the point when <10 patients remain in the risk-set.

Abbreviations: 3L, third-line; CI, confidence interval; D8, DREAMM-8; DaraVd, daratumumab–bortezomib–dexamethasone; IxaRd, ixazomib–lenalidomide–dexamethasone; N/A, not available; PanoVd, panobinostat–bortezomib–dexamethasone; PomDex, pomalidomide–dexamethasone; Rd, lenalidomide–dexamethasone; TTNTD, time to next treatment or death.

FIGURE S13. TTDD from initiation of 3L

1. D8-like lenalidomide-exposed


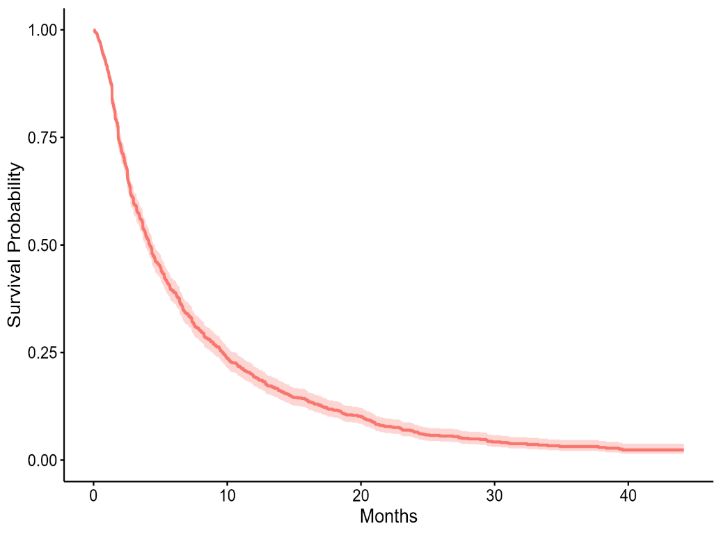


*N* = 1344

Number of events: 1174

Numbers censored: 170

Median (95% CI) TTDD; months: 4.2 (3.9, 4.6)

| Months | 1 | 6 | 12 | 18 | 24 | 36 |
| --- | --- | --- | --- | --- | --- | --- |
| *N* at risk | 1214 | 482 | 207 | 108 | 54 | 17 |

1. D8-like lenalidomide-refractory


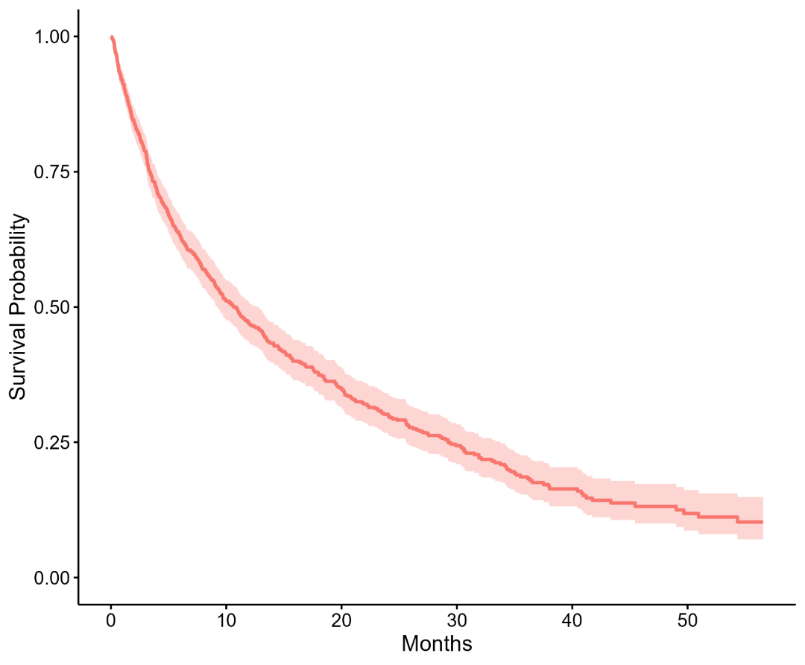


*N* = 776

Number of events: 705

Numbers censored: 71

Median (95% CI) TTDD; months: 3.5 (3.2, 3.9)

| Months | 1 | 6 | 12 | 18 | 24 |
| --- | --- | --- | --- | --- | --- |
| *N* at risk | 692 | 234 | 96 | 53 | 26 |

TTDD is measured in months from start of line of therapy. TTDD event is the earliest of: last administration plus one cycle length; start of a new line minus 1 day; death during follow-up. Patients alive at follow-up end with no subsequent line and last administration date of the line within a cycle length of administrative follow-up end were censored. Kaplan–Meier analysis is truncated at the point when <10 patients remain in the risk-set.

Abbreviations: 3L, third-line; CI, confidence interval; D8, DREAMM-8; TTDD, time to treatment discontinuation or death.

FIGURE S14. TTDD from initiation of 3L by regimen

1. D8-like lenalidomide-exposed


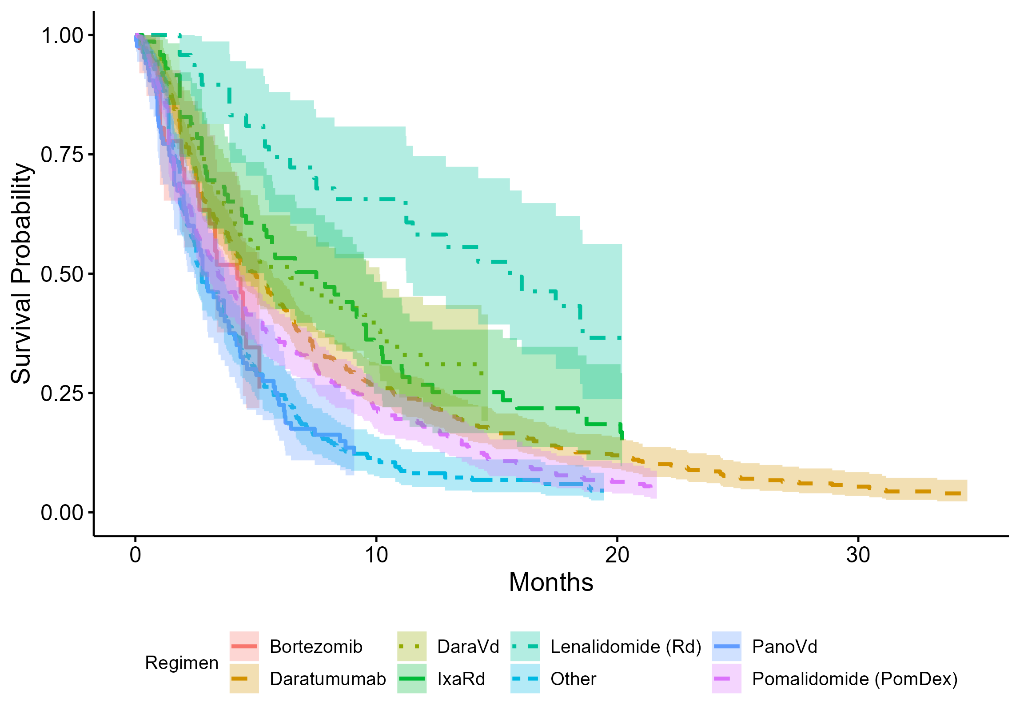


|  | **Bortezomib** | **Daratumumab** | **DaraVd** | **IxaRd** | **Rd** | **PanoVd** | **PomDex** | **Other** |
| --- | --- | --- | --- | --- | --- | --- | --- | --- |
| Total patients*, N* | 36 | 423 | 124 | 74 | 48 | 84 | 304 | 241 |
| Number of events, *n* | 35 | 380 | 68 | 60 | 35 | 80 | 280 | 231 |
| Number censored, *n* | 1 | 43 | 56 | 14 | 13 | 4 | 24 | 10 |
| Median (95% CI) TTDD; months | 4.2 (2.7, 5.1) | 5 (4.1, 5.7) | 6.4 (4.3, 10.1) | 7.5 (5.3, 9.6) | 15.5 (11.2, 23.1) | 3 (2.4, 3.9) | 3.4 (3.0, 4.4) | 2.8 (2.5, 3.7) |
| Months from line start | *N* at risk | | | | | | | |
| 1 | 33 | 391 | 111 | 69 | 48 | 67 | 269 | 217 |
| 6 | – | 174 | 44 | 36 | 34 | 18 | 104 | 57 |
| 12 | – | 84 | 15 | 17 | 23 | – | 44 | 18 |
| 18 | – | 44 | – | 13 | 14 | – | 18 | 13 |
| 24 | – | 28 | – | – | – | – | – | – |

1. D8-like lenalidomide-refractory


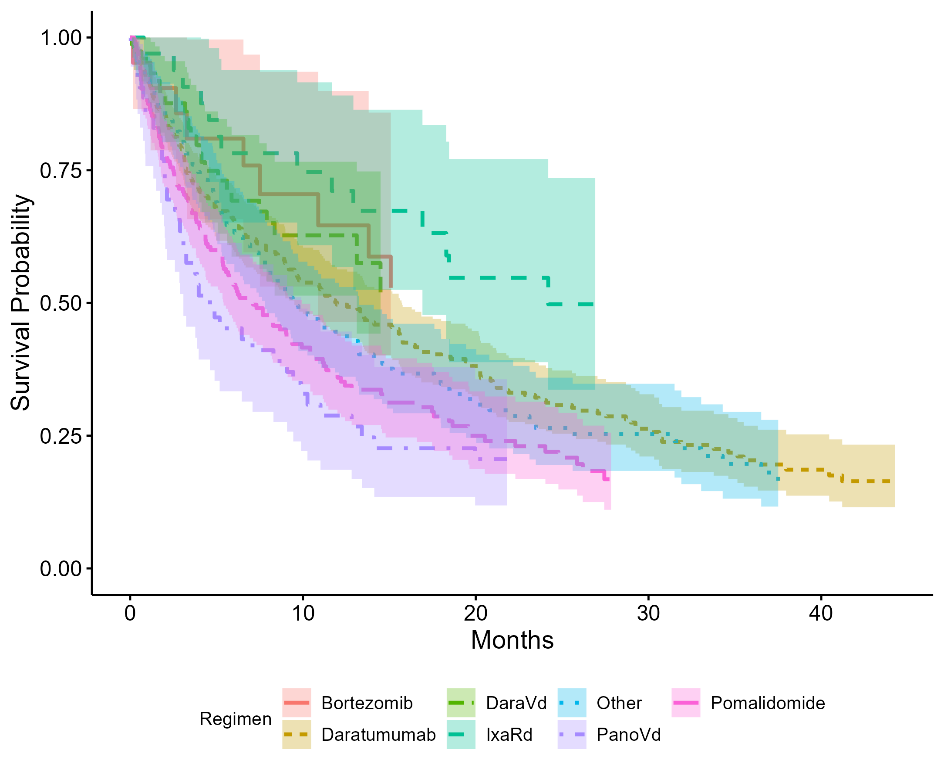


|  | **Bortezomib** | **Daratumumab** | **DaraVd** | **IxaRd** | **PanoVd** | **PomDex** | **Other** |
| --- | --- | --- | --- | --- | --- | --- | --- |
| Total patients*, N* | 21 | 253 | 77 | 34 | 53 | 176 | 149 |
| Number of events, *n* | 21 | 235 | 47 | 29 | 51 | 168 | 145 |
| Number censored, *n* | 0 | 18 | 30 | 5 | 2 | 8 | 4 |
| Median (95% CI) TTDD; months | 3.4 (1.9, 7.2) | 3.9 (3.2, 4.7) | 5.1 (3.9, 9.5) | 5.8 (3.7, 10.3) | 2.7 (2.0, 3.9) | 2.7 (2.3, 3.9) | 2.7 (2.3, 3.7) |
| Months from line start | *N* at risk | | | | | | |
| 1 | 19 | 227 | 70 | 32 | 43 | 152 | 136 |
| 6 | – | 93 | 24 | 15 | – | 50 | 30 |
| 12 | – | 43 | – | – | – | 20 | 11 |
| 18 | – | 24 | – | – | – | 10 | – |
| 24 | – | 15 | – | – | – |  | – |

TTDD is measured in months from start of line of therapy. TTDD event is the earliest of: last administration plus one cycle length; start of a new line minus 1 day; death during follow-up. Patients alive at follow-up end with no subsequent line and last administration date of the line within a cycle length of administrative follow-up end were censored. Kaplan–Meier analysis is truncated at the point when <10 patients remain in the risk-set.

Abbreviations: 3L, third-line; CI, confidence interval; D8, DREAMM-8; DaraVd, daratumumab–bortezomib–dexamethasone; IxaRd, ixazomib–lenalidomide–dexamethasone; PanoVd, panobinostat–bortezomib–dexamethasone; PomDex, pomalidomide–dexamethasone; Rd, lenalidomide–dexamethasone; TTDD, time to treatment discontinuation or death.

FIGURE S15. OS from initiation of 3L

1. D8-like lenalidomide-exposed


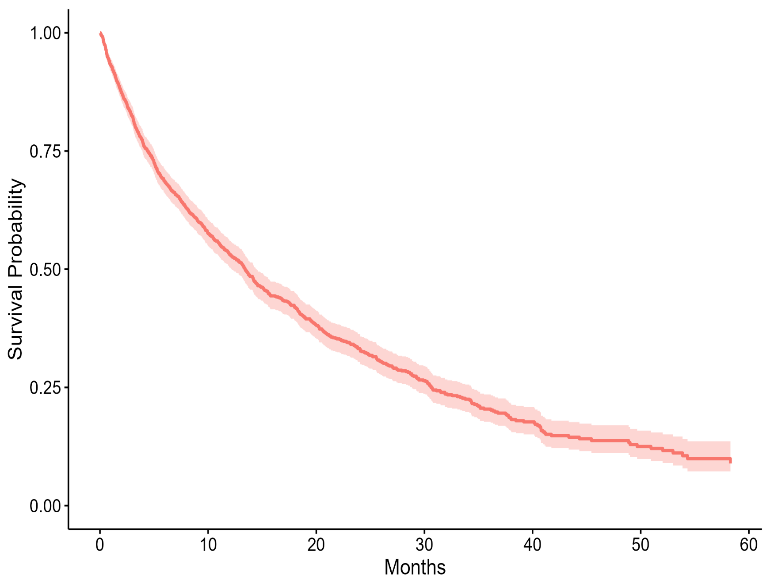


*N* = 1344

Number of events: 876

Numbers censored: 468

Median (95% CI) OS; months: 13.4 (12.2, 14.4)

| Months | 1 | 6 | 12 | 18 | 24 | 36 | 48 |
| --- | --- | --- | --- | --- | --- | --- | --- |
| *N* at risk | 1235 | 832 | 554 | 373 | 258 | 101 | 34 |

1. D8-like lenalidomide-refractory


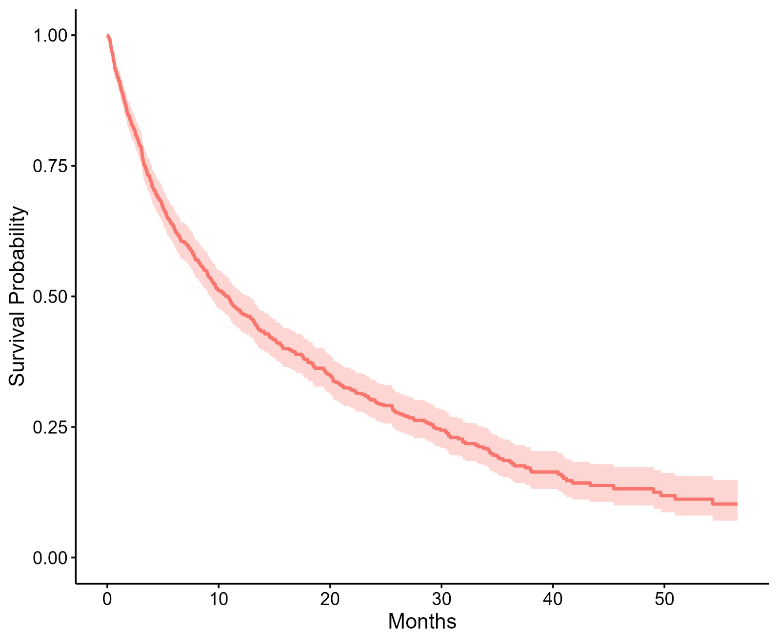


*N* = 776

Number of events: 537

Numbers censored: 239

Median (95% CI) OS; months: 10.7 (9.2, 12.8)

| Months | 1 | 6 | 12 | 18 | 24 | 36 | 48 |
| --- | --- | --- | --- | --- | --- | --- | --- |
| *N* at risk | 705 | 449 | 293 | 200 | 142 | 55 | 20 |

OS is measured in months from start of line of therapy. OS event is death during follow-up. Patients alive at follow-up were censored. Kaplan–Meier analysis is truncated at the point when <10 patients remain in the risk-set.

Abbreviations: 3L, third-line; CI, confidence interval; D8, DREAMM-8; OS, overall survival.

FIGURE S16. OS from initiation of 3L by regimen

1. D8-like lenalidomide-exposed


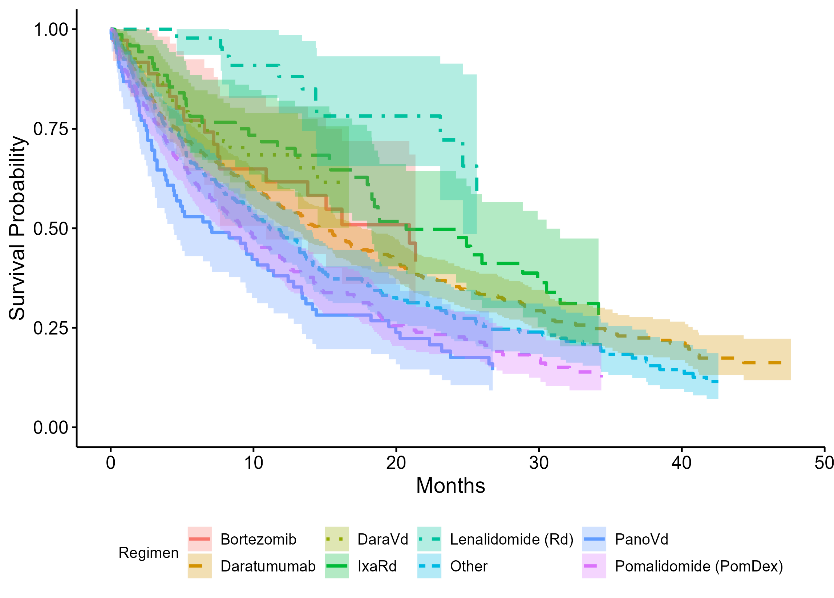


|  | **Bortezomib** | **Daratumumab** | **DaraVd** | **IxaRd** | **Rd** | **PanoVd** | **PomDex** | **Other** |
| --- | --- | --- | --- | --- | --- | --- | --- | --- |
| Total patients*, N* | 36 | 423 | 124 | 74 | 48 | 84 | 304 | 241 |
| Number of events, *n* | 24 | 295 | 33 | 43 | 14 | 72 | 218 | 174 |
| Number censored, *n* | 12 | 128 | 91 | 31 | 34 | 12 | 86 | 67 |
| Median (95% CI) OS; months | 20.9 (10.9, 34.4) | 15.1 (12.6, 17.6) | N/A | 20.7 (18, 30.5) | 43.3 (24.7, 0) | 7.1 (4.4, 12.4) | 9.1 (7.7, 11.5) | 11.2 (9.3, 13.7) |
| Months from line start | *N* at risk | | | | | | | |
| 1 | 35 | 398 | 112 | 69 | 48 | 72 | 272 | 220 |
| 6 | 26 | 283 | 64 | 53 | 43 | 40 | 170 | 146 |
| 12 | 19 | 211 | 28 | 41 | 32 | 27 | 97 | 95 |
| 18 | 13 | 156 | – | 33 | 18 | 20 | 61 | 62 |
| 24 | – | 116 | – | 24 | 12 | 11 | 40 | 42 |
| 36 | – | 47 | – | – | – | – | – | 21 |

1. D8-like lenalidomide-refractory


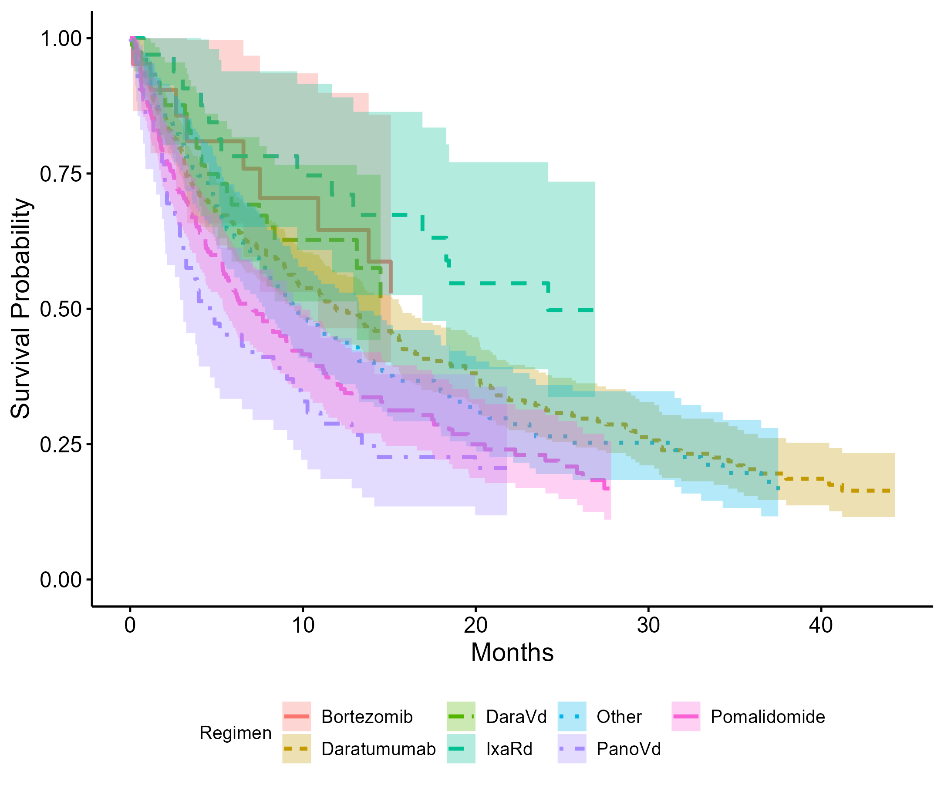


|  | **Bortezomib** | **Daratumumab** | **DaraVd** | **IxaRd** | **PanoVd** | **PomDex** | **Other** |
| --- | --- | --- | --- | --- | --- | --- | --- |
| Total patients*, N* | 21 | 253 | 77 | 34 | 53 | 176 | 149 |
| Number of events, *n* | 13 | 189 | 26 | 18 | 48 | 133 | 106 |
| Number censored, *n* | 8 | 64 | 51 | 16 | 5 | 43 | 43 |
| Median (95% CI) OS; months | 25.5 (10.9*,* N/A) | 12 (9.5, 15.7) | 17.7 (13.1, N/A) | 24.2 (16.9, N/A) | 4.6 (3.1, 9.9) | 7.1 (5.4, 9.9) | 9.7 (7.8, 13.2) |
| Months from line start | *N* at risk | | | | | | |
| 1 | 20 | 232 | 71 | 32 | 45 | 155 | 137 |
| 6 | 16 | 162 | 36 | 25 | 22 | 88 | 89 |
| 12 | 11 | 119 | 17 | 19 | 14 | 50 | 55 |
| 18 | – | 91 | – | 15 | 11 | 32 | 36 |
| 24 | – | 68 | – | 11 | – | 23 | 23 |
| 36 | – | 27 | – | – | – | – | 12 |

OS is measured in months from start of line of therapy. OS event is death during follow-up. Patients alive at follow-up were censored. Kaplan–Meier analysis is truncated at the point when <10 patients remain in the risk-set.

Abbreviations: 3L, third-line; CI, confidence interval; D8, DREAMM-8; DaraVd, daratumumab–bortezomib–dexamethasone; IxaRd, ixazomib–lenalidomide–dexamethasone; N/A, not available; OS, overall survival; PanoVd, panobinostat–bortezomib–dexamethasone; PomDex, pomalidomide–dexamethasone; Rd, lenalidomide–dexamethasone.

TABLE S1. Baseline characteristics of patients treated at 2L by regimen type (excluding DaraVd)

| **D7-like lenalidomide-exposed** | | **Total** | **Bortezomib** | **Daratumumab** | **IxaRd** | **Rd** | **PanoVd** | **PomDex** | **Other** |
| --- | --- | --- | --- | --- | --- | --- | --- | --- | --- |
| Total patients; *N* (%) | | 827 | 79 (9.6) | 126 (15.2) | 20 (2.4) | 38 (4.6) | ^a^ | 72 (8.7) | 183 (22.1) |
| Age at line start; mean (SD) | Years | 73.1 (10.2) | 76.6 (7.3) | 68.9 (10.8) | 69.7 (12.9) | 62.7 (10.9) | – | 75.9 (10.2) | 72.5 (10.3) |
| Age at line start; *N* (%) | 18–44 | 4 (0.5) | – | 1 (0.8) | 1 (5.0) | 1 (2.6) | – | – | – |
|  | 45–64 | 161 (19.5) | 6 (7.6) | 39 (31.0) | 5 (25.0) | 20 (52.6) | – | 14 (19.4) | 44 (24.0) |
|  | 65–74 | 235 (28.4) | 19 (24.1) | 46 (36.5) | 6 (30.0) | 11 (28.9) | – | 9 (12.5) | 50 (27.3) |
|  | 75–84 | 347 (42.0) | 48 (60.8) | 33 (26.2) | 7 (35.0) | 6 (15.8) | – | 36 (50.0) | 67 (36.6) |
|  | 85+ | 80 (9.7) | 6 (7.6) | 7 (5.6) | 1 (5.0) | – | – | 13 (18.1) | 22 (12.0) |
| Gender; *N* (%) | Male | 460 (55.6) | 36 (45.6) | 71 (56.3) | 9 (45.0) | 27 (71.1) | – | 42 (58.3) | 108 (59.0) |
|  | Female | 367 (44.4) | 43 (54.4) | 55 (43.7) | 11 (55.0) | 11 (28.9) | – | 30 (41.7) | 75 (41.0) |
| Ethnicity; *N* (%) | Asian | 28 (3.4) | – | 4 (3.2) | 1 (5.0) | 4 (10.5) | – | 3 (4.2) | 8 (4.4) |
|  | Black | 41 (5.0) | 2 (2.5) | 8 (6.3) | 2 (10.0) | 1 (2.6) | – | 6 (8.3) | 6 (3.3) |
|  | White | 723 (87.4) | 74 (93.7) | 109 (86.5) | 17 (85.0) | 32 (84.2) | – | 61 (84.7) | 158 (86.3) |
|  | Other | 10 (1.2) | – | 1 (0.8) | – | 1 (2.6) | – | 1 (1.4) | 5 (2.7) |
|  | Unknown | 25 (3.0) | 3 (3.8) | 4 (3.2) | – | – | – | 1 (1.4) | 6 (3.3) |
| Stage; *N* (%) | 1 | 62 (7.5) | 2 (2.5) | 3 (2.4) | 1 (5.0) | 8 (21.1) | – | 5 (6.9) | 12 (6.6) |
|  | 2 | 68 (8.2) | 8 (10.1) | 13 (10.3) | 3 (15.0) | 2 (5.3) | – | 1 (1.4) | 14 (7.7) |
|  | 3 | 61 (7.4) | 2 (2.5) | 8 (6.3) | 1 (5.0) | 2 (5.3) | – | 4 (5.6) | 13 (7.1) |
|  | Unstaged | 636 (76.9) | 67 (84.8) | 102 (81.0) | 15 (75.0) | 26 (68.4) | – | 62 (86.1) | 144 (78.7) |
| ECOG performance status; *N* (%) | 0 | 136 (16.4) | 16 (20.3) | 19 (15.1) | 4 (20.0) | 19 (50.0) | – | 9 (12.5) | 39 (21.3) |
|  | 1 | 290 (35.1) | 25 (31.6) | 49 (38.9) | 5 (25.0) | 9 (23.7) | – | 19 (26.4) | 65 (35.5) |
|  | 2 | 132 (16.0) | 14 (17.7) | 17 (13.5) | 4 (20.0) | 2 (5.3) | – | 16 (22.2) | 29 (15.8) |
|  | 3–4 | 24 (2.9) | 2 (2.5) | 3 (2.4) | 1 (5.0) | – | – | 3 (4.2) | 10 (5.5) |
|  | Unknown | 245 (29.6) | 22 (27.8) | 38 (30.2) | 6 (30.0) | 8 (21.1) | – | 25 (34.7) | 40 (21.9) |
| ASCT; *N* (%) | No | 665 (80.4) | 71 (89.9) | 75 (59.5) | 12 (60.0) | 11 (28.9) | – | 66 (91.7) | 150 (82.0) |
|  | Yes | 162 (19.6) | 8 (10.1) | 51 (40.5) | 8 (40.0) | 27 (71.1) | – | 6 (8.3) | 33 (18.0) |
| Lytic bone lesion; *N* (%) | No | 808 (97.7) | 77 (97.5) | 122 (96.8) | 19 (95.0) | 36 (94.7) | – | 69 (95.8) | 181 (98.9) |
|  | Yes | 19 (2.3) | 2 (2.5) | 4 (3.2) | 1 (5.0) | 2 (5.3) | – | 3 (4.2) | 2 (1.1) |
| Extramedullary plasmacytoma; *N* (%) | No | 822 (99.4) | 79 (100.0) | 126 (100.0) | 20 (100.0) | 38 (100.0) | – | 71 (98.6) | 181 (98.9) |
|  | Yes | 5 (0.6) | – | – | – | – | – | 1 (1.4) | 2 (1.1) |
| Prior PI exposure; *N* (%) | No | 741 (89.6) | 77 (97.5) | 81 (64.3) | 18 (90.0) | 33 (86.8) | – | 67 (93.1) | 166 (90.7) |
|  | Yes | 86 (10.4) | 2 (2.5) | 45 (35.7) | 2 (10.0) | 5 (13.2) | – | 5 (6.9) | 17 (9.3) |
| Prior IMID exposure; *N* (%) | No | – | – | – | – | – | – | – | – |
|  | Yes | 827 (100.0) | 79 (100.0) | 126 (100.0) | 20 (100.0) | 38 (100.0) | – | 72 (100.0) | 183 (100.0) |
| Prior lenalidomide exposure; *N* (%) | No | – | – | – | – | – | – | – | – |
|  | Yes | 827 (100.0) | 79 (100.0) | 126 (100.0) | 20 (100.0) | 38 (100.0) | – | 72 (100.0) | 183 (100.0) |
| Lenalidomide-refractory; *N* (%) | No | 466 (56.3) | 45 (57.0) | 70 (55.6) | 14 (70.0) | 38 (100.0) | – | 36 (50.0) | 103 (56.3) |
|  | Yes | 361 (43.7) | 34 (43.0) | 56 (44.4) | 6 (30.0) | – | – | 36 (50.0) | 80 (43.7) |
| Prior anti-CD38 mAb exposure; *N* (%) | No | 827 (100.0) | 79 (100.0) | 126 (100.0) | 20 (100.0) | 38 (100.0) | – | 72 (100.0) | 183 (100.0) |
|  | Yes | – | – | – | – | – | – | – | – |
| Available follow-up from MM diagnosis; median (IQR) | Months | 39.5 (27.9 to 68.9) | 32.0 (24.1 to 50.9) | 70.7 (53.1 to 93.9) | 64.5 (37.2 to 81.4) | 57.6 (31.6 to 87.9) | – | 55.1 (37.6 to 74.1) | 38.3 (27.4 to 61.8) |
| Available follow-up from start of line; median (IQR) | Months | 14.0 (5.2 to 23.9) | 9.5 (3.3 to 22.0) | 18.9 (5.4 to 31.4) | 20.9 (10.8 to 37.7) | 24.7 (16.0 to 50.9) | – | 10.1 (4.8 to 25.6) | 16.8 (7.6 to 24.6) |
| **D7-like lenalidomide-refractory** |  | **Total** | **Bortezomib** | **Daratumumab** | **IxaRd** | **Rd** | **PanoVd** | **PomDex** | **Other** |
| Total patients; *N* (%) | | 361 | 34 (9.4) | 56 (15.5) | ^a^ | 0 | ^a^ | 36 (10.0) | 80 (22.2) |
| Age at line start; mean (SD) | Years | 74.0 (9.4) | 77.2 (6.8) | 70.1 (10.7) | – | – | – | 75.9 (9.6) | 71.9 (10.6) |
| Age at line start; *N* (%) | 18–44 | 2 (0.6) | – | – | – | – | – | – | – |
|  | 45–64 | 58 (16.1) | 3 (8.8) | 16 (28.6) | – | – | – | 7 (19.4) | 21 (26.2) |
|  | 65–74 | 101 (28.0) | 8 (23.5) | 18 (32.1) | – | – | – | 4 (11.1) | 21 (26.2) |
|  | 75–84 | 166 (46.0) | 20 (58.8) | 19 (33.9) | – | – | – | 19 (52.8) | 29 (36.2) |
|  | 85+ | 34 (9.4) | 3 (8.8) | 3 (5.4) | – | – | – | 6 (16.7) | 9 (11.2) |
| Gender; *N* (%) | Male | 201 (55.7) | 18 (52.9) | 29 (51.8) | – | – | – | 21 (58.3) | 49 (61.2) |
|  | Female | 160 (44.3) | 16 (47.1) | 27 (48.2) | – | – | – | 15 (41.7) | 31 (38.8) |
| Ethnicity; *N* (%) | Asian | 12 (3.3) | 0 (0.0) | 2 (3.6) | – | – | – | 2 (5.6) | 4 (5.0) |
|  | Black | 18 (5.0) | 1 (2.9) | 0 (0.0) | – | – | – | 5 (13.9) | 3 (3.8) |
|  | White | 310 (85.9) | 31 (91.2) | 50 (89.3) | – | – | – | 29 (80.6) | 68 (85.0) |
|  | Other | 5 (1.4) | 0 (0.0) | 1 (1.8) | – | – | – | 0 (0.0) | 3 (3.8) |
|  | Unknown | 16 (4.4) | 2 (5.9) | 3 (5.4) | – | – | – | 0 (0.0) | 2 (2.5) |
| Stage; *N* (%) | 1 | 27 (7.5) | 1 (2.9) | 2 (3.6) | – | – | – | 1 (2.8) | 8 (10.0) |
|  | 2 | 34 (9.4) | 3 (8.8) | 9 (16.1) | – | – | – | 1 (2.8) | 5 (6.2) |
|  | 3 | 25 (6.9) | – | 7 (12.5) | – | – | – | 2 (5.6) | 4 (5.0) |
|  | Unstaged | 275 (76.2) | 30 (88.2) | 38 (67.9) | – | – | – | 32 (88.9) | 63 (78.8) |
| ECOG performance status; *N* (%) | 0 | 54 (15.0) | 8 (23.5) | 8 (14.3) | – | – | – | 7 (19.4) | 18 (22.5) |
|  | 1 | 132 (36.6) | 8 (23.5) | 22 (39.3) | – | – | – | 11 (30.6) | 29 (36.2) |
|  | 2 | 63 (17.5) | 6 (17.6) | 8 (14.3) | – | – | – | 6 (16.7) | 12 (15.0) |
|  | 3–4 | 15 (4.2) | 1 (2.9) | 1 (1.8) | – | – | – | 2 (5.6) | 5 (6.2) |
|  | Unknown | 97 (26.9) | 11 (32.4) | 17 (30.4) | – | – | – | 10 (27.8) | 16 (20.0) |
| ASCT; *N* (%) | No | 310 (85.9) | 30 (88.2) | 33 (58.9) | – | – | – | 32 (88.9) | 67 (83.8) |
|  | Yes | 51 (14.1) | 4 (11.8) | 23 (41.1) | – | – | – | 4 (11.1) | 13 (16.2) |
| Lytic bone lesion; *N* (%) | No | 355 (98.3) | 33 (97.1) | 54 (96.4) | – | – | – | 35 (97.2) | 80 (100.0) |
|  | Yes | 6 (1.7) | 1 (2.9) | 2 (3.6) | – | – | – | 1 (2.8) | 0 (0.0) |
| Extramedullary plasmacytoma; *N* (%) | No | 359 (99.4) | 34 (100.0) | 56 (100.0) | – | – | – | 35 (97.2) | 80 (100.0) |
|  | Yes | 2 (0.6) | 0 (0.0) | 0 (0.0) | – | – | – | 1 (2.8) | 0 (0.0) |
| Prior PI exposure; *N* (%) | No | 320 (88.6) | 32 (94.1) | 33 (58.9) | – | – | – | 32 (88.9) | 70 (87.5) |
|  | Yes | 41 (11.4) | 2 (5.9) | 23 (41.1) | – | – | – | 4 (11.1) | 10 (12.5) |
| Prior IMID exposure; *N* (%) | No | – | – | – | – | – | – | – | – |
|  | Yes | 361 (100.0) | 34 (100.0) | 56 (100.0) | – | – | – | 36 (100.0) | 80 (100.0) |
| Prior lenalidomide exposure; *N* (%) | No | – | – | – | – | – | – | – | – |
|  | Yes | 361 (100.0) | 34 (100.0) | 56 (100.0) | – | – | – | 36 (100.0) | 80 (100.0) |
| Len-refractory; *N* (%) | No |  |  |  | – | – | – |  |  |
|  | Yes | 361 (100.0) | 34 (100.0) | 56 (100.0) | – | – | – | 36 (100.0) | 80 (100.0) |
| Prior anti-CD38 mAb exposure; *N* (%) | No | 361 (100.0) | 34 (100.0) | 56 (100.0) | – | – | – | 36 (100.0) | 80 (100.0) |
|  | Yes | – | – | – | – | – | – | – | – |
| Available follow-up from MM diagnosis; median (IQR) | Months | 33.4 (25.4 to 59.0) | 30.5 (26.7 to 37.1) | 69.0 (52.6 to 91.9) | – | – | – | 53.3 (37.6 to 66.3) | 31.2 (25.5 to 42.1) |
| Available follow-up from start of line; median (IQR) | Months | 12.1 (4.5 to 22.5) | 7.4 (2.9 to 14.0) | 18.2 (6.2 to 33.8) | – | – | – | 8.2 (4.5 to 24.4) | 17.9 (7.4 to 23.9) |
| **D8-like lenalidomide-exposed** |  | **Total** | **Bortezomib** | **Daratumumab** | **IxaRd** | **Rd** | **PanoVd** | **PomDex** | **Other** |
| Total patients; *N* (%) |  | 730 (100.0) | 67 (9.2) | 103 (14.1) | ^a^ | 32 (4.4) | ^a^ | 66 (9.0) | 152 (20.8) |
| Age at line start; mean (SD) | Years | 73.1 (10.2) | 76.0 (8.1) | 69.6 (10.9) | – | 62.0 (10.6) | – | 76.7 (9.7) | 71.0 (10.7) |
| Age at line start; *N* (%) | 18–44 | 5 (0.7) | – | 1 (1.0) | – | 1 (3.1) | – | – | 1 (0.7) |
|  | 45–64 | 138 (18.9) | 7 (10.4) | 28 (27.2) | – | 17 (53.1) | – | 11 (16.7) | 43 (28.3) |
|  | 65–74 | 206 (28.2) | 15 (22.4) | 36 (35.0) | – | 10 (31.2) | – | 9 (13.6) | 40 (26.3) |
|  | 75–84 | 311 (42.6) | 39 (58.2) | 32 (31.1) | – | 4 (12.5) | – | 33 (50.0) | 53 (34.9) |
|  | 85+ | 70 (9.6) | 6 (9.0) | 6 (5.8) | – | – | – | 13 (19.7) | 15 (9.9) |
| Gender; *N* (%) | Male | 408 (55.9) | 28 (41.8) | 59 (57.3) | – | 23 (71.9) | – | 39 (59.1) | 91 (59.9) |
|  | Female | 322 (44.1) | 39 (58.2) | 44 (42.7) | – | 9 (28.1) | – | 27 (40.9) | 61 (40.1) |
| Ethnicity; *N* (%) | Asian | 24 (3.3) | – | 3 (2.9) | – | 3 (9.4) | – | 3 (4.5) | 7 (4.6) |
|  | Black | 38 (5.2) | 2 (3.0) | 5 (4.9) | – | 1 (3.1) | – | 6 (9.1) | 7 (4.6) |
|  | White | 633 (86.7) | 62 (92.5) | 88 (85.4) | – | 26 (81.2) | – | 56 (84.8) | 129 (84.9) |
|  | Other | 11 (1.5) | – | 2 (1.9) | – | 1 (3.1) | – | 1 (1.5) | 5 (3.3) |
|  | Unknown | 24 (3.3) | 3 (4.5) | 5 (4.9) | – | 1 (3.1) | – | – | 4 (2.6) |
| Stage; *N* (%) | 1 | 57 (7.8) | 2 (3.0) | 2 (1.9) | – | 7 (21.9) | – | 5 (7.6) | 10 (6.6) |
|  | 2 | 59 (8.1) | 7 (10.4) | 11 (10.7) | – | 2 (6.2) | – | – | 11 (7.2) |
|  | 3 | 54 (7.4) | 2 (3.0) | 8 (7.8) | – | 1 (3.1) | – | 4 (6.1) | 9 (5.9) |
|  | Unstaged | 560 (76.7) | 56 (83.6) | 82 (79.6) | – | 22 (68.8) | – | 57 (86.4) | 122 (80.3) |
| ECOG performance status; *N* (%) | 0 | 124 (17.0) | 16 (23.9) | 17 (16.5) | – | 16 (50.0) | – | 9 (13.6) | 35 (23.0) |
|  | 1 | 255 (34.9) | 24 (35.8) | 42 (40.8) | – | 7 (21.9) | – | 16 (24.2) | 54 (35.5) |
|  | 2 | 106 (14.5) | 9 (13.4) | 13 (12.6) | – | 2 (6.2) | – | 13 (19.7) | 18 (11.8) |
|  | 3–4 | 22 (3.0) | 2 (3.0) | 3 (2.9) | – | – | – | 3 (4.5) | 8 (5.3) |
|  | Unknown | 223 (30.5) | 16 (23.9) | 28 (27.2) | – | 7 (21.9) | – | 25 (37.9) | 37 (24.3) |
| ASCT; *N* (%) | No | 592 (81.1) | 60 (89.6) | 63 (61.2) | – | 9 (28.1) | – | 61 (92.4) | 121 (79.6) |
|  | Yes | 138 (18.9) | 7 (10.4) | 40 (38.8) | – | 23 (71.9) | – | 5 (7.6) | 31 (20.4) |
| Lytic bone lesion; *N* (%) | No | 713 (97.7) | 65 (97.0) | 101 (98.1) | – | 30 (93.8) | – | 64 (97.0) | 149 (98.0) |
|  | Yes | 17 (2.3) | 2 (3.0) | 2 (1.9) | – | 2 (6.2) | – | 2 (3.0) | 3 (2.0) |
| Extramedullary plasmacytoma; *N* (%) | No | 726 (99.5) | 67 (100.0) | 103 (100.0) | – | 32 (100.0) | – | 66 (100.0) | 150 (98.7) |
|  | Yes | 4 (0.5) | – | – | – | – | – | – | 2 (1.3) |
| Prior PI exposure; *N* (%) | No | 669 (91.6) | 65 (97.0) | 71 (68.9) | – | 29 (90.6) | – | 62 (93.9) | 137 (90.1) |
|  | Yes | 61 (8.4) | 2 (3.0) | 32 (31.1) | – | 3 (9.4) | – | 4 (6.1) | 15 (9.9) |
| Prior IMID exposure; *N* (%) | No | – | – | – | – | – | – | – | – |
|  | Yes | 730 (100.0) | 67 (100.0) | 103 (100.0) | – | 32 (100.0) | – | 66 (100.0) | 152 (100.0) |
| Prior anti-CD38 mAb exposure; *N* (%) | No | 730 (100.0) | 67 (100.0) | 103 (100.0) | – | 32 (100.0) | – | 66 (100.0) | 152 (100.0) |
|  | Yes | – | – | – | – | – | – | – | – |
| Prior lenalidomide exposure; *N* (%) | No | – | – | – | – | – | – | – | – |
|  | Yes | 730 (100.0) | 67 (100.0) | 103 (100.0) | – | 32 (100.0) | – | 66 (100.0) | 152 (100.0) |
| Lenalidomide refractory; *N* (%) | No | 359 (49.2) | 33 (49.3) | 47 (45.6) | – | 31 (96.9) | – | 30 (45.5) | 70 (46.1) |
|  | Yes | 371 (50.8) | 34 (50.7) | 56 (54.4) | – | 1 (3.1) | – | 36 (54.5) | 82 (53.9) |
| Available follow-up from MM diagnosis; median (IQR) | Months | 38.9 (27.7 to 67.8) | 32.7 (24.1 to 50.9) | 73.2 (58.1 to 96.9) | – | 38.9 (31.0 to 88.6) | – | 52.3 (34.3 to 69.2) | 39.8 (28.2 to 67.7) |
| Available follow-up from start of line; median (IQR) | Months | 13.4 (5.1 to 22.9) | 8.0 (3.0 to 21.5) | 19.8 (8.1 to 31.1) | – | 18.6 (14.9 to 47.6) | – | 8.9 (4.6 to 23.7) | 16.1 (8.0 to 23.9) |
| **D8-like lenalidomide-refractory** |  | **Total** | **Bortezomib** | **Daratumumab** | **IxaRd** | **Rd** | **PanoVd** | **PomDex** | **Other** |
| Total patients; *N* (%) |  | 371 (100.0) | 34 (9.2) | 56 (15.1) | ^a^ | ^a^ | ^a^ | 36 (9.7) | 82 (22.1) |
| Age at line start; mean (SD) | Years | 73.6 (9.9) | 76.4 (7.8) | 69.9 (11.4) | – | – | – | 76.8 (8.8) | 70.1 (11.6) |
| Age at line start; *N* (%) | 18–44 | 4 (1.1) | – | 1 (1.8) | – | – | – | – | 1 (1.2) |
|  | 45–64 | 60 (16.2) | 4 (11.8) | 15 (26.8) | – | – | – | 5 (13.9) | 25 (30.5) |
|  | 65–74 | 106 (28.6) | 8 (23.5) | 17 (30.4) | – | – | – | 5 (13.9) | 21 (25.6) |
|  | 75–84 | 165 (44.5) | 18 (52.9) | 20 (35.7) | – | – | – | 20 (55.6) | 27 (32.9) |
|  | 85+ | 36 (9.7) | 4 (11.8) | 3 (5.4) | – | – | – | 6 (16.7) | 8 (9.8) |
| Gender; *N* (%) | Male | 204 (55.0) | 17 (50.0) | 27 (48.2) | – | – | – | 22 (61.1) | 49 (59.8) |
|  | Female | 167 (45.0) | 17 (50.0) | 29 (51.8) | – | – | – | 14 (38.9) | 33 (40.2) |
| Ethnicity; *N* (%) | Asian | 12 (3.2) | 0 (0.0) | 2 (3.6) | – | – | – | 2 (5.6) | 4 (4.9) |
|  | Black | 19 (5.1) | 1 (2.9) | 0 (0.0) | – | – | – | 5 (13.9) | 4 (4.9) |
|  | White | 316 (85.2) | 31 (91.2) | 48 (85.7) | – | – | – | 29 (80.6) | 70 (85.4) |
|  | Other | 6 (1.6) | 0 (0.0) | 2 (3.6) | – | – | – | – | 3 (3.7) |
|  | Unknown | 18 (4.9) | 2 (5.9) | 4 (7.1) | – | – | – | – | 1 (1.2) |
| Stage^b^; *N* (%) | 1 | 27 (7.3) | 1 (2.9) | 2 (3.6) | – | – | – | 1 (2.8) | 7 (8.5) |
|  | 2 | 33 (8.9) | 4 (11.8) | 8 (14.3) | – | – | – | – | 5 (6.1) |
|  | 3 | 26 (7.0) | 1 (2.9) | 7 (12.5) | – | – | – | 2 (5.6) | 4 (4.9) |
|  | Unstaged | 285 (76.8) | 28 (82.4) | 39 (69.6) | – | – | – | 33 (91.7) | 66 (80.5) |
| ECOG performance status; *N* (%) | 0 | 58 (15.6) | 10 (29.4) | 8 (14.3) | – | – | – | 7 (19.4) | 20 (24.4) |
|  | 1 | 132 (35.6) | 8 (23.5) | 22 (39.3) | – | – | – | 8 (22.2) | 30 (36.6) |
|  | 2 | 58 (15.6) | 4 (11.8) | 7 (12.5) | – | – | – | 6 (16.7) | 8 (9.8) |
|  | 3–4 | 16 (4.3) | 2 (5.9) | 1 (1.8) | – | – | – | 2 (5.6) | 5 (6.1) |
|  | Unknown | 107 (28.8) | 10 (29.4) | 18 (32.1) | – | – | – | 13 (36.1) | 19 (23.2) |
| ASCT; *N* (%) | No | 316 (85.2) | 30 (88.2) | 33 (58.9) | – | – | – | 33 (91.7) | 65 (79.3) |
|  | Yes | 55 (14.8) | 4 (11.8) | 23 (41.1) | – | – | – | 3 (8.3) | 17 (20.7) |
| Lytic bone lesion; *N* (%) | No | 366 (98.7) | 33 (97.1) | 55 (98.2) | – | – | – | 36 (100.0) | 81 (98.8) |
|  | Yes | 5 (1.3) | 1 (2.9) | 1 (1.8) | – | – | – | – | 1 (1.2) |
| Extramedullary plasmacytoma; *N* (%) | No | 370 (99.7) | 34 (100.0) | 56 (100.0) | – | – | – | 36 (100.0) | 82 (100.0) |
|  | Yes | 1 (0.3) | 0 (0.0) | 0 (0.0) | – | – | – | – | 0 (0.0) |
| Prior PI exposure; *N* (%) | No | 333 (89.8) | 32 (94.1) | 35 (62.5) | – | – | – | 33 (91.7) | 72 (87.8) |
|  | Yes | 38 (10.2) | 2 (5.9) | 21 (37.5) | – | – | – | 3 (8.3) | 10 (12.2) |
| Prior IMID exposure; *N* (%) | No | – | – | – | – | – | – | – | – |
|  | Yes | 371 (100.0) | 34 (100.0) | 56 (100.0) | – | – | – | 36 (100.0) | 82 (100.0) |
| Prior anti-CD38 mAb exposure; *N* (%) | No | 371 (100.0) | 34 (100.0) | 56 (100.0) | – | – | – | 36 (100.0) | 82 (100.0) |
|  | Yes | – | – | – | – | – | – | – | – |
| Prior lenalidomide exposure; *N* (%) | No | – | – | – | – | – | – | – | – |
|  | Yes | 371 (100.0) | 34 (100.0) | 56 (100.0) | – | – | – | 36 (100.0) | 82 (100.0) |
| Lenalidomide refractory; *N* (%) | No | – | – | – | – | – | – | – | – |
|  | Yes | 371 (100.0) | 34 (100.0) | 56 (100.0) | – | – | – | 36 (100.0) | 82 (100.0) |
| Available follow-up from MM diagnosis; median (IQR) | Months | 33.4 (25.4 to 59.5) | 30.8 (26.7 to 38.9) | 69.0 (52.6 to 94.1) | – | – | – | 51.2 (32.8 to 64.7) | 32.7 (27.0 to 56.1) |
| Available follow-up from start of line; median (IQR) | Months | 11.8 (4.5 to 21.7) | 7.4 (2.3 to 15.8) | 18.2 (7.2 to 30.3) | – | – | – | 6.7 (4.1 to 17.9) | 17.4 (8.2 to 24.0) |

Data on patients treated with DaraVd at 2L can be found in the main results. A very small cohort of patients received Kd, KRd and PanoVd – Data and statistics not reported for these group. Presence of ASCT (OPCS-4 code: X334) sourced from National Cancer Registration Dataset, HES APC and OP datasets between date of diagnosis with MM and start of 2L. Presence of lytic bone lesions (ICD-10 codes: M895, M899) sourced from HES APC and OP datasets between date of diagnosis with MM and start of 2L. Extramedullary plasmacytoma (ICD-10 code: C902) sourced from National Cancer Registration Dataset, HES APC and OP datasets between date of diagnosis with MM and start of 2L.

Abbreviations: 2L, second-line; ASCT, autologous stem cell transplant; D7, DREAMM-7; D8, DREAMM-8; DaraVd, daratumumab–bortezomib–dexamethasone; ECOG, Eastern Cooperative Oncology Group; HES APC, hospital episode statistics admitted patient care; ICD-10, International Classification of Diseases, Tenth Revision; IMID, immunomodulatory drugs; IQR, interquartile range; IxaRd, ixazomib–lenalidomide–dexamethasone; Kd, carfilzomib–dexamethasone; KRd, carfilzomib–lenalidomide–dexamethasone; mAb, monoclonal antibody; MM, multiple myeloma; OP, outpatient; OPCS-4, Office of Population Censuses and Surveys Classification of Surgical Operations and Procedures, Fourth Revision; PanoVd, panobinostat–bortezomib–dexamethasone; PI, proteasome inhibitor; PomDex, pomalidomide–dexamethasone; Rd, lenalidomide–dexamethasone; SD, standard deviation.

^a^Small cohort data not reported for this group.
^b^Stage at start of 2L is recorded based on the International Staging System.

TABLE S2. Baseline characteristics of DR3L+, DR4L+ and TR5L+ patients by regimen

| **DR3L+** | | **All DR3L+** | **Bortezomib-based** | **Chemotherapy ± thalidomide ±bortezomib** | **Daratumumab** | **PanoVd** | **Pomalidomide** | **Other** |
| --- | --- | --- | --- | --- | --- | --- | --- | --- |
| Total patients; *N* (%) | | 479 (100) | 22 (4.6) | 83 (17.3) | 145 (30.3) | 34 (7.1) | 133 (27.8) | 41 (8.6) |
| Age at line start; mean (SD) | Years | 68.4 (11.1) | 66.1 (10.9) | 64.0 (11.8) | 70.3 (10.1) | 67.8 (12.1) | 71.0 (11.1) | 64.7 (9.5) |
| Age at line start; *N* (%) | 18–44 | 9 (1.9) | 1 (4.5) | 3 (3.6) | 1 (0.7) | 1 (2.9) | 2 (1.5) | 0 (0) |
|  | 45–64 | 155 (32.4) | 7 (31.8) | 39 (47.0) | 39 (26.9) | 13 (38.2) | 33 (24.8) | 20 (48.8) |
|  | 65–74 | 155 (32.4) | 10 (45.5) | 22 (26.5) | 48 (33.1) | 11 (32.4) | 39 (29.3) | 14 (34.1) |
|  | 75–84 | 142 (29.6) | 3 (13.6) | 16 (19.3) | 52 (35.9) | 7 (20.6) | 52 (39.1) | 7 (17.1) |
|  | 85+ | 18 (3.8) | 1 (4.5) | 3 (3.6) | 5 (3.4) | 2 (5.9) | 7 (5.3) | 0 (0) |
| Gender; *N* (%) | Male | 287 (59.9) | 12 (54.5) | 53 (63.9) | 87 (60.0) | 14 (41.2) | 83 (62.4) | 23 (56.1) |
|  | Female | 192 (40.1) | 10 (45.5) | 30 (36.1) | 58 (40.0) | 20 (58.8) | 50 (37.6) | 18 (43.9) |
| Ethnicity; *N* (%) | Asian | 16 (3.3) | 1 (4.5) | 2 (2.4) | 6 (4.1) | 0 (0) | 1 (0.8) | 3 (7.3) |
|  | Black | 20 (4.2) | 1 (4.5) | 5 (6.0) | 4 (2.8) | 2 (5.9) | 5 (3.8) | 3 (7.3) |
|  | White | 416 (86.8) | 17 (77.3) | 70 (84.3) | 130 (89.7) | 28 (82.4) | 119 (89.5) | 34 (82.9) |
|  | Other | 12 (2.5) | 2 (9.1) | 2 (2.4) | 2 (1.4) | 1 (2.9) | 4 (3.0) | 1 (2.4) |
|  | Unknown | 15 (3.1) | 1 (4.5) | 4 (4.8) | 3 (2.1) | 3 (8.8) | 4 (3.0) | 0 (0) |
| Stage; *N* (%) | 1 | 35 (7.3) | 2 (9.1) | 9 (10.8) | 7 (4.8) | 4 (11.8) | 9 (6.8) | 2 (4.9) |
|  | 2 | 42 (8.8) | 2 (9.1) | 4 (4.8) | 16 (11.0) | 1 (2.9) | 14 (10.5) | 3 (7.3) |
|  | 3 | 61 (12.7) | 3 (13.6) | 9 (10.8) | 23 (15.9) | 5 (14.7) | 16 (12.0) | 4 (9.8) |
|  | Unstaged | 341 (71.2) | 15 (68.2) | 61 (73.5) | 99 (68.3) | 24 (70.6) | 94 (70.7) | 32 (78) |
| ECOG performance status; *N* (%) | 0 | 89 (18.6) | 2 (9.1) | 22 (26.5) | 27 (18.6) | 6 (17.6) | 17 (12.8) | 7 (17.1) |
|  | 1 | 164 (34.2) | 11 (50.0) | 25 (30.1) | 48 (33.1) | 17 (50.0) | 46 (34.6) | 13 (31.7) |
|  | 2 | 111 (23.2) | 2 (9.1) | 15 (18.1) | 38 (26.2) | 4 (11.8) | 36 (27.1) | 12 (29.3) |
|  | 3–4 | 18 (3.8) | 2 (9.1) | 5 (6) | 3 (2.1) | 0 (0) | 7 (5.3) | 0 (0) |
|  | Unknown | 97 (20.3) | 5 (22.7) | 16 (19.3) | 29 (20.0) | 7 (20.6) | 27 (20.3) | 9 (22.0) |
| ASCT; *N* (%) | No | 347 (72.4) | 17 (77.3) | 66 (79.5) | 108 (74.5) | 18 (52.9) | 104 (78.2) | 23 (56.1) |
|  | Yes | 132 (27.6) | 5 (22.7) | 17 (20.5) | 37 (25.5) | 16 (47.1) | 29 (21.8) | 18 (43.9) |
| Lytic bone lesion; *N* (%) | No | 464 (96.9) | 22 (100.0) | 81 (97.6) | 140 (96.6) | 34 (100.0) | 129 (97.0) | 38 (92.7) |
|  | Yes | 15 (3.1) | 0 (0) | 2 (2.4) | 5 (3.4) | 0 (0) | 4 (3.0) | 3 (7.3) |
| Extramedullary plasmacytoma; *N* (%) | No | 468 (97.7) | 22 (100.0) | 82 (98.8) | 142 (97.9) | 33 (97.1) | 128 (96.2) | 41 (100.0) |
|  | Yes | 11 (2.3) | 0 (0) | 1 (1.2) | 3 (2.1) | 1 (2.9) | 5 (3.8) | 0 (0) |
| Prior PI exposure; *N* (%) | Yes | 479 (100.0) | 22 (100.0) | 83 (100.0) | 145 (100.0) | 34 (100.0) | 133 (100.0) | 41 (100.0) |
| Prior IMID exposure; *N* (%) | Yes | 479 (100.0) | 22 (100.0) | 83 (100.0) | 145 (100.0) | 34 (100.0) | 133 (100.0) | 41 (100.0) |
| Prior Len exposure; *N* (%) | No | 479 (100.0) | 22 (100.0) | 83 (100.0) | 145 (100.0) | 34 (100.0) | 133 (100.0) | 41 (100.0) |
|  | Yes | 475 (99.2) | 22 (100.0) | 80 (96.4) | 145 (100.0) | 34 (100.0) | 132 (99.2) | 41 (100.0) |
| Prior anti-CD38 mAb exposure; *N* (%) | Yes | 4 (0.8) | 0 (0) | 3 (3.6) | 0 (0) | 0 (0) | 1 (0.8) | 0 (0) |
| Available follow-up from MM diagnosis; median IQR | Months | 38.6 (24.0 to 58.1) | 40.5 (28.9 to 65.8) | 40.5 (28.9 to 65.8) | 45.4 (27.3 to 67.2) | 38.5 (26.6 to 54.2) | 36.9 (23.0 to 52.9) | 45.9 (27.9 to 71.6) |
| **DR4L+** | | **All DR4L+** | **Chemotherapy ± thalidomide ± bortezomib** | **Daratumumab** | **PanoVd** | **Pomalidomide** | **Other** | |
| Total patients; *N* (%) | | 326 (100.0) | 41 (12.6) | 112 (34.4) | 29 (8.9) | 85 (26.1) | 33 (10.1) | |
| Age at line start; mean (SD) | Years | 67.9 (11.1) | 66.0 (11.7) | 70.0 (10.2) | 65.0 (12.7) | 70.0 (10.7) | 62.9 (10.5) | |
| Age at line start; *N* (%) | 18–44 | 6 (1.8) | 1 (2.4) | 1 (0.9) | 1 (3.4) | 1 (1.2) | 0 (0) | |
|  | 45–64 | 114 (35.0) | 16 (39.0) | 29 (25.9) | 15 (51.7) | 26 (30.6) | 19 (57.6) | |
|  | 65–74 | 105 (32.2) | 13 (31.7) | 40 (35.7) | 7 (24.1) | 26 (30.6) | 8 (24.2) | |
|  | 75–84 | 90 (27.6) | 8 (19.5) | 40 (35.7) | 5 (17.2) | 27 (31.8) | 0 (0) | |
|  | 85+ | 11 (3.4) | 3 (7.3) | 2 (1.8) | 1 (3.4) | 5 (5.9) | 0 (0) | |
| Gender; *N* (%) | Male | 192 (58.9) | 27 (65.9) | 66 (58.9) | 10 (34.5) | 52 (61.2) | 21 (63.6) | |
|  | Female | 134 (41.1) | 14 (34.1) | 46 (41.1) | 19 (65.5) | 33 (38.8) | 12 (36.4) | |
| Ethnicity; *N* (%) | Asian | 9 (2.8) | 0 (0) | 4 (3.6) | 0 (0) | 1 (1.2) | 2 (6.1) | |
|  | Black | 17 (5.2) | 1 (2.4) | 4 (3.6) | 2 (6.9) | 5 (5.9) | 4 (12.1) | |
|  | White | 288 (88.3) | 37 (90.2) | 100 (89.3) | 26 (89.7) | 77 (90.6) | 27 (81.8) | |
|  | Other | 5 (1.5) | 2 (4.9) | 0 (0) | 0 (0) | 1 (1.2) | 0 (0) | |
|  | Unknown | 7 (2.1) | 1 (2.4) | 4 (3.6) | 1 (3.4) | 1 (1.2) | 0 (0) | |
| Stage; *N* (%) | 1 | 22 (6.7) | 3 (7.3) | 7 (6.2) | 3 (10.3) | 5 (5.9) | 2 (6.1) | |
|  | 2 | 25 (7.7) | 0 (0) | 10 (8.9) | 2 (6.9) | 9 (10.6) | 2 (6.1) | |
|  | 3 | 43 (13.2) | 3 (7.3) | 19 (17.0) | 3 (10.3) | 10 (11.8) | 5 (15.2) | |
|  | Unstaged | 236 (72.4) | 35 (85.4) | 76 (67.9) | 21 (72.4) | 61 (71.8) | 24 (72.7) | |
| ECOG performance status; *N* (%) | 0 | 57 (17.5) | 11 (26.8) | 19 (17.0) | 3 (10.3) | 10 (11.8) | 6 (18.2) | |
|  | 1 | 117 (35.9) | 9 (22.0) | 44 (39.3) | 17 (58.6) | 31 (36.5) | 10 (30.3) | |
|  | 2 | 74 (22.7) | 9 (22.0) | 29 (25.9) | 1 (3.4) | 22 (25.9) | 8 (24.2) | |
|  | 3–4 | 9 (2.8) | 2 (4.9) | 1 (0.9) | 0 (0) | 5 (5.9) | 0 (0) | |
|  | Unknown | 69 (21.2) | 10 (24.4) | 19 (17.0) | 8 (27.6) | 17 (20) | 9 (27.3) | |
| ASCT; *N* (%) | No | 220 (67.5) | 31 (75.6) | 81 (72.3) | 14 (48.3) | 60 (70.6) | 18 (54.5) | |
|  | Yes | 106 (32.5) | 10 (24.4) | 31 (27.7) | 15 (51.7) | 25 (29.4) | 15 (45.5) | |
| Lytic bone lesion; *N* (%) | No | 317 (97.2) | 41 (100.0) | 108 (96.4) | 29 (100.0) | 83 (97.6) | 31 (93.9) | |
|  | Yes | 9 (2.8) | 0 (0) | 4 (3.6) | 0 (0) | 2 (2.4) | 2 (6.1) | |
| Extramedullary plasmacytoma; *N* (%) | No | 318 (97.5) | 41 (100.0) | 110 (98.2) | 28 (96.6) | 81 (95.3) | 33 (100.0) | |
|  | Yes | 8 (2.5) | 0 (0) | 2 (1.8) | 1 (3.4) | 4 (4.7) | 0 (0) | |
| Prior PI exposure; *N* (%) | Yes | 326 (100.0) | 41 (100.0) | 112 (100.0) | 29 (100.0) | 85 (100.0) | 33 (100.0) | |
| Prior IMID exposure; *N* (%) | Yes | 326 (100.0) | 41 (100.0) | 112 (100.0) | 29 (100.0) | 85 (100.0) | 33 (100.0) | |
| Prior Len exposure; *N* (%) | No | 326 (100.0) | 41 (100.0) | 112 (100.0) | 29 (100.0) | 85 (100.0) | 33 (100.0) | |
|  | Yes | 324 (99.4) | 39 (95.1) | 112 (100.0) | 29 (100.0) | 85 (100.0) | 33 (100.0) | |
| Prior anti-CD38 exposure; *N* (%) | Yes | 2 (0.6) | 2 (4.9) | 0 (0) | 0 (0) | 0 (0) | 0 (0) | |
| Available follow-up from MM diagnosis; median IQR | Months | 41.1 (26.7 to 62.3) | 41.2 (26.6 to 53.6) | 44.5 (27.9 to 67.0) | 38.4 (26.4 to 48.9) | 40.6 (27.0 to 53.4) | 40.5 (27.7 to 64.8) | |
| **TR5L+** | | **All TR5L+** | | **PanoVd** | | **PomDex** | | |
| Total patients; *N* (%) |  | 136 (100.0) | | 19 (100.0) | | 65 (47.8) | | |
| Age at line start; mean (SD) | Years | 67.2 (10.1) | | 66.4 (10.0) | | 69.2 (9.8) | | |
| Age at line start; *N* (%) | 18–44 | 1 (0.7) | | 0 (0) | | 1 (1.5) | | |
|  | 45–64 | 51 (37.5) | | 7 (36.8) | | 15 (23.1) | | |
|  | 65–74 | 50 (36.8) | | 7 (36.8) | | 29 (44.6) | | |
|  | 75–84 | 31 (22.8) | | 5 (26.3) | | 19 (29.2) | | |
|  | 85+ | 3 (2.2) | | 0 (0) | | 1 (1.5) | | |
| Gender; *N* (%) | Male | 88 (64.7) | | 13 (68.4) | | 38 (58.5) | | |
|  | Female | 48 (35.3) | | 6 (31.6) | | 27 (41.5) | | |
| Ethnicity; *N* (%) | Asian | 3 (2.2) | | 1 (5.3) | | 1 (1.5) | | |
|  | Black | 6 (4.4) | | 1 (5.3) | | 2 (3.1) | | |
|  | White | 121 (89) | | 15 (78.9) | | 60 (92.3) | | |
|  | Other | 2 (1.5) | | 1 (5.3) | | 0 (0) | | |
|  | Unknown | 4 (2.9) | | 1 (5.3) | | 2 (3.1) | | |
| Stage^a^; *N* (%) | 1 | 10 (7.4) | | 3 (15.8) | | 3 (4.6) | | |
|  | 2 | 11 (8.1) | | 4 (21.1) | | 5 (7.7) | | |
|  | 3 | 20 (14.7) | | 0 (0) | | 15 (23.1) | | |
|  | Unstaged | 95 (69.9) | | 12 (63.2) | | 42 (64.6) | | |
| ECOG performance status; *N* (%) | 0 | 23 (16.9) | | 4 (21.1) | | 9 (13.8) | | |
|  | 1 | 52 (38.2) | | 8 (42.1) | | 31 (47.7) | | |
|  | 2 | 27 (19.9) | | 4 (21.1) | | 12 (18.2) | | |
|  | 3–4 | 7 (5.1) | | 1 (5.3) | | 2 (3.1) | | |
|  | Unknown | 27 (19.9) | | 2 (10.5) | | 11 (16.9) | | |
| ASCT; *N* (%) | No | 75 (55.1) | | 11 (57.9) | | 43 (66.2) | | |
|  | Yes | 61 (44.9) | | 8 (42.1) | | 22 (33.8) | | |
| Lytic bone lesion; *N* (%) | No | 130 (95.6) | | 18 (94.7) | | 61 (93.8) | | |
|  | Yes | 6 (4.4) | | 1 (5.3) | | 4 (6.2) | | |
| Extramedullary plasmacytoma; *N* (%) | No | 130 (95.6) | | 19 (100.0) | | 62 (95.4) | | |
|  | Yes | 6 (4.4) | | 0 (0) | | 3 (4.6) | | |
| Prior PI exposure; *N* (%) | Yes | 136 (100.0) | | 19 (100.0) | | 65 (100.0) | | |
| Prior IMID exposure; *N* (%) | Yes | 136 (100.0) | | 19 (100.0) | | 65 (100.0) | | |
| Prior Len exposure; *N* (%) | No | 13 (9.6) | | 0 (0) | | 4 (6.2) | | |
|  | Yes | 123 (90.4) | | 19 (100.0) | | 61 (93.8) | | |
| Prior anti-CD38 exposure; *N* (%) | Yes | 136 (100.0) | | 19 (100.0) | | 65 (100.0) | | |
| Available follow-up from MM diagnosis; median IQR | Months | 62.8 (42.9 to 78.9) | | 60.8 (45.4 to 73.4) | | 56.1 (40.8 to 79.1) | | |

DR3L+ = double class refractory MM commencing a third or higher line of therapy. DR4L+ = double class refractory MM commencing a fourth or higher line of therapy. TR5L+ = triple class refractory MM commencing a fifth or higher line of therapy. Patients at DR3L+, DR4L+ and TR5L+ do not necessarily meet the D7-like and D8-like eligibility criteria. Presence of ASCT (OPCS-4 code: X334) sourced from National Cancer Registration Dataset, HES APC and OP datasets between date of diagnosis with MM and start of the index line of therapy. Presence of lytic bone lesions (ICD-10 codes: M895, M899) sourced from HES APC and OP datasets between date of diagnosis with MM and start of the index line of therapy. Extramedullary plasmacytoma (ICD-10 code: C902) sourced from National Cancer Registration Dataset, HES APC and OP datasets between date of diagnosis with MM and start of the index line of therapy. IMID: lenalidomide, pomalidomide, thalidomide; PI: bortezomib, carfilzomib, ixazomib; Anti-CD38 mAb: daratumumab, isatuximab.

Abbreviations: 3L, third-line; 4L, fourth-line; 5L, fifth-line; ASCT, autologous stem cell transplant; D7, DREAMM-7; D8, DREAMM-8; DR, double class refractory; ECOG, Eastern Cooperative Oncology Group; HES APC, hospital episode statistics admitted patient care; ICD-10, International Classification of Diseases, Tenth Revision; IMID, immunomodulatory drugs; IQR, interquartile range; mAb, monoclonal antibody; MM, multiple myeloma; OP, outpatient; OPCS-4, Office of Population Censuses and Surveys Classification of Surgical Operations and Procedures, Fourth Revision; PanoVd, panobinostat–bortezomib–dexamethasone; PI, proteasome inhibitor; PomDex, pomalidomide–dexamethasone; SD, standard deviation; TR, triple class refractory.

^a^Stage at start of index line of therapy is recorded based on the International Staging System.

TABLE S3. Baseline characteristics of patients treated at 2L and excluded due to CDF embargo

|  | | **D7-like cohort** | | **D8-like cohort** | |
| --- | --- | --- | --- | --- | --- |
|  | | Len-exposed | Len-refractory | Len-exposed | Len-refractory |
| Total patients; *N* (%) | | 56 (100.0) | 21 (100.0) | 50 (100.0) | 23 (100.0) |
| Age at line start; mean (SD) | Years | 67.6 (11.3) | 66.7 (10.8) | 67.4 (11.3) | 66.0 (10.7) |
| Age at line start; *N* (%) | 18–44 | – | – | – | – |
|  | 45–64 | 19 (33.9) | ^a^ | 17 (34.0) | ^a^ |
|  | 65–74 | 20 (35.7) | 10 (47.6) | 19 (38.0) | 12 (52.2) |
|  | 75–84 | 15 (26.8) | ^a^ | 12 (24.0) | ^a^ |
|  | 85+ | 2 (3.6) | – | 2 (4.0) | – |
| Gender; *N* (%) | Male | 34 (60.7) | 13 (61.9) | 31 (62.0) | 16 (69.6) |
|  | Female | 22 (39.3) | 8 (38.1) | 19 (38.0) | 7 (30.4) |
| Ethnicity; *N* (%) | Asian | 6 (10.7) | ^a^ | 5 (10.0) | ^a^ |
|  | Black | 4 (7.1) | ^a^ | 3 (6.0) | ^a^ |
|  | White | 45 (80.4) | 17 (81.0) | 41 (82.0) | 19 (82.6) |
|  | Other | 1 (1.8) | – | 1 (2.0) | – |
|  | Unknown | 0 (0.0) | – | – | – |
| Stage^b^; *N* (%) | 1 | 3 (5.4) | ^a^ | 3 (6.0) | ^a^ |
|  | 2 | 3 (5.4) | ^a^ | 3 (6.0) | ^a^ |
|  | 3 | 4 (7.1) | ^a^ | 4 (8.0) | ^a^ |
|  | Unstaged | 46 (82.1) | 16 (76.2) | 40 (80.0) | 17 (73.9) |
| ECOG performance status; *N* (%) | 0 | 6 (10.7) | ^a^ | 6 (12.0) | ^a^ |
|  | 1 | 19 (33.9) | ^a^ | 19 (38.0) | 6 (26.1) |
|  | 2 | 7 (12.5) | ^a^ | 5 (10.0) | ^a^ |
|  | 3–4 | – | ^a^ | – | – |
|  | Unknown | 24 (42.9) | 10 (47.6) | 20 (40.0) | 11 (47.8) |
| ASCT; *N* (%) | No | 28 (50.0) | 11 (52.4) | 26 (52.0) | 12 (52.2) |
|  | Yes | 28 (50.0) | 10 (47.6) | 24 (48.0) | 11 (47.8) |
| Lytic bone lesion; *N* (%) | No | 53 (94.6) | ^a^ | 47 (94.0) | ^a^ |
|  | Yes | 3 (5.4) | ^a^ | 3 (6.0) | ^a^ |
| Extramedullary plasmacytoma; *N* (%) | No | 56 (100.0) | 21 (100.0) | 50 (100.0) | 20 (100.0) |
|  | Yes | – | – | – | – |
| Prior PI exposure; *N* (%) | No | 39 (69.6) | 14 (66.7) | 36 (72.0) | 16 (69.6) |
|  | Yes | 17 (30.4) | 7 (33.3) | 14 (28.0) | 7 (30.4) |
| Prior IMID exposure; *N* (%) | No | – | – | – | – |
|  | Yes | 56 (100.0) | 21 (100.0) | 50 (100.0) | 23 (100.0) |
| Prior lenalidomide exposure; *N* (%) | No | – | – | – | – |
|  | Yes | 56 (100.0) | 21 (100.0) | 50 (100.0) | 23 (100.0) |
| Lenalidomide-refractory; *N* (%) | No | 35 (62.5) | – | 27 (54.0) | – |
|  | Yes | 21 (37.5) | 21 (100.0) | 23 (46.0) | 23 (100.0) |
| Prior anti-CD38 mAb exposure; *N* (%) | No | 56 (100.0) | 21 (100.0) | 50 (100.0) | 23 (100.0) |
|  | Yes | – | – | – | – |

Presence of ASCT (OPCS-4 code: X334) sourced from National Cancer Registration Dataset, HES APC and OP datasets between date of diagnosis with MM and start of 2L. Presence of lytic bone lesions (ICD-10 codes: M895, M899) sourced from HES APC and OP datasets between date of diagnosis with MM and start of 2L. Extramedullary plasmacytoma (ICD-10 code: C902) sourced from National Cancer Registration Dataset, HES APC and OP datasets between date of diagnosis with MM and start of 2L. IMID: lenalidomide, pomalidomide, thalidomide; PI: bortezomib, carfilzomib, ixazomib; Anti-CD38 mAb: daratumumab, isatuximab.

Abbreviations: 2L, second-line; ASCT, autologous stem cell transplant; CDF, Cancer Drugs Fund; D7, DREAMM-7; D8, DREAMM-8; ECOG, Eastern Cooperative Oncology Group; HES APC, hospital episode statistics admitted patient care; ICD-10, International Classification of Diseases, Tenth Revision; IMID, immunomodulatory drugs; Len, lenalidomide; mAb, monoclonal antibody; MM, multiple myeloma; OP, outpatient; OPCS-4, Office of Population Censuses and Surveys Classification of Surgical Operations and Procedures, Fourth Revision; PI, proteasome inhibitor; SD, standard deviation.

^a^Small cohort–data not reported for this group.
^b^Stage at start of 2L is recorded based on the International Staging System.

TABLE S4. Baseline characteristics of DR3L+, DR4L+ and TR5L+ patients excluded due to CDF embargo

|  | | **DR3L+** | **DR4L+** | **TR5L+** |
| --- | --- | --- | --- | --- |
| Total patients; *N* (%) | | 638 (100.0) | 434 (100.0) | 266 (100.0) |
| Age at line start; mean (SD) | Years | 69.2 (9.7) | 69.0 (10.1) | 67.2 (10.0) |
| Age at line start; *N* (%) | 18–44 | 4 (0.6) | 3 (0.7) | 4 (1.5) |
|  | 45–64 | 193 (30.3) | 142 (32.7) | 96 (36.1) |
|  | 65–74 | 235 (36.8) | 148 (34.1) | 96 (36.1) |
|  | 75–84 | 181 (28.4) | 122 (28.1) | 66 (24.8) |
|  | 85+ | 25 (3.9) | 19 (4.4) | 4 (1.5) |
| Gender; *N* (%) | Male | 376 (58.9) | 255 (58.8) | 145 (54.5) |
|  | Female | 262 (41.1) | 179 (41.2) | 121 (45.5) |
| Ethnicity; *N* (%) | Asian | 17 (2.7) | 12 (2.8) | 8 (3.0) |
|  | Black | 38 (6.0) | 29 (6.7) | 25 (9.4) |
|  | White | 560 (87.8) | 379 (87.3) | 222 (83.5) |
|  | Other | 17 (2.7) | 11 (2.5) | 9 (3.4) |
|  | Unknown | 6 (0.9) | 3 (0.7) | 2 (0.8) |
| Stage^a^; *N* (%) | 1 | 53 (8.3) | 36 (8.3) | 23 (8.6) |
|  | 2 | 85 (13.3) | 54 (12.4) | 38 (14.3) |
|  | 3 | 87 (13.6) | 61 (14.1) | 36 (13.5) |
|  | Unstaged | 413 (64.7) | 283 (65.2) | 169 (63.5) |
| ECOG performance status; *N* (%) | 0 | 112 (17.6) | 87 (20.0) | 44 (16.5) |
|  | 1 | 243 (38.1) | 148 (34.1) | 100 (37.6) |
|  | 2 | 108 (16.9) | 78 (18.0) | 53 (19.9) |
|  | 3–4 | 17 (2.7) | 14 (3.2) | 3 (1.1) |
|  | Unknown | 158 (24.8) | 107 (24.7) | 66 (24.8) |
| ASCT; *N* (%) | No | 370 (58.0) | 256 (59.0) | 124 (46.6) |
|  | Yes | 268 (42.0) | 178 (41.0) | 142 (53.4) |
| Lytic bone lesion; *N* (%) | No | 615 (96.4) | 418 (96.3) | 253 (95.1) |
|  | Yes | 23 (3.6) | 16 (3.7) | 13 (4.9) |
| Extramedullary plasmacytoma; *N* (%) | No | 628 (98.4) | 426 (98.2) | 261 (98.1) |
|  | Yes | 10 (1.6) | 8 (1.8) | 5 (1.9) |
| Prior PI exposure; *N* (%) | Yes | 638 (100.0) | 434 (100.0) | 266 (100.0) |
| Prior IMID exposure; *N* (%) | Yes | 638 (100.0) | 434 (100.0) | 266 (100.0) |
| Prior lenalidomide exposure; *N* (%) | No | 638 (100.0) | 434 (100.0) | 21 (7.9) |
|  | Yes | 622 (97.5) | 413 (95.2) | 245 (92.1) |
| Prior anti-CD38 exposure; *N* (%) | Yes | 16 (2.5) | 21 (4.8) | 266 (100.0) |

DR3L+ = double class refractory MM commencing a third or higher line of therapy. DR4L+ = double class refractory MM commencing a fourth or higher line of therapy. TR5L+ = triple class refractory MM commencing a fifth or higher line of therapy. Patients at DR3L+, DR4L+ and TR5L+ do not necessarily meet the D7-like and D8-like eligibility criteria. Presence of ASCT (OPCS-4 code: X334) sourced from National Cancer Registration Dataset, HES APC and OP datasets between date of diagnosis with MM and start of the index line of therapy. Presence of lytic bone lesions (ICD-10 codes: M895, M899) sourced from HES APC and OP datasets between date of diagnosis with MM and start of the index line of therapy. Extramedullary plasmacytoma (ICD-10 code: C902) sourced from National Cancer Registration Dataset, HES APC and OP datasets between date of diagnosis with MM and start of the index line of therapy. IMID: lenalidomide, pomalidomide, thalidomide; PI: bortezomib, carfilzomib, ixazomib; anti-CD38 mAb: daratumumab, isatuximab.

Abbreviations: 3L, third-line; 4L, fourth-line; 5L, fifth-line; ASCT, autologous stem cell transplant; CDF, Cancer Drugs Fund; D7, DREAMM-7; D8, DREAMM-8; DR, double class refractory; ECOG, Eastern Cooperative Oncology Group; HES APC, hospital episode statistics admitted patient care; ICD-10, International Classification of Diseases, Tenth Revision; IMID, immunomodulatory drugs; mAb, monoclonal antibody; MM, multiple myeloma; OP, outpatient; OPCS-4, Office of Population Censuses and Surveys Classification of Surgical Operations and Procedures, Fourth Revision; PI, proteasome inhibitor; SD, standard deviation; TR, triple class refractory.

^a^Stage at start of index line of therapy is recorded based on the International Staging System.

TABLE S5. Prior treatments of patients treated at 2L by regimen and drug class

| **D7-like lenalidomide-exposed** | | **Total** | **Bortezomib** | **Daratumumab** | **DaraVd** | **IxaRd** | **Rd** | **PomDex** | **Other** |
| --- | --- | --- | --- | --- | --- | --- | --- | --- | --- |
| Total number of patient lines | | 827 | 79 (9.6) | 126 (15.2) | 282 (34.1) | 20 (2.4) | 38 (4.6) | 72 (8.7) | 183 (22.1) |
| 1L regimen | Lenalidomide | 584 (70.6) | 57 (72.2) | 62 (49.2) | 251 (89.0) | 13 (65.0) | – | 54 (75.0) | 130 (71.0) |
|  | Cyclophosphamide– lenalidomide | 152 (18.4) | 20 (25.3) | 18 (14.3) | 22 (7.8) | 4 (20.0) | 32 (84.2) | 13 (18.1) | 36 (19.7) |
|  | Ixazomib–lenalidomide | 70 (8.5) | 2 (2.5) | 44 (34.9) | 2 (0.7) | – | 1 (2.6) | 5 (6.9) | 14 (7.7) |
|  | Bortezomib–lenalidomide | 6 (0.7) | – | – | 1 (0.4) | 1 (5.0) | 3 (7.9) | – | 1 (0.5) |
|  | Bortezomib–cyclophosphamide–lenalidomide | 5 (0.6) | – | – | 3 (1.1) | 1 (5.0) | 1 (2.6) | – | – |
|  | Cyclophosphamide–lenalidomide–melphalan | 1 (0.1) | – | – | – | – | – | – | – |
|  | Cyclophosphamide–ixazomib–lenalidomide | 1 (0.1) | – | 1 (0.8) | – | – | – | – | – |
|  | Cyclophosphamide–lenalidomide–pomalidomide | 1 (0.1) | – | 1 (0.8) | – | – | – | – | – |
|  | Bortezomib–cyclophosphamide–lenalidomide–thalidomide | 1 (0.1) | – | – | 1 (0.4) | – | – | – | – |
|  | Bortezomib–lenalidomide–thalidomide | 1 (0.1) | – | – | 1 (0.4) | – | – | – | – |
|  | Lenalidomide–rituximab | 1 (0.1) | – | – | 1 (0.4) | – | – | – | – |
|  | Cyclophosphamide–lenalidomide–vincristine | 1 (0.1) | – | – | – | 1 (5.0) | – | – | – |
|  | Lenalidomide–melphalan–rituximab | 1 (0.1) | – | – | – | – | 1 (2.6) | – | – |
|  | Bortezomib–cyclophosphamide–lenalidomide–melphalan | 1 (0.1) | – | – | – | – | – | – | 1 (0.5) |
|  | Carfilzomib–cyclophosphamide–lenalidomide | 1 (0.1) | – | – | – | – | – | – | 1 (0.5) |
| 1L drug class | IMID | 584 (70.6) | 57 (72.2) | 62 (49.2) | 251 (89.0) | 13 (65.0) | – | 54 (75.0) | 130 (71.0) |
|  | Chemotherapies–IMID | 155 (18.7) | 20 (25.3) | 19 (15.1) | 22 (7.8) | 5 (25.0) | 32 (84.2) | 13 (18.1) | 36 (19.7) |
|  | IMID–PI | 77 (9.3) | 2 (2.5) | 44 (34.9) | 4 (1.4) | 1 (5.0) | 4 (10.5) | 5 (6.9) | 15 (8.2) |
|  | Chemotherapies–IMID–PI | 9 (1.1) | – | 1 (0.8) | 4 (1.4) | 1 (5.0) | 1 (2.6) | – | 2 (1.1) |
|  | IMID–mAb | 1 (0.1) | – | – | 1 (0.4) | – | – | – | – |
|  | Chemotherapies–IMID–mAb | 1 (0.1) | – | – | – | – | 1 (2.6) | – | – |
| **D7-like lenalidomide-refractory** | | **Total** | **Bortezomib** | **Daratumumab** | **DaraVd** | **IxaRd** | **Rd** | **PomDex** | **Other** |
| Total number of patient lines | | 361 | 34 (9.4) | 56 (15.5) | 143 (39.6) | – | – | 36 (10.0) | 80 (22.2) |
| 1L regimen | Lenalidomide | 281 (77.8) | 25 (73.5) | 22 (39.3) | 134 (93.7) | – | – | 27 (75.0) | 64 (80.0) |
|  | Ixazomib–lenalidomide | 40 (11.1) | 2 (5.9) | 22 (39.3) | 1 (0.7) | – | – | 4 (11.1) | 10 (12.5) |
|  | Cyclophosphamide–lenalidomide | 38 (10.5) | 7 (20.6) | 11 (19.6) | 7 (4.9) | – | – | 5 (13.9) | 6 (7.5) |
|  | Cyclophosphamide–ixazomib–lenalidomide | 1 (0.3) | – | 1 (1.8) | – | – | – | – | – |
|  | Lenalidomide–rituximab | 1 (0.3) | – | – | 1 (0.7) | – | – | – | – |
| 1L drug class | IMID | 281 (77.8) | 25 (73.5) | 22 (39.3) | 134 (93.7) | – | – | 27 (75.0) | 64 (80.0) |
|  | IMID–PI | 40 (11.1) | 2 (5.9) | 22 (39.3) | 1 (0.7) | – | – | 4 (11.1) | 10 (12.5) |
|  | Chemotherapies–IMID | 38 (10.5) | 7 (20.6) | 11 (19.6) | 7 (4.9) | – | – | 5 (13.9) | 6 (7.5) |
|  | Chemotherapies–IMID–PI | 1 (0.3) | – | 1 (1.8) | – | – | – | – | – |
|  | IMID–mAb | 1 (0.3) | – | – | 1 (0.7) | – | – | – | – |
| **D8-like lenalidomide-exposed** | | **Total** | **Bortezomib** | **Daratumumab** | **DaraVd** | **IxaRd** | **Rd** | **PomDex** | **Other** |
| Total number of patient lines | | 730 (100.0) | 67 (9.2) | 103 (14.1) | 269 (36.8) | – | 32 (4.4) | 66 (9.0) | 152 (20.8) |
| 1L regimen | Lenalidomide | 543 (74.4) | 48 (71.6) | 56 (54.4) | 247 (91.8) | – | – | 50 (75.8) | 111 (73.0) |
|  | Cyclophosphamide–lenalidomide | 122 (16.7) | 17 (25.4) | 15 (14.6) | 18 (6.7) | – | 27 (84.4) | 12 (18.2) | 26 (17.1) |
|  | Ixazomib–lenalidomide | 56 (7.7) | 2 (3.0) | 31 (30.1) | 2 (0.7) | – | 1 (3.1) | 4 (6.1) | 14 (9.2) |
|  | Bortezomib–lenalidomide | 2 (0.3) | – | – | – | – | 2 (6.2) | – | – |
|  | Bortezomib–cyclophosphamide–lenalidomide–thalidomide | 1 (0.1) | – | – | 1 (0.4) | – | – | – | – |
|  | Carfilzomib–cyclophosphamide–lenalidomide | 1 (0.1) | – | – | – | – | – | – | 1 (0.7) |
|  | Cyclophosphamide–ixazomib–lenalidomide | 1 (0.1) | – | 1 (1.0) | – | – | – | – | – |
|  | Cyclophosphamide–lenalidomide–melphalan | 1 (0.1) | – | – | – | – | – | – | – |
|  | Cyclophosphamide–lenalidomide–thalidomide | 1 (0.1) | – | – | – | – | 1 (3.1) | – | – |
|  | Lenalidomide–melphalan–rituximab | 1 (0.1) | – | – | – | – | 1 (3.1) | – | – |
|  | Lenalidomide–rituximab | 1 (0.1) | – | – | 1 (0.4) | – | – | – | – |
| 1L drug class | IMID | 543 (74.4) | 48 (71.6) | 56 (54.4) | 247 (91.8) | – | – | 50 (75.8) | 111 (73.0) |
|  | Chemotherapies–IMID | 124 (16.9) | 17 (25.4) | 15 (14.6) | 18 (6.7) | – | 28 (87.5) | 12 (18.2) | 26 (17.1) |
|  | IMID–PI | 58 (7.9) | 2 (3.0) | 31 (30.1) | 2 (0.7) | – | 3 (9.4) | 4 (6.1) | 14 (9.2) |
|  | Chemotherapies–IMID–PI | 3 (0.4) | – | 1 (1.0) | 1 (0.4) | – | – | – | 1 (0.7) |
|  | Chemotherapies–IMID–mAb | 1 (0.1) | – | – | – | – | 1 (3.1) | – | – |
|  | IMID–mAb | 1 (0.1) | – | – | 1 (0.4) | – | – | – | – |
| **D8-like lenalidomide-refractory** | | **Total** | **Bortezomib** | **Daratumumab** | **DaraVd** | **IxaRd** | **Rd** | **PomDex** | **Other** |
| Total number of patient lines | | 371 (100.0) | 34 (9.2) | 56 (15.1) | 148 (39.9) | – | – | 36 (9.7) | 82 (22.1) |
| 1L regimen | Lenalidomide | 290 (78.2) | 24 (70.6) | 24 (42.9) | 140 (94.6) | – | – | 28 (77.8) | 64 (78.0) |
|  | Cyclophosphamide–lenalidomide | 41 (11.1) | 8 (23.5) | 11 (19.6) | 6 (4.1) | – | – | 5 (13.9) | 8 (9.8) |
|  | Ixazomib–lenalidomide | 37 (10.0) | 2 (5.9) | 20 (35.7) | 1 (0.7) | – | – | 3 (8.3) | 10 (12.2) |
|  | Cyclophosphamide–ixazomib–lenalidomide | 1 (0.3) | – | 1 (1.8) | – | – | – | – | – |
|  | Cyclophosphamide–lenalidomide–thalidomide | 1 (0.3) | – | – | – | – | – | – | – |
|  | Lenalidomide–rituximab | 1 (0.3) | – | – | 1 (0.7) | – | – | – | – |
| 1L drug class | IMID | 290 (78.2) | 24 (70.6) | 24 (42.9) | 141 (95.3) | – | – | 28 (77.8) | 64 (78.0) |
|  | Chemotherapies–IMID | 42 (11.3) | 8 (23.5) | 11 (19.6) | 6 (4.1) | – | – | 5 (13.9) | 8 (9.8) |
|  | IMID–PI | 37 (10.0) | 2 (5.9) | 20 (35.7) | 1 (0.7) | – | – | 3 (8.3) | 10 (12.2) |
|  | Chemotherapies–IMID–PI | 1 (0.3) | – | 1 (1.8) | – | – | – | – | – |
|  | IMID–mAb | 1 (0.3) | – | – | – | – | – | – | – |

For the D8-like cohort, data for index lines Kd, KRd, IxaRd and PanoVd not reported due to small group size. IMID: lenalidomide, pomalidomide, thalidomide; PI: bortezomib, carfilzomib, ixazomib; mAb: rituximab.

Abbreviations: 1L, first-line; 2L, second-line; D7, DREAMM-7; D8, DREAMM-8; DaraVd, daratumumab–bortezomib–dexamethasone; IMID, immunomodulatory drugs; IxaRd, ixazomib–lenalidomide–dexamethasone; Kd, carfilzomib–dexamethasone; KRd, carfilzomib–lenalidomide–dexamethasone; mAb, monoclonal antibody; PanoVd, panobinostat–bortezomib–dexamethasone; PI, proteasome inhibitor; PomDex, pomalidomide–dexamethasone; Rd, lenalidomide–dexamethasone.

TABLE S6. Subsequent treatment regimens and drug classes for patients at 2L in the D7-like cohort

| **Lenalidomide-exposed** | | **3L** | **4L** | **5L** | **6L+** |
| --- | --- | --- | --- | --- | --- |
| Regimen | Pomalidomide | 109 (34.5) | 39 (32.2) | 8 (21.6) | 4 (22.2) |
|  | Bortezomib–panobinostat | 18 (5.7) | 21 (17.4) | 12 (32.4) | 3 (16.7) |
|  | Daratumumab | 32 (10.1) | 15 (12.4) | 2 (5.4) | – |
|  | Lenalidomide | 27 (8.5) | 6 (5.0) | 2 (5.4) | – |
|  | Cyclophosphamide–thalidomide | 16 (5.1) | 7 (5.8) | 2 (5.4) | 1 (5.6) |
|  | Ixazomib–lenalidomide | 19 (6.0) | 3 (2.5) | – | – |
|  | Bortezomib–cyclophosphamide | 17 (5.4) | 1 (0.8) | 1 (2.7) | – |
|  | Bortezomib–daratumumab | 15 (4.7) | 1 (0.8) | – | – |
|  | Melphalan–thalidomide | 9 (2.8) | 5 (4.1) | – | 2 (11.1) |
|  | Bortezomib | 13 (4.1) | – | – | – |
|  | Cyclophosphamide–pomalidomide | 8 (2.5) | 1 (0.8) | 2 (5.4) | – |
|  | Bendamustine–thalidomide | 5 (1.6) | – | 2 (5.4) | 2 (11.1) |
|  | Bendamustine | 3 (0.9) | 2 (1.7) | 1 (2.7) | – |
|  | Melphalan | 3 (0.9) | 2 (1.7) | – | 1 (5.6) |
|  | Belantamab mafodotin | – | 3 (2.5) | 1 (2.7) | 2 (11.1) |
|  | Bortezomib–panobinostat–thalidomide | 2 (0.6) | – | 1 (2.7) | 1 (5.6) |
|  | Cisplatin–cyclophosphamide–doxorubicin–etoposide–thalidomide | 3 (0.9) | 1 (0.8) | – | – |
|  | Bortezomib–cisplatin–cyclophosphamide–doxorubicin–etoposide–thalidomide | – | 3 (2.5) | – | 1 (5.6) |
|  | Thalidomide | – | 3 (2.5) | 1 (2.7) | – |
|  | Bortezomib–thalidomide | 3 (0.9) | – | – | – |
|  | Cyclophosphamide | 1 (0.3) | 2 (1.7) | – | – |
|  | Cyclophosphamide–lenalidomide | 2 (0.6) | 1 (0.8) | – | – |
|  | Isatuximab–pomalidomide | 2 (0.6) | 1 (0.8) | – | – |
|  | Ixazomib | 2 (0.6) | – | – | 1 (5.6) |
|  | Bortezomib–melphalan | 2 (0.6) | – | – | – |
|  | Bortezomib–doxorubicin | 1 (0.3) | – | – | – |
|  | Bortezomib–pomalidomide | 1 (0.3) | – | – | – |
|  | Carfilzomib | 1 (0.3) | – | – | – |
|  | Cyclophosphamide–doxorubicin–vincristine | 1 (0.3) | – | – | – |
|  | Cyclophosphamide–pomalidomide–thalidomide | 1 (0.3) | – | – | – |
|  | Bortezomib–cisplatin–cyclophosphamide–doxorubicin–etoposide–lenalidomide–thalidomide | – | 1 (0.8) | – | – |
|  | Bortezomib–daratumumab–panobinostat | – | 1 (0.8) | – | – |
|  | Bortezomib–daratumumab–thalidomide | – | 1 (0.8) | – | – |
|  | Cyclophosphamide–doxorubicin–rituximab–vincristine | – | 1 (0.8) | – | – |
|  | Cyclophosphamide-daratumumab-pomalidomide | – | – | 1 (2.7) | – |
|  | Cyclophosphamide-fludarabine | – | – | 1 (2.7) | – |
| Drug classes | IMID | 136 (43.0) | 48 (39.7) | 11 (29.7) | 4 (22.2) |
|  | Chemotherapies–IMID | 44 (13.9) | 15 (12.4) | 6 (16.2) | 5 (27.8) |
|  | HDAC–PI | 18 (5.7) | 21 (17.4) | 12 (32.4) | 3 (16.7) |
|  | Anti-CD38 mAb | 32 (10.1) | 15 (12.4) | 2 (5.4) | – |
|  | IMID–PI | 23 (7.3) | 3 (2.5) | – | – |
|  | Chemotherapies–PI | 20 (6.3) | 1 (0.8) | 1 (2.7) | – |
|  | Chemotherapies | 8 (2.5) | 6 (5.0) | 2 (5.4) | 1 (5.6) |
|  | PI | 16 (5.1) | – | – | 1 (5.6) |
|  | Anti-CD38 mAb–PI | 15 (4.7) | 1 (0.8) | – | – |
|  | BCMA | – | 3 (2.5) | 1 (2.7) | 2 (11.1) |
|  | Chemotherapies–IMID–PI | – | 4 (3.3) | – | 1 (5.6) |
|  | HDAC–IMID–PI | 2 (0.6) | – | 1 (2.7) | 1 (5.6) |
|  | Anti-CD38 mAb–IMID | 2 (0.6) | 1 (0.8) | – | – |
|  | Anti-CD38 mAb–HDAC–PI | – | 1 (0.8) | – | – |
|  | Anti-CD38 mAb–IMID–PI | – | 1 (0.8) | – | – |
|  | Chemotherapies–mAb | – | 1 (0.8) | – | – |
|  | Anti-CD38 mAb–chemotherapies–IMID | – | – | 1 (2.7) | – |
| **Lenalidomide-refractory** | | **3L** | **4L** | **5L+** |  |
| Regimen | Pomalidomide | 72 (46.8) | 12 (22.6) | – |  |
|  | Bortezomib–panobinostat | 8 (5.2) | 15 (28.3) | 3 (25.0) |  |
|  | Daratumumab | 12 (7.8) | 5 (9.4) | – |  |
|  | Cyclophosphamide–thalidomide | 9 (5.8) | 2 (3.8) | 2 (16.7) |  |
|  | Bortezomib–daratumumab | 10 (6.5) | 1 (1.9) | – |  |
|  | Lenalidomide | 8 (5.2) | 1 (1.9) | – |  |
|  | Bortezomib–cyclophosphamide | 6 (3.9) | – | 1 (8.3) |  |
|  | Melphalan–thalidomide | 3 (1.9) | 4 (7.5) | – |  |
|  | Cyclophosphamide–pomalidomide | 5 (3.2) | 1 (1.9) | – |  |
|  | Bendamustine | 3 (1.9) | 2 (3.8) | – |  |
|  | Ixazomib–lenalidomide | 4 (2.6) | 1 (1.9) | – |  |
|  | Bendamustine–thalidomide | 2 (1.3) | – | 1 (8.3) |  |
|  | Isatuximab–pomalidomide | 2 (1.3) | 1 (1.9) | – |  |
|  | Melphalan | 1 (0.6) | 2 (3.8) | – |  |
|  | Thalidomide | – | 2 (3.8) | 1 (8.3) |  |
|  | Bortezomib | 2 (1.3) | – | – |  |
|  | Bortezomib–panobinostat–thalidomide | 1 (0.6) | – | 1 (8.3) |  |
|  | Belantamab mafodotin | – | 1 (1.9) | 1 (8.3) |  |
|  | Bortezomib–melphalan | 1 (0.6) | – | – |  |
|  | Bortezomib–pomalidomide | 1 (0.6) | – | – |  |
|  | Bortezomib–thalidomide | 1 (0.6) | – | – |  |
|  | Cisplatin–cyclophosphamide–doxorubicin–etoposide–thalidomide | 1 (0.6) | – | – |  |
|  | Cyclophosphamide–doxorubicin–vincristine | 1 (0.6) | – | – |  |
|  | Cyclophosphamide–pomalidomide–thalidomide | 1 (0.6) | – | – |  |
|  | Bortezomib–cisplatin–cyclophosphamide–doxorubicin–etoposide–thalidomide | – | 1 (1.9) | – |  |
|  | Cyclophosphamide | – | 1 (1.9) | – |  |
|  | Cyclophosphamide–doxorubicin–rituximab–vincristine | – | 1 (1.9) | – |  |
|  | Cyclophosphamide–fludarabine | – | – | 1 (8.3) |  |
|  | Ixazomib | – | – | 1 (8.3) |  |
| Drug classes | IMID | 80 (51.9) | 15 (28.3) | 1 (8.3) |  |
|  | Chemotherapies–IMID | 21 (13.6) | 7 (13.2) | 3 (25.0) |  |
|  | HDAC–PI | 8 (5.2) | 15 (28.3) | 3 (25.0) |  |
|  | Anti-CD38 mAb | 12 (7.8) | 5 (9.4) | – |  |
|  | Anti-CD38 mAb–PI | 10 (6.5) | 1 (1.9) | – |  |
|  | Chemotherapies | 5 (3.2) | 5 (9.4) | 1 (8.3) |  |
|  | Chemotherapies–PI | 7 (4.5) | – | 1 (8.3) |  |
|  | IMID–PI | 6 (3.9) | 1 (1.9) | – |  |
|  | Anti-CD38 mAb–IMID | 2 (1.3) | 1 (1.9) | – |  |
|  | PI | 2 (1.3) | – | 1 (8.3) |  |
|  | HDAC–IMID–PI | 1 (0.6) | – | 1 (8.3) |  |
|  | BCMA | – | 1 (1.9) | 1 (8.3) |  |
|  | Chemotherapies–IMID–PI | – | 1 (1.9) | – |  |
|  | Chemotherapies–mAb | – | 1 (1.9) | – |  |

IMID: lenalidomide, pomalidomide, thalidomide; PI: bortezomib, carfilzomib, ixazomib; Anti-CD38 mAb: daratumumab, isatuximab; HDAC: panobinostat.

Abbreviations: 2L, second-line; 3L, third-line; 4L, fourth-line; 5L, fifth-line; 6L, sixth-line; BCMA, B-cell maturation antigen; D7, DREAMM-7; HDAC, histone deacetylase inhibitor; IMID, immunomodulatory drug; mAb, monoclonal antibody; PI, proteasome inhibitor.

TABLE S7. Subsequent lines of therapy received at 2L and 3L in the D8-like cohort

| **Lenalidomide-exposed (2L)** | | **Total** | **Bortezomib** | **Kd** | **KRd** | **Daratumumab** | **DaraVd** | **IxaRd** | **Rd** | **PanoVd** | **PomDex** | **Other** |
| --- | --- | --- | --- | --- | --- | --- | --- | --- | --- | --- | --- | --- |
| Total patients; *N* (%) | | 730 (100.0) | 67 (9.2) | ^a^ | 0 (0.0) | 103 (14.1) | 269 (36.8) | ^a^ | 32 (4.4) | ^a^ | 66 (9.0) | 152 (20.8) |
| Number of subsequent lines | Median (IQR) | 0 (0, 1) | 0 (0, 1) | – | – | 1 (0, 1) | 0 (0, 1) | – | 0 (0, 1) | – | 0 (0, 1) | 1 (0, 1) |
|  | 0 | 449 (61.5) | 40 (59.7) | – | – | 47 (45.6) | 198 (73.6) | – | 23 (71.9) | – | 43 (65.2) | 71 (46.7) |
|  | 1 | 179 (24.5) | 17 (25.4) | – | – | 35 (34.0) | 54 (20.1) | – | 3 (9.4) | – | 13 (19.7) | 52 (34.2) |
|  | 2 | 69 (9.5) | 7 (10.4) | – | – | 12 (11.7) | 14 (5.2) | – | 4 (12.5) | – | 9 (13.6) | 18 (11.8) |
|  | 3+ | 33 (4.5) | 3 (4.5) | – | – | 9 (8.7) | 3 (1.1) | – | 2 (6.2) | – | 1 (1.5) | 11 (7.2) |
| Subsequent 3L meeting D8-like eligibility criteria received in follow-up | Yes | 166 (22.7) | 13 (19.4) | – | – | 56 (54.4) | 11 (4.1) | – | 9 (28.1) | – | 0 (0.0) | 65 (42.8) |
| **Lenalidomide-refractory (2L)** | | **Total** | **Bortezomib** | **Kd** | **KRd** | **Daratumumab** | **DaraVd** | **IxaRd** | **Rd** | **PanoVd** | **PomDex** | **Other** |
| Total patients; *N* (%) |  | 371 (100.0) | 34 (9.2) | ^a^ | 0 (0.0) | 56 (15.1) | 148 (39.9) | ^a^ | ^a^ | ^a^ | 36 (9.7) | 82 (22.1) |
| Number of subsequent lines | Median (IQR) | 0 (0, 1) | 0.5 (0, 1) | – | – | 1 (0, 1) | 0 (0, 1) | – | – | – | 0 (0, 1) | 1 (0, 1) |
|  | 0 | 209 (56.3) | 17 (50.0) | – | – | 23 (41.1) | 100 (67.6) | – | – | – | 21 (58.3) | 38 (46.3) |
|  | 1 | 109 (29.4) | 12 (35.3) | – | – | 23 (41.1) | 34 (23.0) | – | – | – | 8 (22.2) | 31 (37.8) |
|  | 2 | 40 (10.8) | 4 (11.8) | – | – | 7 (12.5) | 12 (8.1) | – | – | – | 6 (16.7) | 8 (9.8) |
|  | 3+ | 13 (3.5) | 1 (2.9) | – | – | 3 (5.4) | 2 (1.4) | – | – | – | 1 (2.8) | 5 (6.1) |
| Subsequent 3L meeting D8-like eligibility criteria received in follow-up | Yes | 85 (22.9) | 5 (14.7) | – | – | 8 (14.3) | 2 (1.4) | – | – | – | 0 (0.0) | 34 (41.5) |
| **Lenalidomide-exposed (3L)** | | **Total** | **Bortezomib** | **Kd** | **KRd** | **Daratumumab** | **DaraVd** | **IxaRd** | **Rd** | **PanoVd** | **PomDex** | **Other** |
| Total patients; *N* (%) |  | 1344 (100.0) | 36 (2.7) | ^a^ | ^a^ | 423 (31.5) | 124 (9.2) | 74 (5.5) | 48 (3.6) | 84 (6.2) | 304 (22.6) | 241 (17.9) |
| Number of subsequent lines | Median (IQR) | 0 (0, 1) | 1 (0, 2) | – | – | 1 (0, 1) | 0 (0, 1) | 0 (0, 1) | 0 (0, 0) | 0 (0, 1) | 0 (0, 1) | 0 (0, 1) |
|  | 0 | 748 (55.7) | 16 (44.4) | – | – | 182 (43.0) | 87 (70.2) | 41 (55.4) | 37 (77.1) | 44 (52.4) | 204 (67.1) | 130 (53.9) |
|  | 1 | 384 (28.6) | 10 (27.8) | – | – | 155 (36.6) | 30 (24.2) | 15 (20.3) | 9 (18.8) | 24 (28.6) | 71 (23.4) | 69 (28.6) |
|  | 2 | 156 (11.6) | 5 (13.9) | – | – | 68 (16.1) | 6 (4.8) | 9 (12.2) | 1 (2.1) | 13 (15.5) | 26 (8.6) | 27 (11.2) |
|  | 3+ | 56 (4.2) | 5 (13.9) | – | – | 18 (4.3) | 1 (0.8) | 9 (12.2) | 1 (2.1) | 3 (3.6) | 3 (1.0) | 15 (6.2) |
| Prior 2L meeting D8-like eligibility criteria received | Yes | 166 (12.4) | 6 (16.7) | – | – | 21 (5.0) | 11 (8.9) | 9 (12.2) | 18 (37.5) | 11 (13.1) | 63 (20.7) | 26 (10.8) |
| **Lenalidomide-refractory (3L)** | | **Total** | **Bortezomib** | **Kd** | **KRd** | **Daratumumab** | **DaraVd** | **IxaRd** | **Rd** | **PanoVd** | **PomDex** | **Other** |
| Total patients; *N* (%) |  | 776 (100.0) | 21 (2.7) | ^a^ | ^a^ | 253 (32.6) | 77 (9.9) | 34 (4.4) | * | 53 (6.8) | 176 (22.7) | 149 (19.2) |
| Number of subsequent lines | Median (IQR) | 0 (0, 1) | 1 (0, 1) | – | – | 1 (0, 1) | 0 (0, 1) | 0.5 (0, 1) | – | 0 (0, 1) | 0 (0, 1) | 0 (0, 1) |
|  | 0 | 410 (52.8) | 10 (47.6) | – | – | 98 (38.7) | 52 (67.5) | 17 (50.0) | – | 31 (58.5) | 111 (63.1) | 79 (53.0) |
|  | 1 | 233 (30.0) | 6 (28.6) | – | – | 97 (38.3) | 19 (24.7) | 9 (26.5) | – | 12 (22.6) | 48 (27.3) | 42 (28.2) |
|  | 2 | 101 (13.0) | 3 (14.3) | – | – | 46 (18.2) | 5 (6.5) | 5 (14.7) | – | 8 (15.1) | 15 (8.5) | 18 (12.1) |
|  | 3+ | 32 (4.1) | 2 (9.5) | – | – | 12 (4.7) | 1 (1.3) | 3 (8.8) | – | 2 (3.8) | 2 (1.1) | 10 (6.7) |
| Prior 2L meeting D8-like eligibility criteria received | Yes | 85 (11.0) | 1 (4.8) | – | – | 12 (4.7) | 7 (9.1) | 2 (5.9) | – | 5 (9.4) | 38 (21.6) | 10 (6.7) |

Abbreviations: 2L, second-line; 3L, third-line; D8, DREAMM-8; DaraVd, daratumumab–bortezomib–dexamethasone; IQR, interquartile range; IxaRd, ixazomib–lenalidomide–dexamethasone; Kd, carfilzomib–dexamethasone; KRd, carfilzomib–lenalidomide–dexamethasone; PanoVd, panobinostat–bortezomib–dexamethasone; PomDex, pomalidomide–dexamethasone; Rd, lenalidomide–dexamethasone.

^a^Small cohort–data not reported for this group.

TABLE S8. Clinical outcomes of patients treated at 2L in the overall D7-like cohort (non-NICE-approved regimens)

|  | **Daratumumab** | **IxaRd** | **PanoVd** | **Pomalidomide** | **Other** |
| --- | --- | --- | --- | --- | --- |
| TTNTD | | | | | |
| Total patients*, N* | 294 | 1216 | 65 | 155 | 2879 |
| Number of events*, n* | 229 | 655 | 63 | 133 | 2532 |
| Number censored*, n* | 65 | 561 | 2 | 22 | 347 |
| Median (95% CI) TTNTD; months | 8.1 (6.1, 10.6) | 17.9 (16.3, 20.3) | 4.2 (3.1, 6.2) | 7.2 (5.6, 10) | 11.1 (10.6, 11.8) |
| Months from line start | *N* at risk | | | | |
| 1 | 270 | 1176 | 60 | 147 | 2775 |
| 6 | 155 | 888 | 24 | 83 | 1953 |
| 12 | 106 | 588 | – | 50 | 1330 |
| 18 | 74 | 389 | – | 32 | 835 |
| 24 | 53 | 219 | – | 27 | 552 |
| 36 | 16 | 100 | – | 14 | 264 |
| 48 | – | 37 | – | 10 | 121 |
| 60 | – | – | – | – | 69 |
|  | **Daratumumab** | **IxaRd** | **PanoVd** | **Pomalidomide** | **Other** |
| TTDD | | | | | |
| Total patients*, N* | 294 | 1216 | 65 | 155 | 2879 |
| Number of events*, n* | 251 | 929 | 63 | 149 | 2812 |
| Number censored*, n* | 43 | 287 | 2 | 6 | 67 |
| Median (95% CI) TTDD; months | 4.8 (4.1, 5.4) | 9.6 (8.6, 10.6) | 3 (1.8, 3.7) | 3.6 (2.7, 4.8) | 4.3 (4.1, 4.4) |
| Months from line start | *N* at risk | | | | |
| 1 | 270 | 1174 | 52 | 145 | 2747 |
| 6 | 119 | 734 | 11 | 54 | 889 |
| 12 | 68 | 446 | – | 34 | 226 |
| 18 | 46 | 269 | – | 24 | 121 |
| 24 | 32 | 151 | – | 17 | 78 |
| 36 | – | 61 | – | – | 24 |
| 48 | – | 18 | – | – | – |
|  | **Daratumumab** | **IxaRd** | **PanoVd** | **Pomalidomide** | **Other** |
| OS | | | | | |
| Total patients*, N* | 294 | 1216 | 65 | 155 | 2879 |
| Number of events*, n* | 175 | 516 | 58 | 119 | 1967 |
| Number censored*, n* | 119 | 700 | 7 | 36 | 912 |
| Median (95% CI) OS; months | 24.6 (19.6, 28.9) | 31.5 (27.9, 35.4) | 5.6 (3.7, 8.7) | 10 (7.3, 15.5) | 27.3 (25.7, 29.2) |
| Months from line start | *N* at risk | | | | |
| 1 | 279 | 1177 | 61 | 147 | 2808 |
| 6 | 221 | 975 | 29 | 96 | 2459 |
| 12 | 172 | 747 | 19 | 63 | 2079 |
| 18 | 144 | 536 | 13 | 45 | 1706 |
| 24 | 115 | 341 | 12 | 37 | 1372 |
| 36 | 60 | 176 | – | 22 | 918 |
| 48 | 14 | 72 | – | 15 | 517 |
| 60 | *–* | – | – | – | 284 |

TTNTD event is the earliest of a new line start or death during follow-up. Patients remaining on the line of therapy or yet to start a new line and alive at follow-up end were censored. TTDD event is the earliest of: last administration plus one cycle length; start of a new line minus 1 day; death during follow-up. Patients alive at follow-up end with no subsequent line and last administration date of the line within a cycle length of administrative follow-up end were censored. OS event is death during follow-up. Patients alive at follow-up were censored.

Abbreviations: 2L, second-line; CI, confidence interval; D7, DREAMM-7; IxaRd, ixazomib–lenalidomide–dexamethasone; NICE, National Institute for Health and Care Excellence; OS, overall survival; PanoVd, panobinostat–bortezomib–dexamethasone; TTDD, time to treatment discontinuation or death; TTNTD, time to next treatment or death.

TABLE S9. Baseline characteristics of patients treated at 3L in the D8-like cohort by regimen type

| **D8-like lenalidomide-exposed** | | **Total** | **Vd** | **Kd** | **KRd** | **Daratumumab** | **DaraVd** | **IxaRd** | **Rd** | **PanoVd** | **PomDex** | **Other** |
| --- | --- | --- | --- | --- | --- | --- | --- | --- | --- | --- | --- | --- |
| Total patients; *N* (%) | | 1344 (100.0) | 36 (2.7) | ^a^ | ^a^ | 423 (31.5) | 124 (9.2) | 74 (5.5) | 48 (3.6) | 84 (6.2) | 304 (22.6) | 241 (17.9) |
| Age at line start; mean (SD) | Years | 71.9 (10.3) | 73.9 (10.3) | – | – | 72.0 (9.7) | 67.1 (9.8) | 70.2 (9.1) | 65.5 (11.7) | 72.0 (9.5) | 75.4 (9.6) | 71.5 (11.0) |
| Age at line start; *N* (%) | 18–44 | 15 (1.1) |  | – | – | 2 (0.5) | 3 (2.4) | – | 2 (4.2) | 1 (1.2) | 2 (0.7) | 5 (2.1) |
|  | 45–64 | 275 (20.5) | 4 (11.1) | – | – | 86 (20.3) | 42 (33.9) | 17 (23.0) | 19 (39.6) | 16 (19.0) | 35 (11.5) | 49 (20.3) |
|  | 65–74 | 454 (33.8) | 12 (33.3) | – | – | 150 (35.5) | 44 (35.5) | 31 (41.9) | 17 (35.4) | 34 (40.5) | 88 (28.9) | 76 (31.5) |
|  | 75–84 | 476 (35.4) | 15 (41.7) | – | – | 154 (36.4) | 32 (25.8) | 22 (29.7) | 7 (14.6) | 25 (29.8) | 133 (43.8) | 87 (36.1) |
|  | 85+ | 124 (9.2) | 5 (13.9) | – | – | 31 (7.3) | 3 (2.4) | 4 (5.4) | 3 (6.2) | 8 (9.5) | 46 (15.1) | 24 (10.0) |
| Gender; *N* (%) | Male | 788 (58.6) | 21 (58.3) | – | – | 238 (56.3) | 69 (55.6) | 46 (62.2) | 26 (54.2) | 41 (48.8) | 184 (60.5) | 157 (65.1) |
|  | Female | 556 (41.4) | 15 (41.7) | – | – | 185 (43.7) | 55 (44.4) | 28 (37.8) | 22 (45.8) | 43 (51.2) | 120 (39.5) | 84 (34.9) |
| Ethnicity; *N* (%) | Asian | 42 (3.1) | 1 (2.8) | – | – | 15 (3.5) | 4 (3.2) | 2 (2.7) | 2 (4.2) | 5 (6.0) | 7 (2.3) | 6 (2.5) |
|  | Black | 52 (3.9) | – | – | – | 13 (3.1) | 9 (7.3) | 5 (6.8) | 3 (6.2) | 7 (8.3) | 6 (2.0) | 8 (3.3) |
|  | White | 1184 (88.1) | 33 (91.7) | – | – | 384 (90.8) | 102 (82.3) | 61 (82.4) | 37 (77.1) | 68 (81.0) | 273 (89.8) | 218 (90.5) |
|  | Other | 31 (2.3) | 2 (5.6) | – | – | 5 (1.2) | 5 (4.0) | 1 (1.4) | 3 (6.2) | 2 (2.4) | 8 (2.6) | 5 (2.1) |
|  | Unknown | 35 (2.6) | – | – | – | 6 (1.4) | 4 (3.2) | 5 (6.8) | 3 (6.2) | 2 (2.4) | 10 (3.3) | 4 (1.7) |
| Stage; *N* (%) | 1 | 96 (7.1) | 2 (5.6) | – | – | 26 (6.1) | 13 (10.5) | 5 (6.8) | 6 (12.5) | 3 (3.6) | 20 (6.6) | 19 (7.9) |
|  | 2 | 145 (10.8) | 5 (13.9) | – | – | 44 (10.4) | 16 (12.9) | 10 (13.5) | 5 (10.4) | 9 (10.7) | 33 (10.9) | 22 (9.1) |
|  | 3 | 129 (9.6) | 3 (8.3) | – | – | 48 (11.3) | 13 (10.5) | 2 (2.7) | 4 (8.3) | 9 (10.7) | 30 (9.9) | 20 (8.3) |
|  | Unstaged | 974 (72.5) | 26 (72.2) | – | – | 305 (72.1) | 82 (66.1) | 57 (77.0) | 33 (68.8) | 63 (75.0) | 221 (72.7) | 180 (74.7) |
| ECOG performance status; *N* (%) | 0 | 219 (16.3) | 4 (11.1) | – | – | 81 (19.1) | 19 (15.3) | 16 (21.6) | 12 (25.0) | 7 (8.3) | 38 (12.5) | 41 (17.0) |
|  | 1 | 501 (37.3) | 17 (47.2) | – | – | 172 (40.7) | 47 (37.9) | 24 (32.4) | 13 (27.1) | 31 (36.9) | 96 (31.6) | 97 (40.2) |
|  | 2 | 219 (16.3) | 5 (13.9) | – | – | 63 (14.9) | 15 (12.1) | 7 (9.5) | 4 (8.3) | 16 (19.0) | 72 (23.7) | 37 (15.4) |
|  | 3–4 | 25 (1.9) | 1 (2.8) | – | – | 5 (1.2) | 1 (0.8) | 2 (2.7) | – | 3 (3.6) | 4 (1.3) | 9 (3.7) |
|  | Unknown | 380 (28.3) | 9 (25.0) | – | – | 102 (24.1) | 42 (33.9) | 25 (33.8) | 19 (39.6) | 27 (32.1) | 94 (30.9) | 57 (23.7) |
| ASCT; *N* (%) | No | 936 (69.6) | 29 (80.6) | – | – | 289 (68.3) | 58 (46.8) | 47 (63.5) | 15 (31.2) | 62 (73.8) | 255 (83.9) | 179 (74.3) |
|  | Yes | 408 (30.4) | 7 (19.4) | – | – | 134 (31.7) | 66 (53.2) | 27 (36.5) | 33 (68.8) | 22 (26.2) | 49 (16.1) | 62 (25.7) |
| Lytic bone lesion; *N* (%) | No | 1304 (97.0) | 35 (97.2) | – | – | 410 (96.9) | 117 (94.4) | 73 (98.6) | 47 (97.9) | 83 (98.8) | 297 (97.7) | 232 (96.3) |
|  | Yes | 40 (3.0) | 1 (2.8) | – | – | 13 (3.1) | 7 (5.6) | 1 (1.4) | 1 (2.1) | 1 (1.2) | 7 (2.3) | 9 (3.7) |
| Extramedullary plasmacytoma; *N* (%) | No | 1335 (99.3) | 36 (100.0) | – | – | 420 (99.3) | 124 (100.0) | 73 (98.6) | 48 (100.0) | 80 (95.2) | 304 (100.0) | 240 (99.6) |
|  | Yes | 9 (0.7) | – | – | – | 3 (0.7) | – | 1 (1.4) | 0 (0.0) | 4 (4.8) | – | 1 (0.4) |
| Prior PI exposure; *N* (%) | No | 193 (14.4) | 19 (52.8) | – | – | 33 (7.8) | 11 (8.9) | 11 (14.9) | 12 (25.0) | 10 (11.9) | 53 (17.4) | 42 (17.4) |
|  | Yes | 1151 (85.6) | 17 (47.2) | – | – | 390 (92.2) | 113 (91.1) | 63 (85.1) | 36 (75.0) | 74 (88.1) | 251 (82.6) | 199 (82.6) |
| Prior IMID exposure; *N* (%) | No | – | – | – | – | – | – | – | – | – | – | – |
|  | Yes | 1344 (100.0) | 36 (100.0) | – | – | 423 (100.0) | 124 (100.0) | 74 (100.0) | 48 (100.0) | 84 (100.0) | 304 (100.0) | 241 (100.0) |
| Prior anti-CD38 mAb exposure; *N* (%) | No | 1265 (94.1) | 34 (94.4) | – | – | 422 (99.8) | 123 (99.2) | 73 (98.6) | 45 (93.8) | 78 (92.9) | 251 (82.6) | 229 (95.0) |
|  | Yes | 79 (5.9) | 2 (5.6) | – | – | 1 (0.2) | 1 (0.8) | 1 (1.4) | 3 (6.2) | 6 (7.1) | 53 (17.4) | 12 (5.0) |
| Prior lenalidomide exposure; *N* (%) | No | – | – | – | – |  |  |  |  |  |  |  |
|  | Yes | 1344 (100.0) | 36 (100.0) | – | – | 423 (100.0) | 124 (100.0) | 74 (100.0) | 48 (100.0) | 84 (100.0) | 304 (100.0) | 241 (100.0) |
| Lenalidomide refractory; *N* (%) | No | 568 (42.3) | 15 (41.7) | – | – | 170 (40.2) | 47 (37.9) | 40 (54.1) | 37 (77.1) | 31 (36.9) | 128 (42.1) | 92 (38.2) |
|  | Yes | 776 (57.7) | 21 (58.3) | – | – | 253 (59.8) | 77 (62.1) | 34 (45.9) | 11 (22.9) | 53 (63.1) | 176 (57.9) | 149 (61.8) |
| Available follow-up from MM diagnosis; median (IQR) | Months | 52.9 (36.5 to 75.4) | 55.3 (37.9 to 70.7) | – | – | 63.8 (46.2 to 84.3) | 36.9 (30.4 to 53.3) | 55.7 (44.9 to 80.4) | 43.5 (34.5 to 82.3) | 45.0 (33.2 to 62.3) | 50.6 (36.2 to 73.6) | 47.2 (33.3 to 68.8) |
| Available follow-up from start of line; median (IQR) | Months | 9.1 (3.6 to 19.5) | 13.8 (5.0 to 21.8) | – | – | 12.0 (4.0 to 24.9) | 6.5 (3.2 to 11.5) | 14.6 (5.3 to 26.8) | 14.5 (10.9 to 23.5) | 5.0 (2.2 to 14.3) | 7.3 (3.1 to 14.3) | 8.6 (3.4 to 18.2) |
| **D8-like lenalidomide-refractory** | | **Total** | **Vd** | **Kd** | **KRd** | **Daratumumab** | **DaraVd** | **IxaRd** | **Rd** | **PanoVd** | **PomDex** | **Other** |
| Total patients; *N* (%) | | 776 (100.0) | 21 (2.7) | ^a^ | ^a^ | 253 (32.6) | 77 (9.9) | 34 (4.4) | ^a^ | 53 (6.8) | 176 (22.7) | 149 (19.2) |
| Age at line start; mean (SD) | Years | 71.4 (10.3) | 75.5 (10.5) | – | – | 71.0 (9.7) | 66.4 (9.9) | 70.1 (9.3) | – | 71.8 (10.4) | 75.3 (9.4) | 70.2 (11.0) |
| Age at line start; *N* (%) | 18–44 | 9 (1.2) | – | – | – | 1 (0.4) | 2 (2.6) | – | – | 1 (1.9) | 1 (0.6) | 3 (2.0) |
|  | 45–64 | 170 (21.9) | ^a^ | – | – | 60 (23.7) | 29 (37.7) | 6 (17.6) | – | 12 (22.6) | 18 (10.2) | 38 (25.5) |
|  | 65–74 | 271 (34.9) | ^a^ | – | – | 89 (35.2) | 29 (37.7) | 18 (52.9) | – | 16 (30.2) | 55 (31.2) | 52 (34.9) |
|  | 75–84 | 265 (34.1) | ^a^ | – | – | 90 (35.6) | 15 (19.5) | 8 (23.5) | – | 20 (37.7) | 77 (43.8) | 45 (30.2) |
|  | 85+ | 61 (7.9) | ^a^ | – | – | 13 (5.1) | 2 (2.6) | 2 (5.9) | – | 4 (7.5) | 25 (14.2) | 11 (7.4) |
| Gender; *N* (%) | Male | 454 (58.5) | 12 (57.1) | – | – | 142 (56.1) | 47 (61.0) | 22 (64.7) | – | 26 (49.1) | 105 (59.7) | 93 (62.4) |
|  | Female | 322 (41.5) | 9 (42.9) | – | – | 111 (43.9) | 30 (39.0) | 12 (35.3) | – | 27 (50.9) | 71 (40.3) | 56 (37.6) |
| Ethnicity; *N* (%) | Asian | 31 (4.0) | ^a^ | – | – | 10 (4.0) | 4 (5.2) | 1 (2.9) | – | 5 (9.4) | 3 (1.7) | 5 (3.4) |
|  | Black | 31 (4.0) | – | – | – | 10 (4.0) | 6 (7.8) | 1 (2.9) | – | 6 (11.3) | 2 (1.1) | 6 (4.0) |
|  | White | 678 (87.4) | ^a^ | – | – | 226 (89.3) | 61 (79.2) | 32 (94.1) | – | 38 (71.7) | 160 (90.9) | 133 (89.3) |
|  | Other | 20 (2.6) | ^a^ | – | – | 3 (1.2) | 4 (5.2) | 0 (0.0) | – | 2 (3.8) | 6 (3.4) | 3 (2.0) |
|  | Unknown | 16 (2.1) | – | – | – | 4 (1.6) | 2 (2.6) | 0 (0.0) | – | 2 (3.8) | 5 (2.8) | 2 (1.3) |
| Stage^b^; *N* (%) | 1 | 57 (7.3) | ^a^ | – | – | 28 (11.1) | 7 (9.1) | 2 (5.9) | – | 6 (11.3) | 24 (13.6) | 11 (7.4) |
|  | 2 | 84 (10.8) | ^a^ | – | – | 32 (12.6) | 7 (9.1) | 2 (5.9) | – | 7 (13.2) | 19 (10.8) | 13 (8.7) |
|  | 3 | 83 (10.7) | ^a^ | – | – | 174 (68.8) | 56 (72.7) | 26 (76.5) | – | 38 (71.7) | 122 (69.3) | 112 (75.2) |
|  | Unstaged | 552 (71.1) | – | – | – | 19 (7.5) | 7 (9.1) | 4 (11.8) | – | 2 (3.8) | 11 (6.2) | 13 (8.7) |
| ECOG performance status; *N* (%) | 0 | 123 (15.9) | ^a^ | – | – | 46 (18.2) | 10 (13.0) | 9 (26.5) | – | 6 (11.3) | 20 (11.4) | 28 (18.8) |
|  | 1 | 298 (38.4) | 10 (47.6) | – | – | 108 (42.7) | 31 (40.3) | 12 (35.3) | – | 20 (37.7) | 54 (30.7) | 57 (38.3) |
|  | 2 | 137 (17.7) | ^a^ | – | – | 37 (14.6) | 10 (13.0) | 4 (11.8) | – | 12 (22.6) | 46 (26.1) | 23 (15.4) |
|  | 3–4 | 15 (1.9) | ^a^ | – | – | 2 (0.8) | 1 (1.3) | – | – | 1 (1.9) | 3 (1.7) | 7 (4.7) |
|  | Unknown | 203 (26.2) | ^a^ | – | – | 60 (23.7) | 25 (32.5) | 9 (26.5) | – | 14 (26.4) | 53 (30.1) | 34 (22.8) |
| ASCT; *N* (%) | No | 526 (67.8) | ^a^ | – | – | 157 (62.1) | 33 (42.9) | 20 (58.8) | – | 37 (69.8) | 149 (84.7) | 106 (71.1) |
|  | Yes | 250 (32.2) | ^a^ | – | – | 96 (37.9) | 44 (57.1) | 14 (41.2) | – | 16 (30.2) | 27 (15.3) | 43 (28.9) |
| Lytic bone lesion; *N* (%) | No | 752 (96.9) | ^a^ | – | – | 247 (97.6) | 72 (93.5) | 33 (97.1) | – | 52 (98.1) | 172 (97.7) | 143 (96.0) |
|  | Yes | 24 (3.1) | ^a^ | – | – | 6 (2.4) | 5 (6.5) | 1 (2.9) | – | 1 (1.9) | 4 (2.3) | 6 (4.0) |
| Extramedullary plasmacytoma; *N* (%) | No | 770 (99.2) | 21 (100.0) | – | – | 250 (98.8) | 77 (100.0) | 33 (97.1) | – | 51 (96.2) | 176 (100.0) | 149 (100.0) |
|  | Yes | 6 (0.8) | 0 (0.0) | – | – | 3 (1.2) | 0 (0.0) | 1 (2.9) | – | 2 (3.8) | 0 (0.0) | 0 (0.0) |
| Prior PI exposure; *N* (%) | No | 97 (12.5) | 9 (42.9) | – | – | 15 (5.9) | 7 (9.1) | 3 (8.8) | – | 7 (13.2) | 29 (16.5) | 24 (16.1) |
|  | Yes | 679 (87.5) | 12 (57.1) | – | – | 238 (94.1) | 70 (90.9) | 31 (91.2) | – | 46 (86.8) | 147 (83.5) | 125 (83.9) |
| Prior IMID exposure; *N* (%) | No | – | – | – | – | – | – | – | – | – | – | – |
|  | Yes | 776 (100.0) | 21 (100.0) | – | – | 253 (100.0) | 77 (100.0) | 34 (100.0) | – | 53 (100.0) | 176 (100.0) | 149 (100.0) |
| Prior anti-CD38 mAb exposure; *N* (%) | No | 730 (94.1) | ^a^ | – | – | 253 (100.0) | 76 (98.7) | 34 (100.0) | – | 50 (94.3) | 142 (80.7) | 144 (96.6) |
|  | Yes | 46 (5.9) | ^a^ | – | – | 0 (0.0) | 1 (1.3) | 0 (0.0) | – | 3 (5.7) | 34 (19.3) | 5 (3.4) |
| Prior lenalidomide exposure; *N* (%) | No | – | – | – | – | – | – | – | – | – | – | – |
|  | Yes | 776 (100.0) | 21 (100.0) | – | – | 253 (100.0) | 77 (100.0) | 34 (100.0) | – | 53 (100.0) | 176 (100.0) | 149 (100.0) |
| Lenalidomide refractory; *N* (%) | No | – | – | – | – | – | – | – | – | – | – | – |
|  | Yes | 776 (100.0) | 21 (100.0) | – | – | 253 (100.0) | 77 (100.0) | 34 (100.0) | – | 53 (100.0) | 176 (100.0) | 149 (100.0) |
| Available follow-up from MM diagnosis; median (IQR) | Months | 48.1 (33.4 to 69.8) | 44.7 (36.8 to 69.6) | – | – | 60.7 (44.5 to 82.2) | 35.2 (27.9 to 46.5) | 54.0 (41.6 to 64.6) | – | 44.3 (30.4 to 54.6) | 44.9 (30.5 to 66.6) | 41.0 (28.9 to 63.0) |
| Available follow-up from start of line; median (IQR) | Months | 7.9 (3.1 to 18.6) | 13.9 (6.6 to 22.9) | – | – | 11.1 (3.2 to 24.9) | 5.6 (3.1 to 11.0) | 13.4 (5.6 to 29.3) | – | 3.9 (2.0 to 12.9) | 6.1 (2.5 to 13.0) | 7.8 (3.6 to 17.7) |

Data on patients treated with DaraVd at 2L can be found in the main results. A very small cohort of patients received Kd, KRd and PanoVd – data and statistics not reported for this group. Presence of ASCT (OPCS-4 code: X334) sourced from National Cancer Registration Dataset, HES APC and OP datasets between date of diagnosis with MM and start of 3L. Presence of lytic bone lesions (ICD-10 codes: M895, M899) sourced from HES APC and OP datasets between date of diagnosis with MM and start of 3L. Extramedullary plasmacytoma (ICD-10 code: C902) sourced from National Cancer Registration Dataset, HES APC and OP datasets between date of diagnosis with MM and start of 3L.

Abbreviations: 3L, third-line; ASCT, autologous stem cell transplant; D8, DREAMM-8; DaraVd, daratumumab–bortezomib–dexamethasone; ECOG, Eastern Cooperative Oncology Group; HES APC, hospital episode statistics admitted patient care; ICD-10, International Classification of Diseases, Tenth Revision; IMID, immunomodulatory drugs; IQR, interquartile range; IxaRd, ixazomib–lenalidomide–dexamethasone; Kd, carfilzomib–dexamethasone; KRd, carfilzomib–lenalidomide–dexamethasone; MM, multiple myeloma; OP, outpatient; OPCS-4, Office of Population Censuses and Surveys Classification of Surgical Operations and Procedures, Fourth Revision; PanoVd, panobinostat–bortezomib–dexamethasone; PI, proteasome inhibitor; PomDex, pomalidomide–dexamethasone; Rd, lenalidomide–dexamethasone; SD, standard deviation; Vd, bortezomib–dexamethasone.

^a^Small cohort–data not reported for this group.
^b^Stage at start of 3L is recorded based on the International Staging System.

TABLE S10. Prior treatments of patients treated at 3L by regimen and drug class in the D8-like cohort

| **Line regimen/class** | | **All D8-like at 3L** | **Bortezomib** | **Daratumumab** | **DaraVd** | **IxaRd** | **Rd** | **PanoVd** | **PomDex** | **Other** |
| --- | --- | --- | --- | --- | --- | --- | --- | --- | --- | --- |
| Total number of patient lines | | 1344 (100.0) | 36 (2.7) | 423 (31.5) | 124 (9.2) | 74 (5.5) | 48 (3.6) | 84 (6.2) | 304 (22.6) | 241 (17.9) |
| 1L regimen | Bortezomib–cyclophosphamide | 402 (29.9) | 6 (16.7) | 145 (34.3) | 34 (27.4) | 26 (35.1) | 9 (18.8) | 26 (31.0) | 79 (26.0) | 76 (31.5) |
|  | Bortezomib | 241 (17.9) | 5 (13.9) | 102 (24.1) | 4 (3.2) | 14 (18.9) | 2 (4.2) | 18 (21.4) | 54 (17.8) | 42 (17.4) |
|  | Bortezomib–thalidomide | 211 (15.7) | 2 (5.6) | 60 (14.2) | 52 (41.9) | 11 (14.9) | 11 (22.9) | 15 (17.9) | 33 (10.9) | 23 (9.5) |
|  | Bortezomib–melphalan | 108 (8.0) | 1 (2.8) | 30 (7.1) | 4 (3.2) | 1 (1.4) | – | 5 (6.0) | 40 (13.2) | 27 (11.2) |
|  | Cyclophosphamide–thalidomide | 108 (8.0) | 11 (30.6) | 33 (7.8) | 6 (4.8) | 8 (10.8) | 1 (2.1) | 4 (4.8) | 18 (5.9) | 25 (10.4) |
|  | Lenalidomide | 101 (7.5) | 4 (11.1) | 15 (3.5) | 7 (5.6) | 6 (8.1) | 8 (16.7) | 6 (7.1) | 42 (13.8) | 13 (5.4) |
|  | Cyclophosphamide–lenalidomide | 45 (3.3) | 2 (5.6) | 8 (1.9) | 3 (2.4) | 3 (4.1) | 9 (18.8) | 1 (1.2) | 8 (2.6) | 10 (4.1) |
|  | Ixazomib–lenalidomide | 25 (1.9) | – | 1 (0.2) | 1 (0.8) | – | 1 (2.1) | 4 (4.8) | 13 (4.3) | 5 (2.1) |
|  | Melphalan–thalidomide | 13 (1.0) | 4 (11.1) | 2 (0.5) | – | 2 (2.7) | – | 1 (1.2) | 3 (1.0) | 1 (0.4) |
|  | Thalidomide | 12 (0.9) | – | 3 (0.7) | 1 (0.8) | 1 (1.4) | 3 (6.2) | – | – | 4 (1.7) |
|  | Bortezomib–cyclophosphamide–thalidomide | 11 (0.8) | – | 2 (0.5) | 4 (3.2) | – | 2 (4.2) | 1 (1.2) | 1 (0.3) | 1 (0.4) |
|  | Carfilzomib | 11 (0.8) | – | 7 (1.7) | 1 (0.8) | – | – | – | 2 (0.7) | 1 (0.4) |
|  | Melphalan | 10 (0.7) | – | 7 (1.7) | 1 (0.8) | – | – | – | 2 (0.7) | – |
|  | Bortezomib–melphalan–thalidomide | 8 (0.6) | – | – | 1 (0.8) | 2 (2.7) | – | – | 2 (0.7) | 2 (0.8) |
|  | Bortezomib–cyclophosphamide–melphalan–thalidomide | 5 (0.4) | – | 3 (0.7) | 2 (1.6) | – | – | – | – | – |
|  | Bortezomib–doxorubicin | 5 (0.4) | – | 1 (0.2) | – | – | – | 1 (1.2) | – | 3 (1.2) |
|  | Bortezomib–cisplatin–cyclophosphamide–doxorubicin–etoposide–thalidomide | 3 (0.2) | – | – | – | – | – | – | – | 2 (0.8) |
|  | Bortezomib–cyclophosphamide–melphalan | 3 (0.2) | – | 1 (0.2) | – | – | – | – | 1 (0.3) | 1 (0.4) |
|  | Carfilzomib–cyclophosphamide | 3 (0.2) | – | 1 (0.2) | – | – | – | 1 (1.2) | – | 1 (0.4) |
|  | Cyclophosphamide | 3 (0.2) | – | 1 (0.2) | – | – | – | 1 (1.2) | 1 (0.3) | – |
|  | Daratumumab | 3 (0.2) | – | – | – | – | – | – | 2 (0.7) | 1 (0.4) |
|  | Bortezomib–daratumumab | 2 (0.1) | – | – | – | – | – | – | 2 (0.7) | – |
|  | Cyclophosphamide–melphalan | 2 (0.1) | – | – | 1 (0.8) | – | – | – | – | 1 (0.4) |
|  | Cyclophosphamide–melphalan–thalidomide | 2 (0.1) | – | – | 1 (0.8) | – | 1 (2.1) | – | – | – |
|  | Bendamustine | 1 (0.1) | – | – | – | – | – | – | – | 1 (0.4) |
|  | Bendamustine–melphalan–thalidomide | 1 (0.1) | – | – | 1 (0.8) | – | – | – | – | – |
|  | Bortezomib–cyclophosphamide–daratumumab–melphalan | 1 (0.1) | – | – | – | – | – | – | 1 (0.3) | – |
|  | Bortezomib–cyclophosphamide–lenalidomide | 1 (0.1) | – | 1 (0.2) | – | – | – | – | – | – |
|  | Bortezomib–lenalidomide | 1 (0.1) | 1 (2.8) | – | – | – | – | – | – | – |
|  | Cyclophosphamide–docetaxel–epirubicin | 1 (0.1) | – | – | – | – | 1 (2.1) | – | – | – |
|  | Cyclophosphamide–fludarabine | 1 (0.1) | – | – | – | – | – | – | – | 1 (0.4) |
| 1L drug class | Chemotherapies–PI | 521 (38.8) | 7 (19.4) | 178 (42.1) | 38 (30.6) | 27 (36.5) | 9 (18.8) | 33 (39.3) | 120 (39.5) | 108 (44.8) |
|  | PI | 252 (18.8) | 5 (13.9) | 109 (25.8) | 5 (4.0) | 14 (18.9) | 2 (4.2) | 18 (21.4) | 56 (18.4) | 43 (17.8) |
|  | IMID–PI | 237 (17.6) | 3 (8.3) | 61 (14.4) | 53 (42.7) | 11 (14.9) | 12 (25.0) | 19 (22.6) | 46 (15.1) | 28 (11.6) |
|  | Chemotherapies–IMID | 169 (12.6) | 17 (47.2) | 43 (10.2) | 11 (8.9) | 13 (17.6) | 11 (22.9) | 6 (7.1) | 29 (9.5) | 36 (14.9) |
|  | IMID | 113 (8.4) | 4 (11.1) | 18 (4.3) | 8 (6.5) | 7 (9.5) | 11 (22.9) | 6 (7.1) | 42 (13.8) | 17 (7.1) |
|  | Chemotherapies–IMID–PI | 28 (2.1) | – | 6 (1.4) | 7 (5.6) | 2 (2.7) | 2 (4.2) | 1 (1.2) | 3 (1.0) | 5 (2.1) |
|  | Chemotherapies | 18 (1.3) | – | 8 (1.9) | 2 (1.6) | – | 1 (2.1) | 1 (1.2) | 3 (1.0) | 3 (1.2) |
|  | Anti-CD38 mAb | 3 (0.2) | – | – | – | – | – | – | 2 (0.7) | 1 (0.4) |
|  | Anti-CD38 mAb–PI | 2 (0.1) | – | – | – | – | – | – | 2 (0.7) | – |
|  | Anti-CD38 mAb–chemotherapies–PI | 1 (0.1) | – | – | – | – | – | – | 1 (0.3) | – |
| 2L regimen | Lenalidomide | 717 (53.3) | 26 (72.2) | 193 (45.6) | 69 (55.6) | 47 (63.5) | – | 50 (59.5) | 166 (54.6) | 159 (66.0) |
|  | Ixazomib–lenalidomide | 327 (24.3) | 5 (13.9) | 165 (39.0) | 46 (37.1) | 3 (4.1) | 12 (25.0) | 8 (9.5) | 53 (17.4) | 35 (14.5) |
|  | Cyclophosphamide–lenalidomide | 124 (9.2) | 2 (5.6) | 45 (10.6) | 3 (2.4) | 8 (10.8) | 16 (33.3) | 11 (13.1) | 20 (6.6) | 19 (7.9) |
|  | Daratumumab | 56 (4.2) | 2 (5.6) | – | 1 (0.8) | 1 (1.4) | – | 5 (6.0) | 39 (12.8) | 8 (3.3) |
|  | Bortezomib–cyclophosphamide | 20 (1.5) | – | 4 (0.9) | 1 (0.8) | 2 (2.7) | 4 (8.3) | – | 7 (2.3) | 2 (0.8) |
|  | Bortezomib | 13 (1.0) | – | 3 (0.7) | – | 2 (2.7) | 3 (6.2) | – | 3 (1.0) | 2 (0.8) |
|  | Bortezomib–daratumumab | 11 (0.8) | – | – | – | – | 3 (6.2) | 1 (1.2) | 4 (1.3) | 3 (1.2) |
|  | Carfilzomib–lenalidomide | 9 (0.7) | – | – | – | 2 (2.7) | 1 (2.1) | 2 (2.4) | – | 2 (0.8) |
|  | Melphalan | 8 (0.6) | – | 1 (0.2) | 1 (0.8) | – | 5 (10.4) | – | – | 1 (0.4) |
|  | Carfilzomib | 5 (0.4) | – | – | – | 1 (1.4) | – | 1 (1.2) | 1 (0.3) | 2 (0.8) |
|  | Cyclophosphamide–thalidomide | 5 (0.4) | – | 2 (0.5) | – | 1 (1.4) | 1 (2.1) | 1 (1.2) | – | – |
|  | Bendamustine–thalidomide | 4 (0.3) | – | – | – | 1 (1.4) | – | 1 (1.2) | 1 (0.3) | 1 (0.4) |
|  | Bortezomib–thalidomide | 4 (0.3) | – | 1 (0.2) | 1 (0.8) | – | 1 (2.1) | – | 1 (0.3) | – |
|  | Cyclophosphamide–lenalidomide–thalidomide | 4 (0.3) | – | 1 (0.2) | – | – | – | 1 (1.2) | 1 (0.3) | 1 (0.4) |
|  | Lenalidomide–melphalan | 4 (0.3) | – | – | 1 (0.8) | – | 2 (4.2) | – | 1 (0.3) | – |
|  | Bortezomib–melphalan | 3 (0.2) | – | 1 (0.2) | 1 (0.8) | – | – | – | – | 1 (0.4) |
|  | Cyclophosphamide–ixazomib–lenalidomide | 3 (0.2) | – | – | – | 2 (2.7) | – | – | 1 (0.3) | – |
|  | Cyclophosphamide–ixazomib–lenalidomide–thalidomide | 3 (0.2) | – | 1 (0.2) | – | – | – | 1 (1.2) | – | 1 (0.4) |
|  | Daratumumab–lenalidomide | 3 (0.2) | – | – | – | – | – | – | 3 (1.0) | – |
|  | Bortezomib–cisplatin–cyclophosphamide–doxorubicin–etoposide–thalidomide | 2 (0.1) | – | 1 (0.2) | – | – | – | – | – | 1 (0.4) |
|  | Bortezomib–panobinostat | 2 (0.1) | – | 1 (0.2) | – | – | – | – | 1 (0.3) | – |
|  | Ixazomib–lenalidomide–melphalan | 2 (0.1) | – | – | – | 2 (2.7) | – | – | – | – |
|  | Melphalan–thalidomide | 2 (0.1) | 1 (2.8) | 1 (0.2) | – | – | – | – | – | – |
|  | Bendamustine | 1 (0.1) | – | – | – | – | – | 1 (1.2) | – | – |
|  | Bortezomib–cyclophosphamide–lenalidomide | 1 (0.1) | – | 1 (0.2) | – | – | – | – | – | – |
|  | Bortezomib–doxorubicin | 1 (0.1) | – | – | – | – | – | 1 (1.2) | – | – |
|  | Carfilzomib–daratumumab | 1 (0.1) | – | 1 (0.2) | – | – | – | – | – | – |
|  | Cisplatin–cyclophosphamide–doxorubicin–etoposide–ixazomib–lenalidomide–thalidomide | 1 (0.1) | – | – | – | – | – | – | – | 1 (0.4) |
|  | Cyclophosphamide | 1 (0.1) | – | – | – | – | – | – | – | 1 (0.4) |
|  | Cyclophosphamide–daratumumab–doxorubicin rituximab–vincristine | 1 (0.1) | – | – | – | – | – | – | 1 (0.3) | – |
|  | Cyclophosphamide–daratumumab–lenalidomide | 1 (0.1) | – | – | – | – | – | – | 1 (0.3) | – |
|  | Cyclophosphamide–lenalidomide–melphalan | 1 (0.1) | – | – | – | 1 (1.4) | – | – | – | – |
|  | Ixazomib | 1 (0.1) | – | – | – | 1 (1.4) | – | – | – | – |
|  | Ixazomib–lenalidomide–melphalan–thalidomide | 1 (0.1) | – | 1 (0.2) | – | – | – | – | – | – |
|  | Ixazomib–melphalan–thalidomide | 1 (0.1) | – | – | – | – | – | – | – | – |
|  | Thalidomide | 1 (0.1) | – | – | – | – | – | – | – | 1 (0.4) |
| 2L drug class | IMID | 718 (53.4) | 26 (72.2) | 193 (45.6) | 69 (55.6) | 47 (63.5) | – | 50 (59.5) | 166 (54.6) | 160 (66.4) |
|  | IMID–PI | 340 (25.3) | 5 (13.9) | 166 (39.2) | 47 (37.9) | 5 (6.8) | 14 (29.2) | 10 (11.9) | 54 (17.8) | 37 (15.4) |
|  | Chemotherapies–IMID | 144 (10.7) | 3 (8.3) | 49 (11.6) | 4 (3.2) | 11 (14.9) | 19 (39.6) | 14 (16.7) | 23 (7.6) | 21 (8.7) |
|  | Anti-CD38 mAb | 56 (4.2) | 2 (5.6) | – | 1 (0.8) | 1 (1.4) | – | 5 (6.0) | 39 (12.8) | 8 (3.3) |
|  | Chemotherapies–PI | 24 (1.8) | – | 5 (1.2) | 2 (1.6) | 2 (2.7) | 4 (8.3) | 1 (1.2) | 7 (2.3) | 3 (1.2) |
|  | PI | 19 (1.4) | – | 3 (0.7) | – | 4 (5.4) | 3 (6.2) | 1 (1.2) | 4 (1.3) | 4 (1.7) |
|  | Chemotherapies–IMID–PI | 14 (1.0) | – | 4 (0.9) | – | 4 (5.4) | – | 1 (1.2) | 1 (0.3) | 3 (1.2) |
|  | Anti-CD38 mAb–PI | 12 (0.9) | – | 1 (0.2) | – | – | 3 (6.2) | 1 (1.2) | 4 (1.3) | 3 (1.2) |
|  | Chemotherapies | 10 (0.7) | – | 1 (0.2) | 1 (0.8) | – | 5 (10.4) | 1 (1.2) | – | 2 (0.8) |
|  | Anti-CD38 mAb–IMID | 3 (0.2) | – | – | – | – | – | – | 3 (1.0) | – |
|  | HDAC–PI | 2 (0.1) | – | 1 (0.2) | – | – | – | – | 1 (0.3) | – |
|  | Anti-CD38 mAb–Chemotherapies–mAb | 1 (0.1) | – | – | – | – | – | – | 1 (0.3) | – |
|  | Anti-CD38 mAb–chemotherapies–IMID | 1 (0.1) | – | – | – | – | – | – | 1 (0.3) | – |
| **Line regimen/class** | | **Lenalidomide-refractory D8-like at 3L** | **Bortezomib** | **Daratumumab** | **DaraVd** | **IxaRd** | **Rd** | **PanoVd** | **PomDex** | **Other** |
|  |  |  |  |  |  |  |  |  |  |  |
| Total number of patient lines | | 776 (100.0) | 21 (2.7) | 253 (32.6) | 77 (9.9) | 34 (4.4) | 0 (0) | 53 (6.8) | 176 (22.7) | 149 (19.2) |
| 1L regimen | Bortezomib–cyclophosphamide | 239 (30.8) | 4 (19.0) | 92 (36.4) | 21 (27.3) | 13 (38.2) | – | 16 (30.2) | 47 (26.7) | 46 (30.9) |
|  | Bortezomib–thalidomide | 140 (18.0) | 2 (9.5) | 42 (16.6) | 36 (46.8) | 6 (17.6) | – | 12 (22.6) | 21 (11.9) | 20 (13.4) |
|  | Bortezomib | 124 (16.0) | 2 (9.5) | 48 (19.0) | 2 (2.6) | 8 (23.5) | – | 9 (17.0) | 27 (15.3) | 27 (18.1) |
|  | Bortezomib–melphalan | 60 (7.7) | 1 (4.8) | 16 (6.3) | 1 (1.3) | 1 (2.9) | – | 4 (7.5) | 22 (12.5) | 15 (10.1) |
|  | Cyclophosphamide–thalidomide | 54 (7.0) | 7 (33.3) | 16 (6.3) | 3 (3.9) | 1 (2.9) | – | 3 (5.7) | 8 (4.5) | 16 (10.7) |
|  | Lenalidomide | 52 (6.7) | 2 (9.5) | 10 (4.0) | 6 (7.8) | 1 (2.9) | – | 2 (3.8) | 24 (13.6) | 3 (2.0) |
|  | Cyclophosphamide–lenalidomide | 22 (2.8) | – | 5 (2.0) | 1 (1.3) | 1 (2.9) | – | 1 (1.9) | 5 (2.8) | 4 (2.7) |
|  | Ixazomib–lenalidomide | 20 (2.6) | – | 1 (0.4) | 1 (1.3) | – | – | 2 (3.8) | 10 (5.7) | 5 (3.4) |
|  | Melphalan–thalidomide | 10 (1.3) | 2 (9.5) | 1 (0.4) | – | 2 (5.9) | – | 1 (1.9) | 3 (1.7) | 1 (0.7) |
|  | Carfilzomib | 9 (1.2) | – | 7 (2.8) | – | – | – | – | 1 (0.6) | 1 (0.7) |
|  | Bortezomib–melphalan–thalidomide | 6 (0.8) | – | – | 1 (1.3) | 1 (2.9) | – | – | 2 (1.1) | 1 (0.7) |
|  | Melphalan | 6 (0.8) | – | 5 (2.0) | – | – | – | – | 1 (0.6) | – |
|  | Thalidomide | 6 (0.8) | – | 3 (1.2) | – | – | – | – | – | 3 (2.0) |
|  | Bortezomib–cyclophosphamide–melphalan–thalidomide | 5 (0.6) | – | 3 (1.2) | 2 (2.6) | – | – | – | – | – |
|  | Bortezomib–cyclophosphamide–thalidomide | 5 (0.6) | – | 1 (0.4) | 1 (1.3) | – | – | 1 (1.9) | 1 (0.6) | 1 (0.7) |
|  | Carfilzomib–cyclophosphamide | 3 (0.4) | – | 1 (0.4) | – | – | – | 1 (1.9) | – | 1 (0.7) |
|  | Bortezomib–cisplatin–cyclophosphamide–doxorubicin–etoposide–thalidomide | 2 (0.3) | – | – | – | – | – | – | – | 2 (1.3) |
|  | Bortezomib–cyclophosphamide–melphalan | 2 (0.3) | – | 1 (0.4) | – | – | – | – | 1 (0.6) | – |
|  | Bortezomib–doxorubicin | 2 (0.3) | – | – | – | – | – | – | – | 2 (1.3) |
|  | Cyclophosphamide–melphalan | 2 (0.3) | – | – | 1 (1.3) | – | – | – | – | 1 (0.7) |
|  | Daratumumab | 2 (0.3) | – | – | – | – | – | – | 2 (1.1) | – |
|  | Bendamustine–melphalan–thalidomide | 1 (0.1) | – | – | 1 (1.3) | – | – | – | – | – |
|  | Bortezomib–cyclophosphamide– daratumumab–melphalan | 1 (0.1) | – | – | – | – | – | – | 1 (0.6) | – |
|  | Bortezomib–cyclophosphamide–lenalidomide | 1 (0.1) | – | 1 (0.4) | – | – | – | – | – | – |
|  | Bortezomib–lenalidomide | 1 (0.1) | 1 (4.8) | – | – | – | – | – | – | – |
|  | Cyclophosphamide | 1 (0.1) | – | – | – | – | – | 1 (1.9) | – | – |
| 1L drug class | Chemotherapies–PI | 306 (39.4) | 5 (23.8) | 110 (43.5) | 22 (28.6) | 14 (41.2) | – | 21 (39.6) | 70 (39.8) | 64 (43.0) |
|  | IMID–PI | 161 (20.7) | 3 (14.3) | 43 (17.0) | 37 (48.1) | 6 (17.6) | – | 14 (26.4) | 31 (17.6) | 25 (16.8) |
|  | PI | 133 (17.1) | 2 (9.5) | 55 (21.7) | 2 (2.6) | 8 (23.5) | – | 9 (17.0) | 28 (15.9) | 28 (18.8) |
|  | Chemotherapies–IMID | 87 (11.2) | 9 (42.9) | 22 (8.7) | 5 (6.5) | 4 (11.8) | – | 5 (9.4) | 16 (9.1) | 21 (14.1) |
|  | IMID | 58 (7.5) | 2 (9.5) | 13 (5.1) | 6 (7.8) | 1 (2.9) | – | 2 (3.8) | 24 (13.6) | 6 (4.0) |
|  | Chemotherapies–IMID–PI | 19 (2.4) | – | 5 (2.0) | 4 (5.2) | 1 (2.9) | – | 1 (1.9) | 3 (1.7) | 4 (2.7) |
|  | Chemotherapies | 9 (1.2) | – | 5 (2.0) | 1 (1.3) | – | – | 1 (1.9) | 1 (0.6) | 1 (0.7) |
|  | Anti-CD38 mAb | 2 (0.3) | – | – | – | – | – | – | 2 (1.1) | – |
|  | Anti-CD38 mAb–chemotherapies–PI | 1 (0.1) | – | – | – | – | – | – | 1 (0.6) | – |
|  |  | 2L | | | | | | | | |
| 2L regimen | Lenalidomide | 407 (52.4) | 14 (66.7) | 101 (39.9) | 45 (58.4) | 28 (82.4) | – | 33 (62.3) | 92 (52.3) | 93 (62.4) |
|  | Ixazomib–lenalidomide | 218 (28.1) | 5 (23.8) | 120 (47.4) | 26 (33.8) | – | – | 5 (9.4) | 34 (19.3) | 27 (18.1) |
|  | Cyclophosphamide–lenalidomide | 58 (7.5) | 1 (4.8) | 22 (8.7) | 2 (2.6) | 1 (2.9) | – | 6 (11.3) | 10 (5.7) | 15 (10.1) |
|  | Daratumumab | 33 (4.3) | 1 (4.8) | – | 1 (1.3) | – | – | 3 (5.7) | 23 (13.1) | 5 (3.4) |
|  | Bortezomib | 8 (1.0) | – | 3 (1.2) | – | – | – | – | 3 (1.7) | 1 (0.7) |
|  | Bortezomib–cyclophosphamide | 8 (1.0) | – | 1 (0.4) | – | – | – | – | 4 (2.3) | – |
|  | Bortezomib–daratumumab | 5 (0.6) | – | – | – | – | – | – | 3 (1.7) | – |
|  | Carfilzomib–lenalidomide | 5 (0.6) | – | – | – | – | – | 2 (3.8) | – | 2 (1.3) |
|  | Cyclophosphamide–lenalidomide–thalidomide | 3 (0.4) | – | – | – | – | – | 1 (1.9) | 1 (0.6) | 1 (0.7) |
|  | Cyclophosphamide–thalidomide | 3 (0.4) | – | 1 (0.4) | – | 1 (2.9) | – | – | – | – |
|  | Daratumumab–lenalidomide | 3 (0.4) | – | – | – | – | – | – | 3 (1.7) | – |
|  | Melphalan | 3 (0.4) | – | – | 1 (1.3) | – | – | – | – | – |
|  | Bendamustine–thalidomide | 2 (0.3) | – | – | – | – | – | 1 (1.9) | – | 1 (0.7) |
|  | Bortezomib–melphalan | 2 (0.3) | – | 1 (0.4) | 1 (1.3) | – | – | – | – | – |
|  | Cyclophosphamide–ixazomib–lenalidomide | 2 (0.3) | – | – | – | 1 (2.9) | – | – | 1 (0.6) | – |
|  | Cyclophosphamide–ixazomib–lenalidomide–thalidomide | 2 (0.3) | – | – | – | – | – | 1 (1.9) | – | 1 (0.7) |
|  | Ixazomib–lenalidomide–melphalan | 2 (0.3) | – | – | – | 2 (5.9) | – | – | – | – |
|  | Bendamustine | 1 (0.1) | – | – | – | – | – | 1 (1.9) | – | – |
|  | Bortezomib–cisplatin–cyclophosphamide–doxorubicin–etoposide–thalidomide | 1 (0.1) | – | 1 (0.4) | – | – | – | – | – | – |
|  | Bortezomib–panobinostat | 1 (0.1) | – | 1 (0.4) | – | – | – | – | – | – |
|  | Bortezomib–thalidomide | 1 (0.1) | – | – | 1 (1.3) | – | – | – | – | – |
|  | Cisplatin–cyclophosphamide–doxorubicin–etoposide–ixazomib–lenalidomide–thalidomide | 1 (0.1) | – | – | – | – | – | – | – | 1 (0.7) |
|  | Cyclophosphamide | 1 (0.1) | – | – | – | – | – | – | – | 1 (0.7) |
|  | Cyclophosphamide–daratumumab–doxorubicin–rituximab–vincristine | 1 (0.1) | – | – | – | – | – | – | 1 (0.6) | – |
|  | Cyclophosphamide–daratumumab–lenalidomide | 1 (0.1) | – | – | – | – | – | – | 1 (0.6) | – |
|  | Ixazomib | 1 (0.1) | – | – | – | 1 (2.9) | – | – | – | – |
|  | Ixazomib–lenalidomide–melphalan–thalidomide | 1 (0.1) | – | 1 (0.4) | – | – | – | – | – | – |
|  | Melphalan–thalidomide | 1 (0.1) | – | 1 (0.4) | – | – | – | – | – | – |
|  | Thalidomide | 1 (0.1) | – | – | – | – | – | – | – | 1 (0.7) |
| 2L drug class | IMID | 408 (52.6) | 14 (66.7) | 101 (39.9) | 45 (58.4) | 28 (82.4) | – | 33 (62.3) | 92 (52.3) | 94 (63.1) |
|  | IMID–PI | 224 (28.9) | 5 (23.8) | 120 (47.4) | 27 (35.1) | – | – | 7 (13.2) | 34 (19.3) | 29 (19.5) |
|  | Chemotherapies–IMID | 67 (8.6) | 1 (4.8) | 24 (9.5) | 2 (2.6) | 2 (5.9) | – | 8 (15.1) | 11 (6.2) | 17 (11.4) |
|  | Anti-CD38 mAb | 33 (4.3) | 1 (4.8) | – | 1 (1.3) | – | – | 3 (5.7) | 23 (13.1) | 5 (3.4) |
|  | Chemotherapies–PI | 10 (1.3) | – | 2 (0.8) | 1 (1.3) | – | – | – | 4 (2.3) | – |
|  | Chemotherapies–IMID–PI | 9 (1.2) | – | 2 (0.8) | – | 3 (8.8) | – | 1 (1.9) | 1 (0.6) | 2 (1.3) |
|  | PI | 9 (1.2) | – | 3 (1.2) | – | 1 (2.9) | – | – | 3 (1.7) | 1 (0.7) |
|  | Anti-CD38 mAb–PI | 5 (0.6) | – | – | – | – | – | – | 3 (1.7) | – |
|  | Chemotherapies | 5 (0.6) | – | – | 1 (1.3) | – | – | 1 (1.9) | – | 1 (0.7) |
|  | Anti-CD38 mAb–IMID | 3 (0.4) | – | – | – | – | – | – | 3 (1.7) | – |
|  | Anti-CD38 mAb–chemotherapies–mAb | 1 (0.1) | – | – | – | – | – | – | 1 (0.6) | – |
|  | Anti-CD38 mAb–chemotherapies–IMID | 1 (0.1) | – | – | – | – | – | – | 1 (0.6) | – |
|  | HDAC–PI | 1 (0.1) | – | 1 (0.4) | – | – | – | – | – | – |

For the D8-like cohort, data for index lines Kd, KRd, IxaRd and PanoVd not reported due to small group size. IMID: lenalidomide, pomalidomide, thalidomide; PI: bortezomib, carfilzomib, ixazomib; mAb: rituximab.

Abbreviations: 1L, first-line; 2L, second-line; 3L, third-line; D8, DREAMM-8; DaraVd, daratumumab–bortezomib–dexamethasone; HDAC, histone deacetylase inhibitor; IMID, immunomodulatory drugs; IxaRd, ixazomib–lenalidomide–dexamethasone; Kd, carfilzomib–dexamethasone; KRd, carfilzomib–lenalidomide–dexamethasone; mAb, monoclonal antibody; PanoVd, panobinostat–bortezomib–dexamethasone; PI, proteasome inhibitor; PomDex, pomalidomide–dexamethasone; Rd, lenalidomide–dexamethasone.

TABLE S11. Subsequent treatment regimens and drug classes for patients at 2L or 3L in the D8-like cohort

| **Lenalidomide-exposed** | | **4L** | **5L+** |
| --- | --- | --- | --- |
| Regimen | Pomalidomide | 254 (40.1) | 82 (27.2) |
|  | Daratumumab | 137 (21.6) | 12 (4.0) |
|  | Bortezomib–panobinostat | 86 (13.6) | 77 (25.5) |
|  | Belantamab mafodotin | 4 (0.6) | 19 (6.3) |
|  | Cyclophosphamide–pomalidomide | 14 (2.2) | 19 (6.3) |
|  | Melphalan–thalidomide | 10 (1.6) | 17 (5.6) |
|  | Bortezomib–daratumumab | 15 (2.4) | 1 (0.3) |
|  | Cyclophosphamide–thalidomide | 15 (2.4) | 11 (3.6) |
|  | Ixazomib–lenalidomide | 14 (2.2) | 1 (0.3) |
|  | Lenalidomide | 13 (2.1) | 3 (1.0) |
|  | Bendamustine–thalidomide | 7 (1.1) | 11 (3.6) |
|  | Bendamustine | 7 (1.1) | 9 (3.0) |
|  | Isatuximab–pomalidomide | 7 (1.1) | – |
|  | Cisplatin–cyclophosphamide–doxorubicin–etoposide–thalidomide | 6 (0.9) | 4 (1.3) |
|  | Cyclophosphamide | 3 (0.5) | 6 (2.0) |
|  | Thalidomide | 6 (0.9) | 4 (1.3) |
|  | Bortezomib | 5 (0.8) | 4 (1.3) |
|  | Bortezomib–cisplatin–cyclophosphamide–doxorubicin–etoposide–thalidomide | 5 (0.8) | 1 (0.3) |
|  | Bortezomib–cyclophosphamide | 4 (0.6) | 3 (1.0) |
|  | Bortezomib–panobinostat–thalidomide | 2 (0.3) | 4 (1.3) |
|  | Melphalan | 4 (0.6) | 4 (1.3) |
|  | Bortezomib–melphalan | 3 (0.5) | – |
|  | Cyclophosphamide–doxorubicin–rituximab–vincristine | – | 2 (0.7) |
|  | Bendamustine–rituximab | – | 1 (0.3) |
|  | Bortezomib–cyclophosphamide–lenalidomide | 1 (0.2) | – |
|  | Bortezomib–daratumumab–panobinostat | 1 (0.2) | – |
|  | Bortezomib–daratumumab–thalidomide | 1 (0.2) | – |
|  | Bortezomib–panobinostat–pomalidomide–thalidomide | – | 1 (0.3) |
|  | Bortezomib–thalidomide | – | 1 (0.3) |
|  | Carfilzomib | – | 1 (0.3) |
|  | Carfilzomib–cyclophosphamide | 1 (0.2) | 1 (0.3) |
|  | Carfilzomib–lenalidomide | 1 (0.2) | – |
|  | Carfilzomib–pomalidomide | 1 (0.2) | – |
|  | Cisplatin–cyclophosphamide–doxorubicin–etoposide–pomalidomide–thalidomide | 1 (0.2) | – |
|  | Cyclophosphamide–cytarabine–pomalidomide | 1 (0.2) | – |
|  | Cyclophosphamide–daratumumab–pomalidomide | – | 1 (0.3) |
|  | Cyclophosphamide–doxorubicin–vincristine | 1 (0.2) | – |
|  | Cyclophosphamide–fludarabine | 1 (0.2) | – |
|  | Cyclophosphamide–lenalidomide | 1 (0.2) | – |
|  | Etoposide–idarubicin–thalidomide | – | 1 (0.3) |
|  | Ixazomib | – | 1 (0.3) |
|  | Panobinostat | 1 (0.2) | – |
| Drug classes | IMID | 273 (43.1) | 89 (29.5) |
|  | Anti-CD38 mAb | 137 (21.6) | 12 (4.0) |
|  | HDAC–PI | 86 (13.6) | 77 (25.5) |
|  | Chemotherapies–IMID | 55 (8.7) | 63 (20.9) |
|  | Chemotherapies | 16 (2.5) | 20 (6.6) |
|  | BCMA | 4 (0.6) | 19 (6.3) |
|  | IMID–PI | 16 (2.5) | 2 (0.7) |
|  | Anti-CD38 mAb–PI | 15 (2.4) | 1 (0.3) |
|  | Chemotherapies–PI | 8 (1.3) | 4 (1.3) |
|  | Anti-CD38 mAb–IMID | 7 (1.1) | – |
|  | Chemotherapies–IMID–PI | 6 (0.9) | 1 (0.3) |
|  | PI | 5 (0.8) | 6 (2.0) |
|  | HDAC–IMID–PI | 2 (0.3) | 5 (1.7) |
|  | Chemotherapies–mAb | – | 2 (0.7) |
|  | Anti-CD38 mAb–chemotherapies–IMID | – | 1 (0.3) |
|  | Anti-CD38 mAb–HDAC–PI | 1 (0.2) | – |
|  | Anti-CD38 mAb–IMID–PI | 1 (0.2) | – |
|  | HDAC | 1 (0.2) | – |
| **Lenalidomide-refractory** | | **4L** | **5L+** |
| Regimen | Pomalidomide | 160 (41.0) | 51 (27.7) |
|  | Daratumumab | 84 (21.5) | 6 (3.3) |
|  | Bortezomib–panobinostat | 54 (13.8) | 52 (28.3) |
|  | Belantamab mafodotin | 2 (0.5) | 16 (8.7) |
|  | Bortezomib–daratumumab | 10 (2.6) | 1 (0.5) |
|  | Cyclophosphamide–pomalidomide | 9 (2.3) | 10 (5.4) |
|  | Ixazomib–lenalidomide | 8 (2.1) | – |
|  | Melphalan–thalidomide | 8 (2.1) | 8 (4.3) |
|  | Cyclophosphamide–thalidomide | 5 (1.3) | 7 (3.8) |
|  | Lenalidomide | 7 (1.8) | – |
|  | Bendamustine | 3 (0.8) | 6 (3.3) |
|  | Isatuximab–pomalidomide | 6 (1.5) | – |
|  | Bendamustine–thalidomide | 5 (1.3) | 5 (2.7) |
|  | Cisplatin–cyclophosphamide–doxorubicin–etoposide–thalidomide | 5 (1.3) | 1 (0.5) |
|  | Cyclophosphamide | 1 (0.3) | 4 (2.2) |
|  | Melphalan | 4 (1.0) | 2 (1.1) |
|  | Thalidomide | 4 (1.0) | 2 (1.1) |
|  | Bortezomib | 2 (0.5) | 3 (1.6) |
|  | Bortezomib–cyclophosphamide | 3 (0.8) | 3 (1.6) |
|  | Bortezomib–panobinostat–thalidomide | 2 (0.5) | 3 (1.6) |
|  | Bortezomib–cisplatin–cycliphosphamide–doxorubicin–etoposide–thalidomide | 2 (0.5) | – |
|  | Cyclophosphamide–doxorubicin–rituximab–vincristine | – | 2 (1.1) |
|  | Bortezomib–cyclophosphamide–lenalidomide | 1 (0.3) | – |
|  | Bortezomib–melphalan | 1 (0.3) | – |
|  | Carfilzomib–cyclophosphamide | 1 (0.3) | 1 (0.5) |
|  | Carfilzomib–pomalidomide | 1 (0.3) | – |
|  | Cisplatin–cyclophosphamide–doxorubicin–etoposide–pomalidomide–thalidomide | 1 (0.3) | – |
|  | Cyclophosphamide–cytarabine–pomalidomide | 1 (0.3) | – |
|  | Ixazomib | – | 1 (0.5) |
| Drug classes | IMID | 171 (43.8) | 53 (28.8) |
|  | Anti-CD38 mAb | 84 (21.5) | 6 (3.3) |
|  | HDAC–PI | 54 (13.8) | 52 (28.3) |
|  | Chemotherapies–IMID | 34 (8.7) | 31 (16.8) |
|  | BCMA | 2 (0.5) | 16 (8.7) |
|  | Chemotherapies | 8 (2.1) | 12 (6.5) |
|  | Anti-CD38 mAb–PI | 10 (2.6) | 1 (0.5) |
|  | IMID–PI | 9 (2.3) | – |
|  | Anti-CD38 mAb–IMID | 6 (1.5) | – |
|  | Chemotherapies–PI | 5 (1.3) | 4 (2.2) |
|  | PI | 2 (0.5) | 4 (2.2) |
|  | Chemotherapies–IMID–PI | 3 (0.8) | – |
|  | HDAC–IMID–PI | 2 (0.5) | 3 (1.6) |
|  | Chemotherapies–mAb | – | 2 (1.1) |

IMID: lenalidomide, pomalidomide, thalidomide; PI: bortezomib, carfilzomib, ixazomib; Anti-CD38 mAb: daratumumab, isatuximab; HDAC: panobinostat.

Abbreviations: 2L, second-line; 3L, third-line; 4L, fourth-line; 5L, fifth-line; BCMA, B-cell maturation antigen; D8, DREAMM-8; HDAC, histone deacetylase inhibitor; IMID, immunomodulatory drug; mAb, monoclonal antibody; PI, proteasome inhibitor
